# Technological pathways for cost-effective steel decarbonization

**DOI:** 10.1038/s41586-025-09658-9

**Published:** 2025-10-29

**Authors:** Xinyi Wu, Jing Meng, Xi Liang, Laixiang Sun, D’Maris Coffman, Andreas Kontoleon, Dabo Guan

**Affiliations:** 1 The Bartlett School of Sustainable Construction, University College London, London, UK; 2 Centre for Sustainability Science and Technology, University College London, London, UK; 3 Department of Geographical Sciences, University of Maryland, College Park, MD USA; 4 Department of Land Economy, University of Cambridge, Cambridge, UK; 5 Department of Earth System Sciences, Tsinghua University, Beijing, China

**Keywords:** Climate-change mitigation, Environmental economics

## Abstract

The iron and steel sector is central to national net-zero efforts but remains hard to abate^1,2^. Existing decarbonization roadmaps fail to guide technology choices for individual plants, given their heterogeneity and economic constraints^3–5^. Here, by integrating two global plant-level datasets and forecasted technology costs, we develop a model to identify the least-cost technology pathway for each plant worldwide in alignment with national carbon-neutrality targets. In the short term (pre-2030), energy efficiency improvements and scrap reuse are the cheapest decarbonization strategies, reducing cumulative global carbon dioxide (CO_2_) emissions by 7.8 Gt and 7.2 Gt at average costs of –US$8.5 tCO_2_^−1^ and US$0.3 tCO_2_^−1^, respectively. In the long term (after 2030), smelt reduction with carbon capture is expected to become technically mature and economically viable, achieving approximately 6.0 Gt of CO_2_ reductions at costs of US$7–15 tCO_2_^−1^ in Chinese plants and US$26–75 tCO_2_^−1^ in plants across Japan, Korea and Europe. After 2040, green-hydrogen-based steelmaking is estimated to contribute an additional 0.3 Gt of CO_2_ abatement in European plants at costs of US$27–44 tCO_2_^−1^. This study tailors plant-specific least-cost technology pathways that reconcile stakeholders’ economic interests with climate objectives, enabling actionable decarbonization strategies and supporting global net-zero targets.

## Abstract (web summary)

A model has been developed to identify the least-cost technology pathway for global individual iron and steel plants over 2020–2050 in alignment with national carbon-neutrality targets.

## Main

Combating climate change requires concerted action across all economic sectors^6^. As the largest industrial emitter, the iron and steel sector accounts for 7% of global carbon dioxide (CO_2_) emissions, with an expected increase in emissions owing to surging steel demand driven by urbanization and industrialization^7^. Decarbonizing steel is now a strategic priority, reinforced by policies such as the European Union (EU)’s Carbon Border Adjustment Mechanism, the US Inflation Reduction Act, China’s transition towards carbon management, and the net-zero commitments made by numerous nations and leading steel companies^8–11^. However, steelmaking is hard to abate because of its heavy technological dependence on fossil fuels and the significant carbon lock-in effect of long-lived facilities^7^. Traditional mitigation strategies such as energy efficiency improvements can provide only a further 15–20% emissions abatement in the future^12,13^. Meeting the carbon-neutrality target necessitates the adoption of low-carbon and zero-carbon technologies for deep decarbonization, despite their early development stage and substantial costs^1^.

A variety of decarbonization strategies have been identified, including scrap recycling^14,15^, carbon capture^16,17^, hydrogen^18,19^, bioenergy^1,20,21^, direct electrorefining^22^ and innovative additives for material efficiency improvements^8,23^. The feasibility of each option depends on technological readiness, economic viability and compatibility with existing plants and infrastructures^1,2,24^. To maintain competitiveness while reducing emissions, steel producers must assess technology costs to identify the most cost-effective pathways^25^. However, the cost of technologies often changes over time, with varying change rates between technologies, which alters the least-cost solution at different stages^26,27^. Most existing techno-economic studies on steelmaking decarbonization merely estimated the static costs of incremental^12,21,28^ or breakthrough^2,7,29–31^ technologies, while overlooking the cost dynamics over time and the readiness level of these technologies. A few studies have forecasted cost variations using methods such as the learning curve^32^ or machine learning^33^, or have based their forecasts on industrial estimates^10^, yet these often ignore regional cost disparities, focus on a single technology type or overlook plant-specific characteristics—limiting their real-world applicability. Consequently, substantial uncertainty remains over the economic viability of different technologies for individual plants worldwide in the coming decades. A global, plant-specific forecast of evolving costs across promising decarbonization options is therefore critical for designing technically and economically robust zero-carbon pathways.

Achieving net zero in the steel sector requires supporting not only policymakers but also individual plants in identifying the cost-effective, technically mature and plant-compatible decarbonization solutions^27^. However, the thousands of steel plants worldwide vary widely in processing routes, production costs, ages, locations, and access to low-carbon energy and infrastructure, resulting in substantial differences in the techno-economic feasibility of decarbonization technologies^3^. A uniform ‘one size fits all’ strategy is inadequate for addressing the unique needs of each plant and may hinder the achievement of carbon-neutrality targets^5^. Recent studies have proposed plant-level phase-out or mitigation strategies for China’s^5,34^ or the global^3,4,35^ iron and steel sector, considering plant-varied emissions, ages and locations. Yet, these works lack a comprehensive techno-economic analysis of promising decarbonization technologies and plant-specific cost forecasts, and fail to identify cost-effective technology pathways tailored to individual plants (Supplementary Note 1 and Supplementary Table 1). Compared with existing national or sectoral net-zero roadmaps optimized for minimizing total costs^25,29,36,37^, plant-level economically feasible transition pathways that focus on minimizing each plant’s production costs are more practical and encouraging for individual plants to implement.

**Extended Data Fig. 1:**
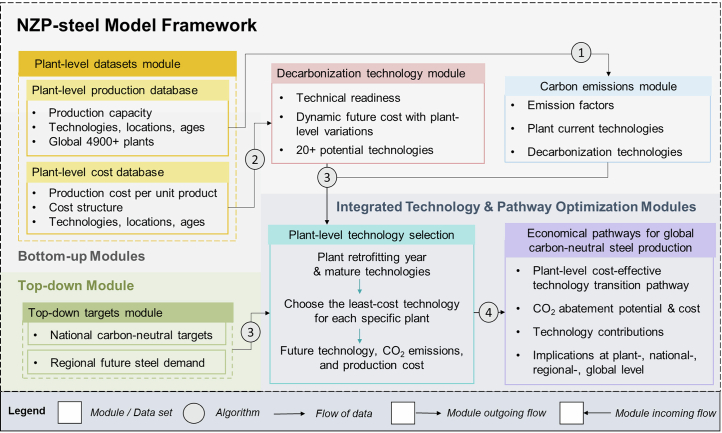
Framework of the NZP-steel model for plant-level technology pathways toward cost-effective global carbon-neutral steel production. The model comprises six modules: three bottom-up modules (plant-level datasets, decarbonization technologies, and carbon emissions), one top-down module (targets and constraints), and two integrated modules (technology selection and pathway optimization). Grey circles with numbers indicate the main algorithms, detailed in the *Methodology* section.

To fill the research gap, we develop a model to explore the plant-level net-zero pathway for the steel sector, abbreviated as NZP-steel (see Methods for details; Extended Data Fig. 1). Integrating bottom-up technology selection modules—including plant-specific technology costs, retrofitting timelines and technical feasibility—with top-down constraints such as national carbon-neutrality targets, increasing steel demand and limited scrap supply, the model tailors cost-effective technology pathways for global individual iron and steel plants over 2020–2050. These pathways reconcile stakeholders’ economic interests with climate objectives. This study provides firm data, a methodological foundation and actionable decarbonization strategies for individual plants to facilitate the achievement of net-zero-emissions targets.

## Current technologies, cost and emissions

Globally, there are nearly 4,900 operating plants in the iron and steel sector, of which 1,967 plants are responsible for 98% of global iron and crude steel production, contributing 80–90% of the sector’s CO_2_ emissions in 2021^38^. Given the various production technologies, plants producing crude steel can be further classified into four categories (Supplementary Note 2): three for steelmaking based on iron input, namely, blast furnace–basic oxygen furnace (BF–BOF), blast furnace–open hearth furnace (BF–OHF) and direct reduced iron–electric arc furnace (DRI–EAF); and steelmaking process utilizing recycled steel (Scrap–EAF).

**Fig. 1:**
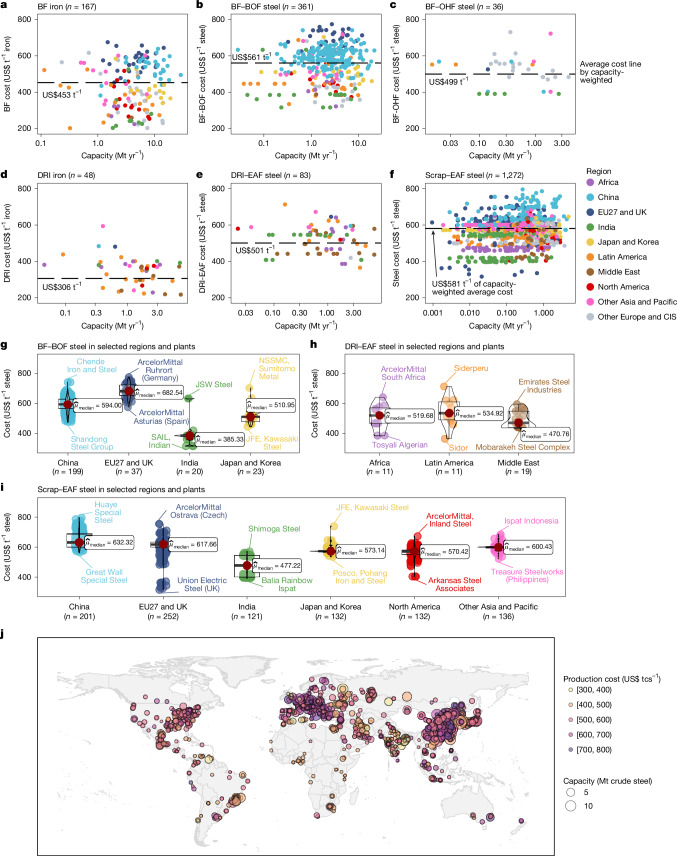
Geographical distribution of current unit production cost and capacity of global iron and steel plants. **a**–**f**, Global plants producing BF iron (**a**), BF–BOF steel (**b**), BF–OHF steel (**c**), DRI iron (**d**), DRI–EAF steel (**e**) and Scrap–EAF steel (**f**). *n* denotes the number of plants shown in each panel. Black dashed lines and accompanying cost annotations indicate the global capacity-weighted average costs for each processing route. **g**–**i**, Violin plots of BF–BOF (**g**), DRI–EAF (**h**) and Scrap–EAF (**i**) costs in key regions. Dark red points ($${\widehat{\mu }}_{{\rm{median}}}$$) denote the regional median costs, and labels indicate plants with the highest and lowest costs. CIS refers to the Commonwealth of Independent States. **j**, Geographical distribution of global steel plants with cost and capacity information. Unit production costs include operating and capital components, and are expressed as US$ t^−1^ steel (US dollars per tonne of crude steel) or US$ t^−1^ iron (US dollars per tonne of iron). Base map from https://www.naturalearthdata.com/ (public domain).

Significant cost variations exist not only among different technologies and regions but also among individual plants utilizing the same technology within a given region, highlighting the plant-level heterogeneity (Fig. 1 and Supplementary Fig. 1). Globally, cost differences across steelmaking technologies stem from their reliance on different raw materials and energy sources. The higher capacity-weighted average costs of Scrap–EAF (US$581 tcs^−1^, where tcs denotes per tonne of crude steel) and BF–BOF (US$561 tcs^−1^) are attributed to expensive scrap feedstock (US$400 tcs^−1^) and costly coke consumption (US$114 tcs^−1^), respectively, compared with the iron ore and coal used by DRI–EAF (US$501 tcs^−1^) and BF–OHF (US$499 tcs^−1^).

At the plant level, cost variations within a given technology arise from local material and energy prices, region-specific climate policies, and scale effects. Among the 1,967 iron and steel plants worldwide, 199 BF–BOF plants in China and 37 in the EU are the 2 largest steelmaking groups (36% and 7% of global steel production), but bear the highest costs, at unit production costs of US$647 tcs^−1^ (ranging from US$477 tcs^−1^ to US$741 tcs^−1^) and US$688 tcs^−1^ ($575–774 tcs^−1^) on regional average, respectively (Fig. 1b). The high costs in China and the EU are explained by the significant proportion (80%) of expensive imported iron ore used in Chinese plants, and the CO_2_ emission permit fees levied on EU plants under the EU Emissions Trading System, respectively (Supplementary Fig. 2). By contrast, Indian plants had the lowest regional average unit production cost of BF–BOF steel owing to the low cost (US$70 tcs^−1^) of local iron-ore mining and transportation.

For 83 DRI–EAF steel plants worldwide, plant-level unit production cost shows a negative correlation with plant capacity, implying the existence of a scale effect (Fig. 1e). Nearly 60% of global DRI–EAF steel was produced at a low unit cost of US$365–485 tcs^−1^ by 21 plants from the Middle East, Latin America and Africa owing to the local low price of natural gas and iron-ore pellets needed for DRI–EAF steel. For Scrap–EAF steel, the unit production cost is more or less the same at the regional level owing to the similar scrap prices across regions from the international markets, but significantly different at the plant level. Among all steelmaking technologies, Scrap–EAF has the smallest regional average cost variation of US$174 tcs^−1^ but the largest individual plant cost variation of US$480 tcs^−1^.

**Fig. 2:**
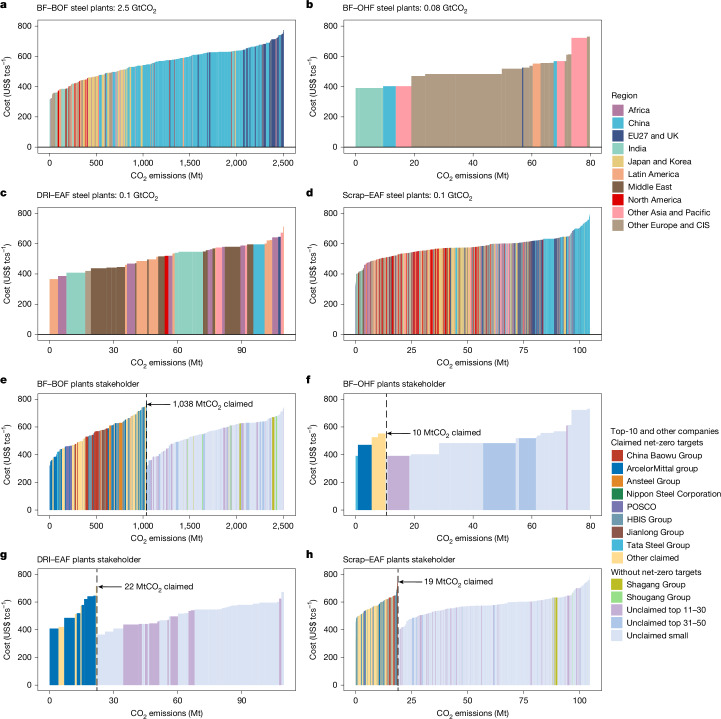
CO_2_ emissions and unit production cost of global steel plants by region and ownership. **a**–**d**, BF–BOF (**a**), BF–OHF (**b**), DRI–EAF (**c**) and Scrap–EAF (**d**) plants, coloured by region. **e**–**h**, BF–BOF (**e**), BF–OHF (**f**), DRI–EAF (**g**) and Scrap–EAF (**h**) plants re-coloured by ownership, grouped into 14 categories: 8 of the top-10 companies and smaller companies with carbon-neutrality targets, the remaining 2 of the top-10 companies, companies ranked 11–30 and 31–50, and those ranked beyond 50 without neutrality commitments. Each panel presents all global steel plants using the specified processing route, ordered by unit production cost.

Figure 2 shows the unit production cost and CO_2_ emissions of global iron and steel plants. In 2021, plants from China, EU27 and the UK, and Japan and Korea contributed 51%, 11% and 10% of the 2.8 GtCO_2_ from the global steel sector, respectively (Fig. 2a–d). Steel production by Chinese plants was both expensive and emissions intensive, whereas Indian plants had the lowest cost but the highest CO_2_ intensity, and plants in North America had the least-emissions production with moderate cost (Extended Data Fig. 3). In terms of climate mitigation commitments, 8 of the largest 10 steel companies and 7 smaller companies have pledged to reach carbon neutrality by 2030–2050, including a total of 296 steel plants that currently account for 39% of global steelmaking emissions (Fig. 2e–h). The other 1,436 plants from companies without pledged climate goals, such as Shagang Group (47 MtCO_2_, representing 1.7% of global emissions) and Shougang Group (40 MtCO_2_, 1.4%), should also expedite decarbonization efforts to align with national carbon-neutrality targets as soon as possible.

## Future cost of decarbonization technologies

**Extended Data Fig. 2:**
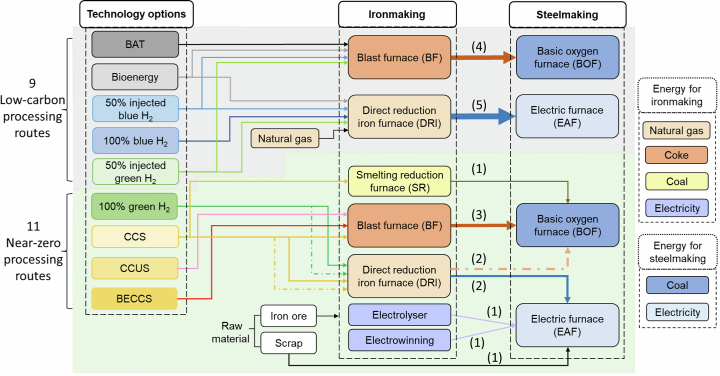
Toolbox of promising decarbonization technologies for low- and zero-carbon steel production. Numbers in parentheses and line thicknesses between ironmaking and steelmaking furnaces indicate the number of mitigation options available for each processing route. Detailed descriptions of these technologies are provided in Extended Data Table 1.

To simultaneously meet the steel demand and carbon mitigation target, we have developed a decarbonization toolbox containing 20 promising steelmaking technologies^2,10,19,28,29,39^ (Extended Data Fig. 2 and Extended Data Table 1). The toolbox includes 9 low-carbon techniques involving energy efficiency improvements, and partial replacement of fossil fuels with injected hydrogen (H_2_) or bioenergy; and 11 near-zero-emissions technologies including recycled steelmaking (that is, scrap), carbon capture and storage (CCS), complete utilization of green H_2_, and direct electrolysis. Because most of these technologies have not been commercialized yet and lack historical cost data, we have combined component-based learning curves^16,32,40^ with plant-level current cost databases to forecast the plant-specific future costs of 20 decarbonization technologies (see Methods for details). Overall, all costs will decline over time because of technological progress. However, there are big differences in terms of technical readiness and production costs across technologies.

**Fig. 3:**
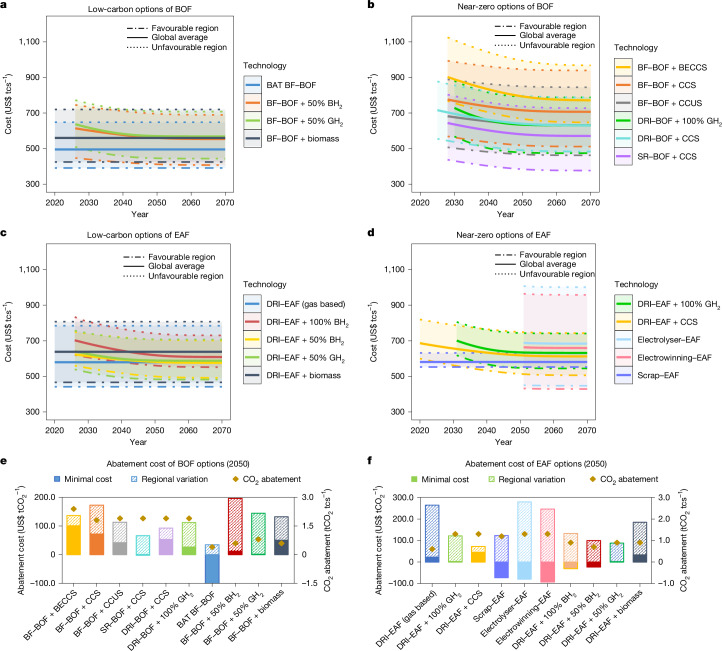
Future production and abatement cost estimates of promising steelmaking technologies. **a**–**d**, Global unit production cost per tonne crude steel (US$ tcs^−1^) for low-carbon BOF steelmaking (**a**), near-zero BOF steelmaking (**b**), low-carbon EAF steelmaking (**c**) and near-zero EAF steelmaking (**d**). Among technologies, BH_2_ refers to blue hydrogen, GH_2_ to green hydrogen, CCS to carbon capture and storage, BECCS to bioenergy with carbon capture and storage, and CCUS to carbon capture, utilization and storage. The solid lines indicate the global-average cost, and the dashed and dash-dotted lines denote the lowest and highest regional costs, respectively. **e**,**f**, The minimum CO_2_ abatement cost (US$ tCO_2_^−1^, bars) and abatement potential per tonne crude steel (tCO_2_ tcs^−1^, dots) for BOF-based (**e**) and EAF-based (**f**) options, compared with conventional BF–BOF and DRI–EAF production in 2050. Shaded areas in **a**–**f** represent regional cost variations between maximum and minimum values.

For existing BF–BOF steel plants, improving energy efficiency is the earliest-maturing and cheapest low-carbon option in the short term (before 2030). A plant applying comprehensive efficiency measures, such as top gas recovery, enhanced heat efficiency, and increased use of scrap and pulverized coal injection, is named the best-available technology (BAT) BF–BOF. Owing to energy savings, such efficiency improvements will result in a production cost decrease of US$20 tcs^−1^ and a CO_2_ abatement cost of −US$50 tCO_2_^−1^ on average across plants globally (Fig. 3a,e). In the long term (after 2030), as deep decarbonization technologies mature, smelt reduction with CCS (SR–BOF + CCS) will be the most economical zero-carbon option for plants in most regions, whereas direct reduction with green H_2_ (DRI–BOF + 100% GH_2_) will be cost-competitive for plants in the EU, Latin America and the Pacific after 2040 (Extended Data Fig. 4). The global-average CO_2_ abatement cost will be US$63 tCO_2_^−1^ for SR–BOF + CCS and US$110 tCO_2_^−1^ for DRI–BOF + 100% GH_2_ in 2030 and decrease to US$36 tCO_2_^−1^ and US$63 tCO_2_^−1^ by 2050, respectively (Extended Data Fig. 5), with most values below the EU’s carbon price of US$96 tCO_2_^−1^ in 2023^41^.

For existing DRI–EAF steel plants that consume either coal or natural gas, not only low-carbon options but also CCS application will be technically feasible before 2025. In the short term, the cheapest alternative for coal-based DRI–EAF plants is switching to natural gas with a US$46 tcs^−1^ cost increase globally, whereas CCS deployment (DRI–EAF + CCS) is the most cost-effective option for those already gas-based plants incurring a cost increase of US$67 tcs^−1^ (Fig. 3c,d). In the medium to long term, given the rapid decline of green H_2_ price, full fuel substitution with green H_2_ (DRI–EAF + 100% GH_2_) is expected to become more affordable than CCS for plants in the EU27 and UK, and China before 2035, and in India and other Asian and Pacific countries around 2040, resulting in cost increases of US$3–86 tcs^−1^ by 2050 (Extended Data Fig. 4). Meanwhile, from the 2030s, partial injection of green H_2_ (DRI–EAF + 50% GH_2_) becomes an economic transitional low-carbon option in EU, Latin America, India and the Pacific region, with estimated cost increases of US$36–53 tcs^−1^ by 2050. By the mid-century (after 2050), the commercialization of direct electrified steelmaking technologies (that is, electrolyser–EAF and electrowinning–EAF) promises cost advantages over DRI–EAF with CCS and green H_2_ in regions such as India and the Middle East, with production costs ranging from US$433 tcs^−1^ to US$560 tcs^−1^ (Fig. 3d).

Scrap–EAF is both technically and economically ideal for decarbonization because it is currently the only mature near-zero-emissions technology with affordable cost. In the EU and Pacific, its cost advantage and lower emissions compared with BF–BOF and DRI–EAF result in negative CO_2_ abatement costs of −US$46 tCO_2_^−1^ and −US$73 tCO_2_^−1^, respectively (Fig. 3f). However, the limited scrap supply constrains the growth potential of Scrap–EAF worldwide^7^, necessitating the plants without scrap availability to turn to CCS or H_2_ for deep decarbonization. Furthermore, cost forecast here can be influenced by factors such as prices of key materials and energy (that is, scrap, iron ore, coke and electricity), along with initial costs, learning rates and cumulative capacities of decarbonization components (Extended Data Figs. 8 and 9, Supplementary Notes 4–7, and Supplementary Figs. 4–15).

## Plant-level least-cost transition pathway

**Fig. 4:**
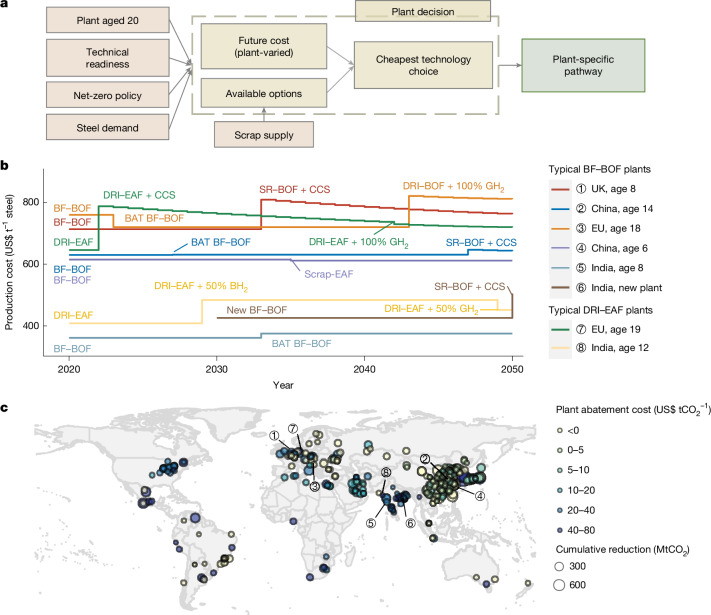
Abatement potential and cost of individual steel plants under the least-cost transition pathway in the medium deployment scenario. **a**, Framework used to explore plant-specific least-cost transition pathways worldwide. Pink indicates key constraints in the pathway exploration, yellow represents factors influencing plant-level least-cost technology choices, and green denotes the final selected transition pathway. **b**, Least-cost technology transition pathways and production cost changes of typical BF–BOF and DRI–EAF plants of different operating ages in China, the EU and India. A sudden increase or decrease in the line reflects the cost changes from retrofitting to a new technology, whereas a smooth decline indicates gradual cost reduction over time for the same technology. **c**, Geographical distribution, average abatement cost and cumulative CO_2_ abatement of global steel plants over 2020–2050. Base map from https://www.naturalearthdata.com/ (public domain).

Individual plants, balancing economic returns with climate goals, typically adopt the lowest-cost option available under prevailing external conditions^25^. On the basis of this feature, we developed a model to design the least-production-cost, plant-level decarbonization pathway. The model assumes that each plant will choose the cheapest technology available at its retrofit window (every 20 years^7^), subject to constraints imposed by national carbon-neutrality targets, future steel demand, limited scrap supply, technology maturity and policies of varying decarbonization paces (Fig. 4a and Supplementary Fig. 3).

**Fig. 5:**
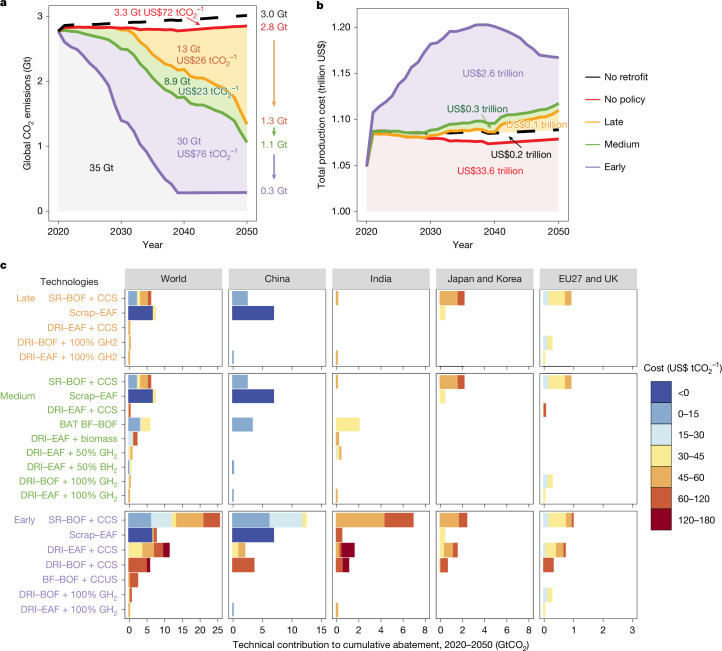
Comparison of CO_2_ abatement potentials, abatement costs and technology contributions under different policy scenarios. **a**,**b**, Global CO_2_ emissions and production costs of all steel plants during 2020–2050 under the least-cost pathways with varying policy strengths (late, medium and early). The numbers in yellow, green and purple indicate differences between adjacent scenarios: cumulative CO_2_ abatement and average abatement cost (**a**) and increases in total production cost (**b**), corresponding to the areas between neighbouring solid lines. **c**, Abatement potentials and abatement costs of different decarbonization technologies worldwide and in major regions over 2020–2050 under the late, medium and early scenarios, compared with reference pathways without policy intervention.

Without policy intervention, only a few steel plants worldwide would cut emissions through energy efficiency improvements (that is, BAT BF–BOF) with negative costs, which offset part of emissions increase from expanded steel production but lead to 2.8 GtCO_2_ emissions in 2050 (Fig. 5a). To avoid this, policy intervention forcing deep decarbonization is necessary. We start with a medium deployment scenario, which requires global steel plants to adopt either low-carbon or zero-carbon technologies from their first retrofit and to deploy only zero-carbon technologies at their last retrofit before reaching the national carbon-neutrality target years.

Figure 4b illustrates how plant heterogeneity in technology types, production costs, equipment ages and national net-zero policies lead to distinct plant-level optimal transition pathways and cost changes, using six BF–BOF plants and two DRI–EAF plants (aged 1–19) from the EU, China, and India as examples. Specifically, all steel plants in the EU should deploy zero-carbon options like DRI–BOF + 100% GH_2_ or SR–BOF + CCS by 2050 despite substantial cost increases (plant 1 and plant 3), whereas plants in China under the age of 10 and all plants in India could use low-carbon technologies until then (plant 5). In China, BF–BOF plants over 10 years old have two retrofit opportunities before 2050 and would transition to the cheapest low-carbon technology BAT BF–BOF for the first retrofit in the 2020s and deploy the cheapest zero-carbon option SR–BOF + CCS at the second retrofit in 2040s (plant 2). Some plants benefit from increased scrap supply, enabling a switch to Scrap–EAF with lower costs (plant 4). Newly built BF–BOF plants in India will transition to green H_2_ steelmaking from 2050 (plant 6). For DRI–EAF plants, those with earlier retrofit schedules and near-term neutrality targets adopt CCS or blue H_2_ (ref. ^39^), given their readiness and short-term cost-effectiveness, whereas those with later reconstruction timelines shift directly to green H_2_—either fully or partially—once costs decline (plant 7 and plant 8).

Following the same process, we identify plant-specific technology solutions and transition pathways globally. This enables the calculation of individual plant abatement potential and cost, and shows that several plants exhibit low abatement costs (Fig. 4c). Globally, plant-level average abatement costs over 2020–2050 vary depending on current processing routes, ranging from −US$80 tCO_2_^−1^ to US$66 tCO_2_^−1^ for BF–BOF plants, US$15–65 tCO_2_^−1^ for DRI–EAF plants, and remain unchanged for existing near-zero-emissions Scrap–EAF plants (Extended Data Fig. 6).

## Global least-cost decarbonization pathways

Countries with ambitious climate goals prefer early rollout of zero-carbon technologies, whereas those prioritizing economic development may postpone mitigation actions to avoid the burden of substantial cost. To quantify the impact of climate policy stringency on global decarbonization pathways, we develop two additional scenarios: an early deployment scenario mandating zero-carbon technologies from the very first retrofit of all plants, and a late deployment scenario allowing plants to keep current technologies until the last retrofit before national carbon neutrality. All scenarios require zero-carbon technologies for the final retrofit to avoid stranded assets upon reaching carbon neutrality.

Figure 5 compares the global abatement potential, cost and technology contributions across the three scenarios based on aggregated plant-level pathways. All scenarios achieve substantial emissions reductions: global CO_2_ declines from 2.8 Gt in 2020 to 1.3 Gt (late), 1.1 Gt (medium) and 0.3 Gt (early) in 2050 (Fig. 5a). China’s emissions fall by 90–92% across all scenarios (from 1.4 Gt in 2020 to 0.10–0.12 Gt in 2050), whereas the EU and Japan consistently reach net-zero by 2050. By contrast, regions lacking mid-century carbon-neutrality targets, such as India and the Middle East, show larger variation in plant-level technology deployment, driving differences in global outcomes. The medium deployment scenario emerges as the most cost-effective, delivering a cumulative 22.4 GtCO_2_ reduction (2020–2050) at a global-average abatement cost of US$24.7 tCO_2_^−1^—lower than the late (13.5 Gt at US$26.0 tCO_2_^−1^) and early (52.7 Gt at $54.0 tCO_2_^−1^) scenarios. This cost advantage arises from the optimal use of mature low-carbon technologies as transitional measures before zero-carbon options become commercially viable. The medium deployment’s superiority is consistent across most sensitivity analyses (Supplementary Note 8 and Supplementary Figs. 16–28).

Technology contributions to global abatement potential and cost also vary among the three scenarios (Fig. 5c). In the late scenario, most reductions come from Scrap–EAF (7.2 Gt) and SR–BOF + CCS (6.0 Gt), with global-average abatement costs of US$0.3 tCO_2_^−1^ and US$33 tCO_2_^−1^. Scrap–EAF is the cheapest zero-carbon option in most regions, but only 45 plants in China and 2 in Japan can switch from BF–BOF to Scrap–EAF owing to limited scrap supply. SR–BOF + CCS deeply decarbonizes 235 BF–BOF plants worldwide and will contribute considerable CO_2_ mitigations in China (2.4 Gt, 149 plants), Japan and Korea (2.1 Gt, 21 plants), and the EU27 and UK (0.9 Gt, 17 plants). The abatement cost of this technology increased 4–5 times in Europe (US$26–52 tCO_2_^−1^), and Japan and Korea (US$46–75 tCO_2_^−1^) compared with China (US$7–15 tCO_2_^−1^), owing to the higher price of CCS in the former regions^39^. Green H_2_ deployment is minimal and geographically concentrated—yielding 0.3-Gt carbon reduction through DRI–BOF + 100% GH_2_ and DRI–EAF + 100% GH_2_ at costs of US$27–44 tCO_2_^−1^ in 21 EU plants and US$2–54 tCO_2_^−1^ in 5 plants elsewhere.

In the medium scenario, retrofitting existing BF–BOF plants with the BAT abates 5.6 Gt of CO_2_—26% of the scenario’s total—at an average cost of just US$15 per tonne. This is achieved by upgrading 251 plants by 2040, primarily in China, India, Japan and Korea. This pathway offers the lowest abatement cost of any scenario, highlighting the critical role of mature technologies as a cost-effective bridge to zero-carbon steelmaking. Furthermore, these upgrades are cost-saving for some plants and thus implemented profitably even without policy (Extended Data Fig. 7). Including such cost-negative actions raises the CO_2_ abatement potential to 7.8 Gt and reduces the cost to –US$8.5 tCO_2_^−1^ relative to existing technology patterns.

In the early scenario, mandatory early transition to zero-carbon technologies would drive extensive CCS deployment, abating nearly 42 Gt of cumulative CO_2_ emissions (80% of scenario total) at US$52 tCO_2_^−1^. Compared with the late scenario, the earlier and large-scale deployment of CCS (that is, SR–BOF + CCS, DRI–BOF + CCS and DRI–EAF + CCS) will contribute an additional abatement of 36 GtCO_2_ globally but increase the plant-level abatement cost from US$7–75 tCO_2_^−1^ to US$7–174 tCO_2_^−1^. Growing scrap availability in India leads to the expansion of Scrap–EAF but at a relatively higher cost of US$107 tCO_2_^−1^ than China and Japan.

## Plant-specific strategy for carbon neutrality

At the plant level, higher-cost climate actions are rarely adopted voluntarily, making net-zero targets unattainable without targeted incentives. Policies must therefore encourage individual steel plants to decarbonize while minimizing costs. Among different technology strategies, the medium pathway—emphasizing transitional low-carbon technologies—achieves the lowest affordable average abatement cost of US$22 tCO_2_^−1^ during 2020–2050. This least-cost pathway suggests region-specific priorities. China could leverage its low-cost advantages in scrap recycling and CCS; the EU could exploit its technological and cost leadership in H_2_ to accelerate commercial deployment of green H_2_ steelmaking; and India, facing rapid production growth that drives emissions to 500 MtCO_2_ in 2050 even with low-carbon technologies, must initiate a zero-emissions transition earlier. For India, this could begin in the 2040s, once SR–BOF + CCS and DRI–BOF + 100% GH_2_ become mature and cost-competitive^42^, to avoid a steep post-2050 mitigation burden.

## Leveraging the role of energy efficiency

**Extended Data Fig. 3:**
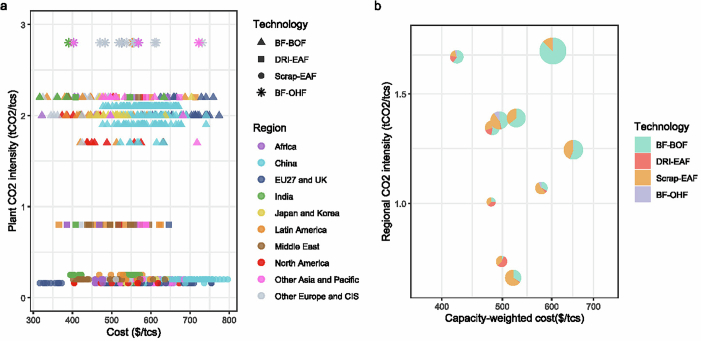
Relationship between CO_2_ intensity and production cost in global steel plants. a) CO_2_ intensity and production cost per ton of crude steel for individual plants, with variations in processing technologies shown by point shape and regional differences by color. b) Regional averages of CO_2_ intensity and capacity-weighted production cost, with pie charts illustrating the contributions of different processing technologies to total regional CO_2_ emissions. Colors denote technology types, and pie size reflects regional capacity.

**Extended Data Fig. 4:**
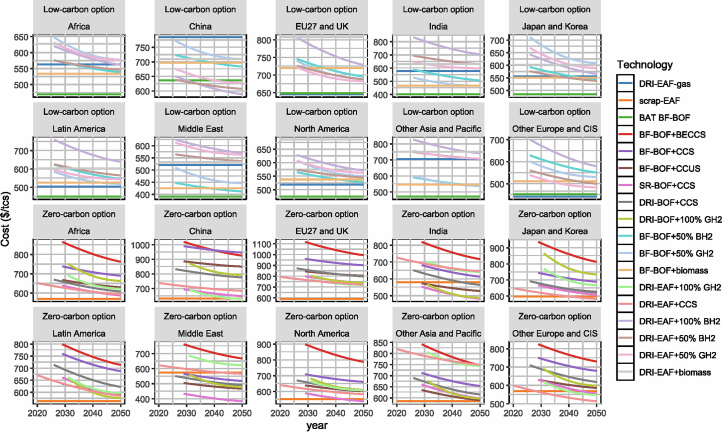
Regional production cost estimates of promising steelmaking technologies. Technologies are grouped into two categories: low-carbon and near-zero emissions. For each region, the cost line represents the capacity-weighted average production cost across individual steel plants.

**Extended Data Fig. 5:**
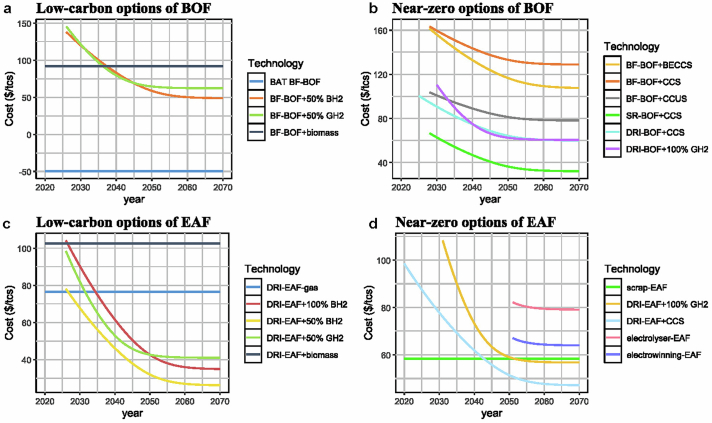
Carbon abatement cost estimates of low-carbon and near-zero emission steelmaking technologies. a–b) Low-carbon and near-zero technology options for BF-BOF retrofits, respectively. Abatement costs are calculated relative to the carbon emissions and production cost of AVG BF-BOF, representing the current BF-BOF route at the global average level. c–d) Low-carbon and near-zero technology options for DRI-EAF retrofits, respectively. Abatement costs are calculated relative to the emissions and cost of the current global-average DRI-EAF route.

**Extended Data Fig. 6:**
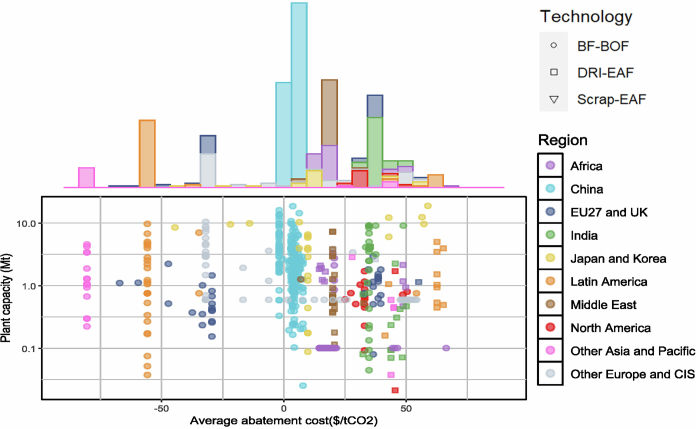
Average abatement cost of individual steel plants over 2020–2050 in the medium deployment scenario. Colors indicate regions, while different point shapes denote technology types. The bar plot shows total production capacity in 2050 for plants grouped by similar average abatement costs.

**Extended Data Fig. 7:**
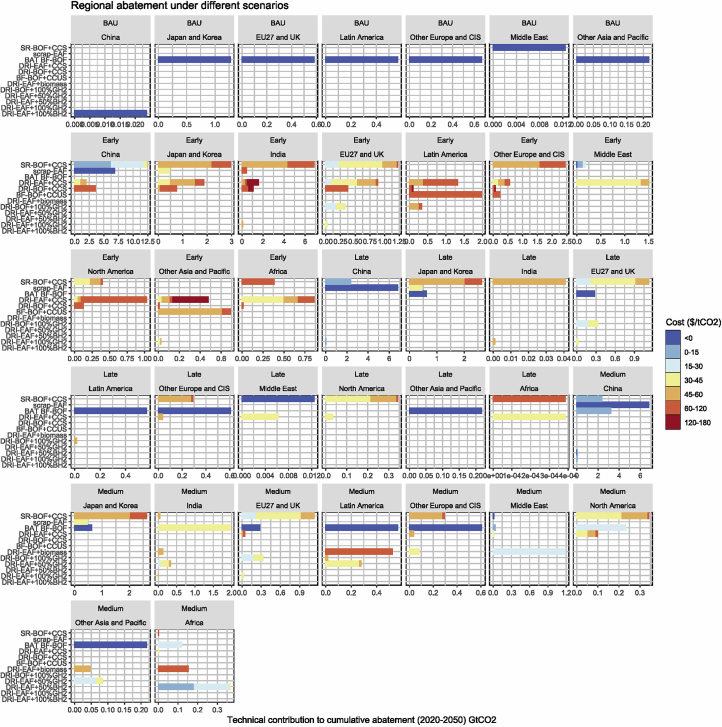
Regional abatement potential and abatement cost of cost-effective technology pathways over 2020–2050. Values are calculated relative to pathways preserving current technology configurations.

**Extended Data Fig. 8:**
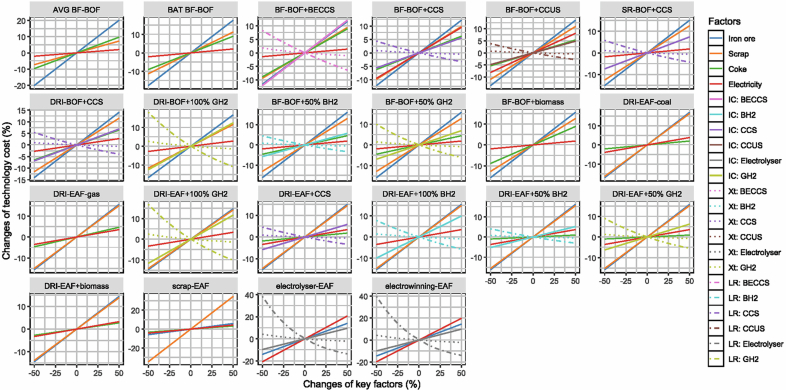
Cost sensitivity of 22 technologies to key input prices, initial costs, learning rates, and component capacities by 2050. Price fluctuations in iron ore, scrap, coke, and electricity are considered as four primary cost drivers in steelmaking. Variations in initial cost (IC), learning rate (LR), and cumulative capacity (Xt) are also examined for novel components, including carbon capture and storage (CCS), bioenergy with carbon capture and storage (BECCS), carbon capture, utilization and storage (CCUS), green hydrogen (GH_2_), blue hydrogen (BH_2_), and electrolysers. Colors differentiate input types and novel components, while line styles denote parameter categories: solid lines indicate input prices or initial costs, dot-dashed lines represent learning rates, and dotted lines show cumulative capacities.

**Extended Data Fig. 9:**
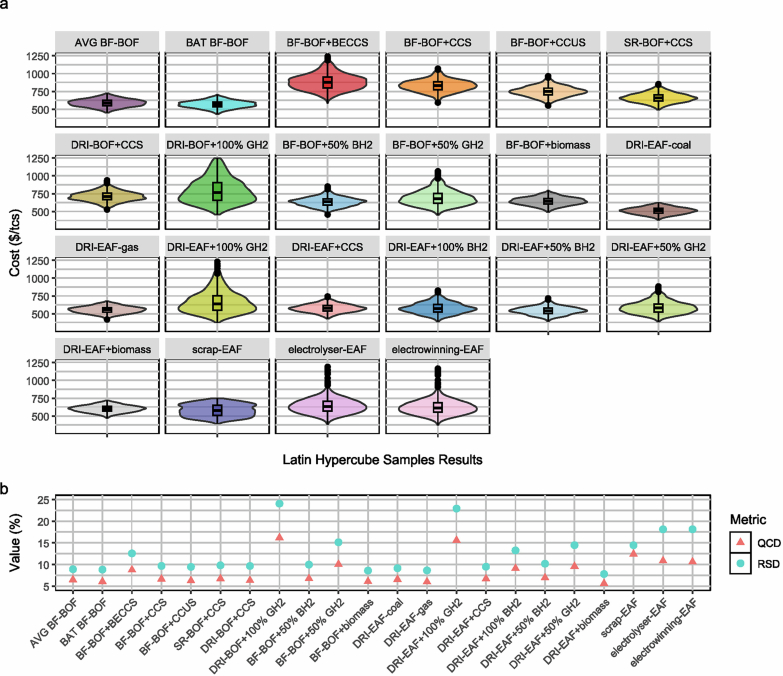
Multi-factor sensitivity analysis of 2050 cost estimates for 22 technologies. All 22 cost drivers identified in Table S5 were simultaneously varied in the 1,000 cost combinations drawn by the Latin-hypercube sampling method from the input space (10^44^ possible combinations). a) Box-and-whisker plots display the median (line), interquartile range (box), and 5th–95th percentiles (whiskers) for each technology, with violin plots representing the probability density of cost distributions. b) Statistical summary of a), reporting the relative standard deviation (RSD) and quartile coefficient of dispersion (QCD) for each technology. Both RSD and QCD values are shown as percentages in this figure.

Even the same conventional technology (that is, BF–BOF) performs differently, from a technical standpoint, across different plants, resulting in varied carbon emission intensities (Extended Data Fig. 3). Countries should focus on standardizing technical performance across plants and improving current energy efficiency and operational management to the best possible level, as a decarbonization low-hanging fruit^43^. For instance, the BAT for BF–BOF (that is, BAT BF–BOF), which can be attained by optimizing feedstock ratios and reaching the highest efficiency levels of advanced plants, is technically and economically feasible for most emissions-intensive plants. If all steel plants prioritize this technology as their decarbonization choice, 7.8 GtCO_2_ can be cumulatively abated, which accounts for 31% of global abatement in the medium deployment scenario compared with the scenario without retrofitting, at a negative average abatement cost of −US$8.5 tCO_2_^−1^ (Extended Data Fig. 7).

## Financing the high costs of decarbonization

Achieving deep decarbonization in the industrial sector is a costly and technology-intensive endeavour. Even in the late deployment scenario with the lowest total abatement cost, nearly US$350 billion is needed to reduce 13 GtCO_2_ emissions during 2020–2050, in which Japan and Korea steel plants will bear the heaviest economic burden of US$221 billion for 2.4 GtCO_2_ abatement, whereas plants in the EU27 and UK, China, and India will spend US$84 billion for 1.2-GtCO_2_, US$6.7 billion for 9.2-GtCO_2_ and US$2.0 billion for 0.04-GtCO_2_ abatement, respectively (Fig. 5). Such massive abatement costs are unaffordable for many steel plants with limited profits and may lead to their closure. To avoid the financial shock of a net-zero transition and maintain the pillar role of the iron and steel industry, timely and generous financial assistance from the government is necessary. For instance, the UK government granted £300 million each to British Steel and Tata Steel to promote their transition to green steel production^44^. The EU Green Innovation Fund supported research and development and risk-sharing for large-scale green steel demonstrations^45^.

## Methods

### Plant-level pathway design model

This study developed a plant-level net-zero pathway model for the steel sector, called NZP-steel, to identify cost-effective technology pathways for each plant worldwide. The model integrates bottom-up modules built on plant-level datasets with top-down constraints, including national carbon-neutrality targets, rising steel demand and limited scrap supply.

The model consists of six modules (Extended Data Fig. 1). Three bottom-up modules are used to: (1) compile two plant-level industry datasets (yellow box), (2) calculate carbon emissions by plant and technology (blue box), and (3) estimate dynamic costs of decarbonization technologies (pink box). The algorithms for these modules are described below. The top-down module (green box) incorporates scenario constraints for plant-level pathway exploration, including national net-zero targets, future steel demand and scrap availability, sourced from the literature^7,46^. Using plant-level cost and emission data from the bottom-up modules together with top-down constraints, the model determines the least-cost technology choice for each plant (cyan box). The resulting plant-level pathways are then aggregated to national, regional and global levels to quantify overall CO_2_ abatement potentials and costs (purple box).

### Plant-level datasets of global steel production and cost

The two plant-level databases used in this study are the World Crude Steel Capacity and Production Database^38^ and the Global Iron and Steel Cost Database^47^.

The World Crude Steel Capacity and Production Database^38^ (https://www.steelonthenet.com/plant.html) covers more than 4,900 operating plants worldwide with a total of more than 20,000 facilities in 127 countries. Of these, 1,967 plants are involved in the production of iron and crude steel, whereas the others are steel processing plants that are excluded from this study owing to their limited emissions and lack of decarbonization measures. The database contains facility-based information on processing routes, nominal capacities, start-up and retrofitting years, plant geographical locations, and ownership in the year 2018. The annual production of each plant has been updated for the years 2020 and 2021 according to the national steel production data from the World Steel Association (WSA) and the plant-level cost database below^48^. This production database provides original information on technology types, capacity and operating ages of individual plants, which contributes to the differentiation of plant-specific decarbonization pathways.

The Global Iron and Steel Cost Database in this study is compiled and harmonized from the open database TransitionZero^47^ (https://www.transitionzero.org/products/global-steel-cost-tracker) and the non-public Metalinfo steel cost database (http://www.metalinfo.cn). Access to the latter requires contacting the online customer service to purchase the database. The merged cost database encompasses the plant-level production costs of different processing routes for 1,082 global iron and steel plants in 2021. Cost information includes not only the total production cost per tonne of iron or crude steel but also four cost subcategories, that is, cost of raw materials, cost of energy and reductants, cost of labour and overheads, and capital charges. The database also provides geographical locations for each plant, facilitating the mapping between the cost database and the production database. Owing to data limitations, a group of small plants included in the production database are not covered in the cost database, accounting for 13% of global steel production^48^. The production costs of these plants are estimated using the national capacity-weighted average costs for the same processing routes, ensuring consistency with the average costs of the majority of steel plants. We acknowledge that this approximation may introduce some uncertainty; however, it is a feasible approach given the limited availability of plant-level cost data.

### CO_2_ emissions of current and decarbonization technologies

The CO_2_ emissions of current processing routes and decarbonization technologies are estimated using the mass balance approach of the WSA^10,49^. Compared with the Intergovernmental Panel on Climate Change method, which requires historical emission factors measured from existing activities, the WSA’s approach based on the carbon balance of material flows is more suitable for estimating future emissions of yet-to-be-commercialized decarbonization technologies lacking empirical data^7,28,49,50^. This method also enables emission estimation at the level of individual processes or facilities.

As shown in equation (1), the total CO_2_ emissions of a steel plant are calculated as the sum of process-level emission intensities, each expressed per tonne of crude steel, multiplied by the plant’s crude steel output. The emission intensity of each process is obtained from the carbon content difference between purchased inputs and sold outputs, converted into CO_2_ equivalents (equation (2)).

1 $${E}_{{\rm{t}}{\rm{o}}{\rm{t}}{\rm{a}}{\rm{l}}}=P\times \mathop{\sum }\limits_{i=1}^{n}{{\rm{E}}{\rm{I}}}_{i}$$

2 $${{\rm{E}}{\rm{I}}}_{i}=\mathop{\sum }\limits_{j,k=1}^{n}({C}_{i,{\rm{p}}{\rm{u}}{\rm{r}}{\rm{c}}{\rm{h}}{\rm{a}}{\rm{s}}{\rm{e}}{\rm{d}},j}\times {{\rm{E}}{\rm{F}}}_{j}-{C}_{i,{\rm{s}}{\rm{o}}{\rm{l}}{\rm{d}},k}\times {{\rm{E}}{\rm{F}}}_{k})$$

where *E*_total_ is the total CO_2_ emissions of a steel plant, *P* is the total crude steel output of the plant, EI_*i*_ is the emission intensity of process *i*, normalized per tonne of crude steel, *C*_*i*__,purchased,__*j*_ is the amount of purchased feedstock *j* required in process *i*, normalized per tonne of crude steel, *C*_*i*,sold,*k*_ is the amount of sold product *k* from process *i*, normalized per tonne of crude steel, and EF_*j*_ and EF_*k*_ are the emission factors for items *j* and *k*, derived from the carbon content of materials using the stoichiometric ratio (44/12) to convert C into CO_2_.

Calculations in this study incorporate Scope 1 and Scope 2 emissions. Scope 1 CO_2_ emissions from process *i* include direct emissions from site chimneys (*E*_*i*,site_) and CO_2_ credits (*E*_*i*,credit_) from on-site steam generation, as shown in equation (3). Scope 2 emissions refer to the indirect upstream emissions (*E*_*i*,upstream_) related to electricity procurement (equation (4)).

3 $${E}_{i,{\rm{s}}{\rm{c}}{\rm{o}}{\rm{p}}{\rm{e}}1}={E}_{i,{\rm{s}}{\rm{i}}{\rm{t}}{\rm{e}}}-{E}_{i,{\rm{c}}{\rm{r}}{\rm{e}}{\rm{d}}{\rm{i}}{\rm{t}}}=P\times \mathop{\sum }\limits_{i=1}^{n}{{\rm{E}}{\rm{I}}}_{i,{\rm{s}}{\rm{c}}{\rm{o}}{\rm{p}}{\rm{e}}1}$$

4 $${E}_{i,{\rm{s}}{\rm{c}}{\rm{o}}{\rm{p}}{\rm{e}}1+2}={E}_{i,{\rm{s}}{\rm{c}}{\rm{o}}{\rm{p}}{\rm{e}}1}+{E}_{i,{\rm{u}}{\rm{p}}{\rm{s}}{\rm{t}}{\rm{r}}{\rm{e}}{\rm{a}}{\rm{m}}}=P\times \mathop{\sum }\limits_{i=1}^{n}{{\rm{E}}{\rm{I}}}_{i,{\rm{s}}{\rm{c}}{\rm{o}}{\rm{p}}{\rm{e}}1+2}$$

Here process-based emission intensities for Scope 1 (EI_*i*,scope1_) were collected from International Environmental Agency and previous studies^3,4,7,10^, whereas emission intensities (EI_*i*,scope1+2_) for each process in Scope 1 and Scope 2 were obtained from the WSA^49,51^. The primary results in this study are based on Scope 1 emissions, whereas the impact of Scope 1 + 2 emissions are discussed in the sensitivity analyses (Supplementary Note 8 and Supplementary Table 7). The input and output flows (*C*_*i*_) of current processing routes such as BF–BOF, DRI–EAF and Scrap–EAF were obtained from the WSA^52^, whereas those of 20 promising decarbonization technologies were collected from other literature^2,10,52^ (Extended Data Table 1). Our estimates of emission intensities for existing steel plants and decarbonization technologies are comparable to previous research.

### Future cost estimates of decarbonization technologies

A toolbox of 20 promising low-carbon and zero-carbon technologies for net-zero steel production has been developed^2,7,10,28^ (Extended Data Fig. 2 and Extended Data Table Table 1). Estimating plant-specific dynamic costs of these options is essential to identify the least-cost technology solution for each plant. However, the early stage of most decarbonization technologies lack historical cost and capacity data, challenging traditional learning curve methods for cost forecast^16^. Therefore, we innovatively integrated global plant-level production and cost databases with a component-based cost forecasting model^16,53^ and learning curves^32,40^ of decarbonization components to project the plant-specific future costs for 20 decarbonization technologies (Supplementary Fig. 29).

According to the component-based method, the cost of a complex technology can be decomposed into the costs of its individual components at different levels of maturity^16^. For steel plants, conventional processing routes (that is, BF–BOF, DRI–EAF, Scrap–EAF and so on) have been commercialized for decades, with costs primarily driven by fluctuations in raw material and energy prices rather than by technological progress^47^. By contrast, the technical maturity and future costs of novel decarbonization components (that is, CCS, carbon capture, utilization and storage (CCUS), bioenergy with carbon capture and storage (BECCS), H_2_ and so on) in steel plants may change significantly with cumulative deployment experience and across regions^10,32,53^. Therefore, the future production cost (*Y*) of a steel plant adopting a specific technology (*k*) can be divided into two parts:

5 $${Y}_{{\rm{p}}{\rm{l}}{\rm{a}}{\rm{n}}{\rm{t}},k}={Y}_{{\rm{r}}{\rm{o}}{\rm{u}}{\rm{t}}{\rm{e}},k,i}+{Y}_{{\rm{n}}{\rm{o}}{\rm{v}}{\rm{e}}{\rm{l}},k,j}$$

where, *Y*_plant_, *Y*_route_ and *Y*_novel_ represent the unit production costs (US$ tcs^−1^) of the overall plant, processing routes and novel components, respectively; and *i* and *j* denote the types of conventional processing routes and novel components, respectively.

The cost of a processing route (*Y*_route_) consists of four parts: the cost of raw materials (*Y*_raw material_), including iron ore, scrap and coke; the cost of energy (*Y*_energy_), such as thermal coal and electricity; the cost of labour and overheads (*Y*_labour_); and the capital cost (*Y*_capital_), as shown in equation (6). Plant-specific costs for each processing route across the 20 decarbonization options were calculated based on our plant-level cost database (Supplementary Note 3).

6 $${Y}_{{\rm{r}}{\rm{o}}{\rm{u}}{\rm{t}}{\rm{e}},k,i}={Y}_{{\rm{raw\; material}},k,i}+{Y}_{{\rm{e}}{\rm{n}}{\rm{e}}{\rm{r}}{\rm{g}}{\rm{y}},k,i}+{Y}_{{\rm{l}}{\rm{a}}{\rm{b}}{\rm{o}}{\rm{u}}{\rm{r}},k,i}{+Y}_{{\rm{c}}{\rm{a}}{\rm{p}}{\rm{i}}{\rm{t}}{\rm{a}}{\rm{l}},k,i}$$

The cost of novel decarbonization components (*Y*_novel_) was estimated using Wright’s law, postulating that the cost of technology (*y*_*t*_) evolves as a function of cumulative capacity^40^ (equations (7)–(9)).

7 $${Y}_{{\rm{n}}{\rm{o}}{\rm{v}}{\rm{e}}{\rm{l}},k,j}={a}_{k,j}\times {y}_{t,j}$$

8 $${y}_{t,j}={B}_{j}{{X}_{t,j}}^{{b}_{j}}$$

9 $${{\rm{L}}{\rm{R}}}_{j}=1-{2}^{{b}_{j}}$$

where *a*_*k*,*j*_ represents the consumption factor of novel component *j* by technology *k*, expressed in units of *j* per tonne of crude steel; *y*_*t*,*j*_ denotes the cost of component *j* at time *t* (in US$ per unit of *j*); *B*_*j*_ is the initial cost at the first unit capacity, *X*_*t*,*j*_ is the cumulative capacity of component *j* by time *t*, *b*_*j*_ is a parametric constant and LR_*j*_ is the learning rate for component *j*. Technical consumption factors (*a*_*k*,*j*_) of various decarbonization technologies were collected from previous studies^2,10^. Values of *B*_*j*_, LR_*j*_ and *X*_*t*,*j*_ for various novel components were obtained from historical databases and systematic literature reviews to ensure consistency. Supplementary Notes 3–5 describe the data sources (Supplementary Tables 2–4 and 8) and Supplementary Notes 9–10 outline the systematic review process (Supplementary Figs. 30–41 and Supplementary Tables 9–18). Regional variations in initial costs and cost reductions were carefully considered, provided relevant references were available.

By summing up the plant-specific cost of processing routes (*Y*_route_) with the regional-varied future cost of novel components (*Y*_novel_), we obtained the dynamic costs of 20 promising decarbonization technologies for each steel plant, laying a solid data foundation for subsequent plant-level economical pathway exploration. Several factors may influence future cost estimates, including initial costs, learning rates and cumulative capacities of novel components (for example, CCS and H_2_), as well as price fluctuations of key raw materials and energy sources such as iron ore, scrap, coke and electricity (Supplementary Table 5). Sensitivity analyses of cost forecasts for all promising technologies with respect to 22 key factors were conducted (Supplementary Note 7).

### Scenario design

Of all the technology transition options, we propose a bottom-up method to optimize plant-level net-zero pathways for global iron and steel plants, aiming to achieve retrofitting at the lowest production cost. In brief, each steel plant is assumed to undergo retrofitting every 20 years, consistent with the average capital investment cycle of steel equipment^7^. The construction period is simplified in this study owing to limited data availability. On the basis of the carbon-neutrality target years pledged by different countries, we estimate that all existing iron and steel plants would need to retrofit one to three times between now and their respective target years.

Without policy intervention (the reference scenario), only a few plants are expected to voluntarily implement efficiency improvements motivated by potential cost savings, yielding limited emission reductions. To achieve carbon neutrality, we consider three policy scenarios that enforce the deployment of decarbonization technologies by restricting the available technology options with varying levels of stringency. In all policy scenarios, steel plants are required to adopt zero-carbon technologies by the target net-zero year, but flexibility remains regarding when deployment begins. Specifically, the three scenarios are defined as: (1) early deployment, adopting zero-carbon technologies at the first retrofit; (2) medium deployment, adopting low-carbon technologies during the first few retrofits and zero-carbon technologies at the final retrofit; and (3) late deployment, retaining current technologies until the final retrofit, at which zero-carbon technologies are adopted.

To maximize the economic benefit of each plant, we assume that every plant can choose the lowest-cost technology option available at each retrofitting time under different policy scenarios. Sectoral-level changes, such as the potential increase in regional steel demand and the growing availability of scrap, are set as exogenous constraints based on International Energy Agency projections^7^ (Supplementary Fig. 3). In regions such as India, the Middle East, Africa and other Asian countries, where the establishment of new plants is needed to meet growing demand, the technology costs of new plants are assumed to be the national capacity-weighted average values. In regions with increasing scrap supply, whether a steel plant transitions to Scrap–EAF depends on whether Scrap–EAF is more cost-effective than other decarbonization options in that region. By aggregating all plant-level choices, we derive the least-cost global net-zero transition pathway. We also calculate the total CO_2_ abatement potential and mitigation costs associated with this optimized pathway. The uncertainties in the optimal pathways, abatement potential and costs, arising from factors including technology cost forecasts, retrofitting cycles and energy transitions, are discussed in Supplementary Note 8 (Supplementary Table 6).

### Reporting summary

Further information on research design is available in the Nature Portfolio Reporting Summary linked to this article.

## Online content

Any methods, additional references, Nature Portfolio reporting summaries, source data, extended data, supplementary information, acknowledgements, peer review information; details of author contributions and competing interests; and statements of data and code availability are available at 10.1038/s41586-025-09658-9.

## Supplementary information

Supplementary Information

Reporting Summary

## Figures and Tables

**Extended Data Table 1:**
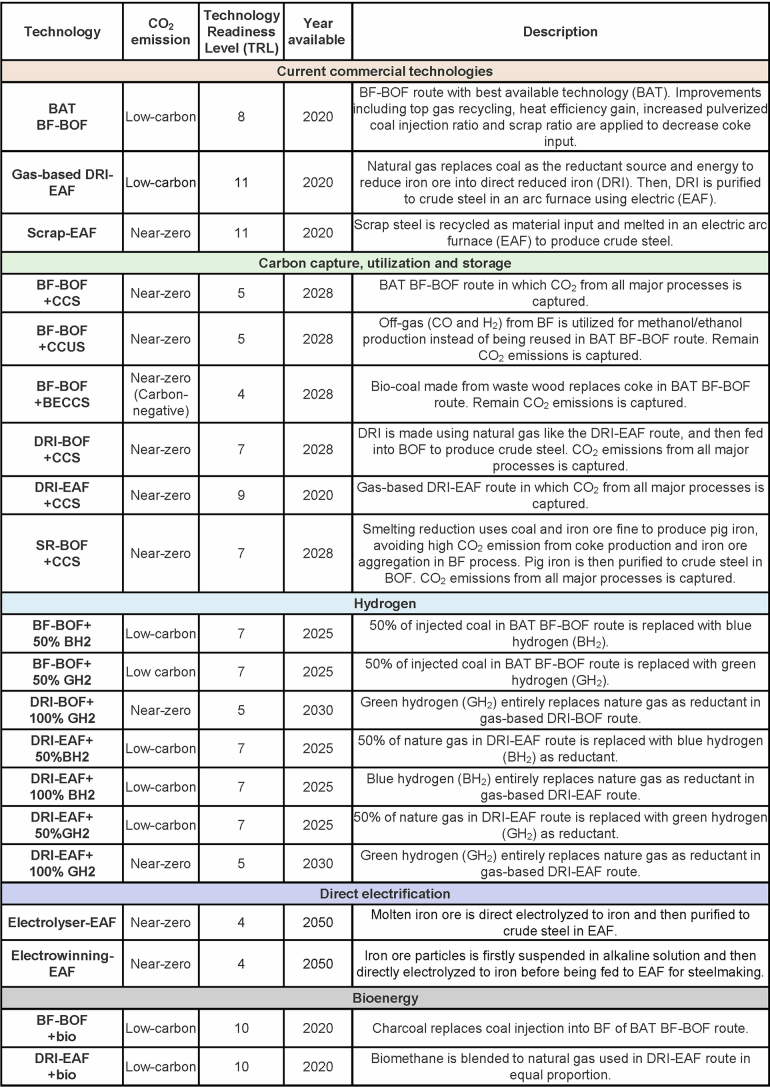
Promising low-carbon and near-zero emission technologies in the iron and steel sector

## Data Availability

The dataset of cost-effective decarbonization pathways for global iron and steel plants is available via Zenodo at 10.5281/zenodo.8214604 (ref. ^54^). It includes geographic locations and current technologies of iron and steel plants worldwide, along with plant-level data on cost-effective decarbonization technologies, emission reductions and abatement costs from 2020 to 2050 under three different carbon-neutrality strategies. In addition, all references used for the systematic reviews on CCS and H_2_ costs and learning rates (Supplementary Notes 9 and 10) are provided. Data for the future production of iron and steel by country are available on the website of the International Environmental Agency ETP Clean Energy Technology Guide: https://www.iea.org/data-and-statistics/data-tools/etp-clean-energy-technology-guide.

## Code availability

The code for the plant-level cost-effective technology pathway is available via Zenodo at 10.5281/zenodo.8214604 (ref. ^54^). It includes both R and Python scripts for implementing the plant-level net-zero pathway model, as well as procedures for conducting one-factor and multi-factor sensitivity analyses.
