# Supplementary material for: Technological pathways for cost-effective steel decarbonization
Source: Nature. 2025 Oct 29;647(8088):93–101. doi: 10.1038/s41586-025-09658-9 (PMC12589104; doi:10.1038/s41586-025-09658-9)
Supplement: Supplementary file 1 — Supplementary Information [file 41586_2025_9658_MOESM1_ESM.pdf]

nature portfolio

https://doi.org/10.1038/s41586-025-09658-9

Supplementary information

Technological pathways for cost-effective steel decarbonization

In the format provided by the authors and unedited

Technological Pathways for Cost-Effective Steel Decarbonization

Xinyi Wu[1,2], Jing Meng[1,2 *], Xi Liang[1], Laixiang Sun[3], D'Maris Coffman[1], Andreas Kontoleon[4], Dabo Guan[1,5]

[1] The Bartlett School of Sustainable Construction, University College London, London, WC1E 7HB, UK

[2] Centre for Sustainability Science and Technology, University College London, London, UK

[3] Department of Geographical Sciences, University of Maryland, College Park, MD 20742, USA

[4] Department of Land Economy, University of Cambridge, Cambridge CB3 9EP, UK

[5] Department of Earth System Sciences, Tsinghua University, Beijing, 100080, China

[*] Corresponding author: Jing Meng (jing.j.meng@ucl.ac.uk)

Supplementary Information

Part A - Supplementary data, results, and analyses

Note S1. Literature review and statement of novelty.

To achieve net-zero, it is crucial to support not only policymakers but also individual plants worldwide in identifying the most cost-effective, technically mature, and plant-compatible decarbonization solutions. However, each steel plant has its own unique production and cost patterns. Any "one-size-fits-all" approach that lumps different entities together may fall short in motivating individual climate actions and potentially rendering carbon neutrality targets unachievable. Hence, plant-specific, tailor-made decarbonization pathways that consider the unique characteristics of each plant are necessary for global effective mitigation.

Previous studies have made significant efforts to explore decarbonization roadmaps for the iron and steel sector. Most studies have focused on national or sectoral-level mitigation strategies, optimizing decarbonization pathways by minimizing total sectoral costs using top-down models or Integrated Assessment Models (IAMs)[1-3]. However, these studies overlooked the plant-level heterogeneity in processing routes, production costs, equipment ages, locations, and techno-economic feasibility of promising decarbonization technologies, which might lead to plant-varied optimal decarbonization technologies. Some recent research investigated plant-level phaseout or mitigation strategies for China's[4,5] or the global iron and steel sector[6-8] based on non-economic indicators like plant-varied ages, locations, and emission intensities. Yet, existing plant-level studies did not consider the techno-economic viability of different decarbonization technologies specific to each plant. Thus, their resulting pathways may not be the most economical, and therefore not attractive or instructive enough to guide the decarbonization of steel stakeholders, who often prioritize economic cost over emissions reductions.

In order to explore the plant-level cost-effective pathway, it is important to conduct techno-economic analysis and plant-specific cost forecasting for various decarbonization technologies. However, there have been limited studies on future cost projections for steelmaking technologies, largely because many of these technologies are not yet commercialized. Among the dozens of works we reviewed, only three existing research performed cost predictions on steelmaking decarbonization options, while others provided static cost estimates of hypothetical plants or demonstrated projects with specific processes (Table S1). In detail, Leeson et al. (2017) projected the cost of CCS on steel plants using the learning curve method, but they did not consider the regional cost variations and different steelmaking processing routes[9]. Devlin et al. (2023) estimated the cost of DRI-EAF with green hydrogen through a machine learning approach, considering cost variations of both hydrogen and the DRI-EAF processing route across 68 countries[10]. The Emission Transition Committee [11] predicted the cost of 11 technologies based on expert elicitation, with cost variations in processing routes and regional differences[11]. Yet, none of these studies were based on actual plant data, which limits their practical applicability to real-world individual plants globally. As a

result, they are not suitable for identifying cost-effective technology choices at the plant level.

To fill the research gaps, this study for the first-time forecasts the plant-specific cost for 20 promising steelmaking decarbonization technologies, and tailors the most cost-effective decarbonization pathways for each of the iron and steel plants globally. This marks the first implementation of comprehensive full-process research at the global plant-level, integrating plant-varied current production and cost patterns, techno-economic evaluation of various decarbonization technologies, plant-specific net-zero pathways optimization, as well as abatement potential and abatement cost of each plant. As a complement to existing roadmaps, this study offers stakeholders more practical and specific guidance on decarbonization and provides the government with a more accurate basis for design mitigation strategies.

Table S1. Characteristics of recent research on decarbonization roadmaps and technology cost forecasts for the steel sector.

1) Literature on iron and steel roadmaps

Author	Data scale	Pathway scale	Model	Cost optimization	Pathway identification
Brinckerhoff (2015)	National level, UK	National	DACC & BIS	Yes	Total cost minimization
Yang et al. (2022)	National level, China	National	MAPLE-China (IAMs model)	Yes	Total cost minimization
Speizer et al. (2023)	Sectoral level, Global	Sectoral	GCAM (IAMs model)	Yes	Total cost minimization
Ren et al. (2021)	National level, China	National	MEID (IAMs model)	Yes	Total cost minimization
Li et al. (2022)	Plant-level, China	Aggregated to National	AIM (IAMs model)	Yes	Total cost minimization
Xu et al. (2023)	Plant-level, Global	Plant-level	None	No	Carbon intensity
Lei et al. (2023)	Plant-level, Global	Plant-level	None	No	Age and location

2) Literature on cost estimates of steelmaking technologies

Author	Technology type	Number of technologies	Include steelmaking route	Time variation	Regional variation	Method	Applied for pathway
ETC (2021)	CCS, BECCS, H2, Direct electrification	11	Yes	Yes	Global range	Expert elicitation	Yes
Devlin et al. (2023)	H2-based DRI-EAF	1	Yes	Yes	68 countries	Machine learning	No
Lesson et al. (2017)	CCS for steel plant	1	No	Yes	No	Hypothetical model	No
IEA (2020)	CCS, H2	5	Yes	No	Global range	Hypothetical model	Yes
Yang et al. (2022)	CCUS, H2	28	Yes	No	China	Hypothetical model	Yes
Fan et al. (2021)	CCS, H2, Biomass, Zero-carbon electricity	15	Yes	No	USA	Hypothetical model	No
Ren et al. (2021)	Enery efficiency improvements	Multiple	No	No	No	Review	No
Vogl et al. (2018)	H2-based DRI-EAF	1	Yes	No	No	Hypothetical model	No
Conde et al. (2022)	H2-based DRI-EAF	1	Yes	No	No	Project data (H2FUTURE project)	No
Fajardy et al. (2021)	BECCS for steel plant	1	No	No	No	Hypothetical model	No
Yang et al. (2021)	BECCS for steel plant	1	No	No	No	Review	No
Elsheikh et al. (2022)	H2-based DRI-EAF	1	Yes	No	No	Hypothetical model	No
Superchi et al. (2023)	H2-based DRI-EAF	1	No	No	No	Hypothetical model	No

Note S2. Supplementary data.

Existing technology patterns. In general, 64% of global steel production and 90% of sectoral CO_2 emissions come from the 361 BF-BOF plants, followed by Scrap-EAF plants (n=1272, 28% capacity and 4% emissions), DRI-EAF plants (n=83, 6% capacity and 3% emissions), and BF-OHF plants (n=36, 2% capacity with 3% emissions) which are being phased out in most regions due to the high emission intensity (Fig. 1-2). Ironmaking via blast furnace (BF) or direct reduced iron (DRI) is an essential process for most steel production, making up over 70% of the corresponding steelmaking emissions, and is used in 215 iron plants worldwide (Fig. 1 a and d).

CO_2 emission patterns. Globally, average CO_2 emission intensities of different processing routes vary significantly: BF-OHF (2.8 tCO_2/ton), BF-BOF (2.0 tCO_2/ton), DRI-EAF (1.0 tCO_2/ton), Scrap-EAF (0.1 tCO_2/ton). The variations in carbon intensity, both across and within processing routes, are primarily driven by differences in the type and quantity of energy consumed. For instance, among BF-BOF plants, those predominantly relying on coke as the main energy source and reductant have a higher carbon emission intensity than those using coal, and significantly higher than those utilizing charcoal as a substitute. Similarly, DRI-EAF plants using coal as a feedstock generally have higher CO_2 emissions compared to those powered by natural gas (Extended Data Fig. 3).

Regional average CO_2 emission intensity largely depends on each region's technology portfolio and energy sources. Regions with a high share of the emission-intensive BF-BOF process and substantial coal consumption tend to have higher carbon intensities. Consequently, China and India have the highest averages exceeding 1.7 tCO_2/ton, followed by Japan and South Korea, Other Europe and CIS, Latin America, EU 27 and UK with intensities between 1.2-1.4 tCO_2/ton. In contrast, North America and the Middle East, predominantly using electricity-based Scrap-EAF and gas-based DRI-EAF, show the lowest regional intensities at 0.7-0.8 tCO_2/ton (Extended Data Fig. 3b).

Furthermore, the relationship between regional production costs and CO_2 emission intensity is complex. China, in its effort to ensure a stable supply of high-quality steel at scale, incurs both high production costs and high emission intensities. Countries such as those in the EU have achieved lower emission intensities but at higher production costs by implementing advanced low-emission technologies like Scrap-EAF, adopting high energy efficiency measures, and enforcing stringent environmental policies. Conversely, India maintains lower production costs by relying on cheap but highly polluting coal, as well as emissions-intensive iron ore mining and transportation, which results in high carbon intensities.

Decarbonization under the medium policy scenario. In the medium deployment scenario, Chinese plants have the lowest average abatement cost, with 199 BF-BOF plants representing 85% of national capacity (i.e., 612 Mt crude steel), that can abate emissions at -\$2/tCO2 to \$8/tCO2 by retrofitting to BAT BF-BOF in the short term and

to Scrap-EAF or SR-BOF+CCS in the long term (Extended Data Fig. 6). Furthermore, 114 BF-BOF plants (225 Mt capacity) in the EU, Japan, Korea, Latin America, Other Asia and Pacific, and Other Europe and CIS, can be decarbonized at a negative cost of -\$44/tCO2 due to enhanced efficiency of BAT BF-BOF. Regions dominated by Scrap-EAF plants, such as EU27 & UK, and Japan and Korea, will see over 90% and 63% of national capacity remain unchanged without production cost increase, respectively. In contrast, it will cost as much as \$15-65/tCO2 for DRI-EAF plants in EU27 & UK, India, Africa, and Latin America to decarbonize with hydrogen-based technologies.

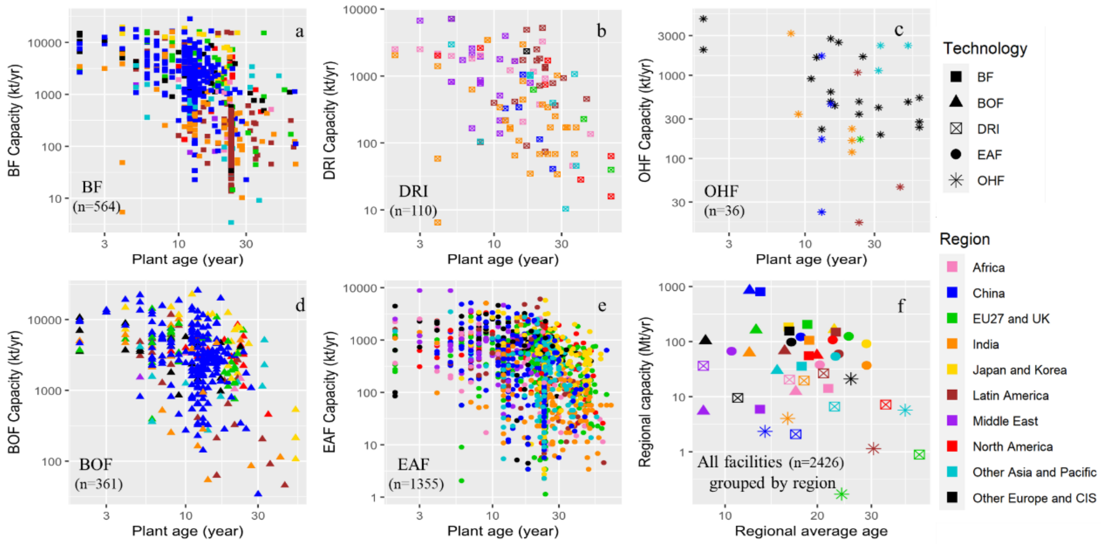

Fig. S1 Global distribution, technology, capacity and age of ironmaking and steelmaking facility units. This plot presents the characteristics of 2,464 facility units within 1,967 plants worldwide, excluding plants solely engaged in downstream steel processing. Colors indicate geographical locations, and shapes denote technology types: a) blast furnace (BF; square), b) direct reduction iron (DRI; square), c) open-hearth furnace (OHF; star), d) basic oxygen furnace (BOF; triangle), and e) electric arc furnace (EAF; circle). Capacity represents the annual iron output for BF and DRI, or crude steel output for BOF, EAF, and OHF. Age is measured from the commissioning year to 2021. (f) displays the total capacity and average age of each technology type across regions.

Note: Fig. S1 displays data at the facility unit level, whereas the main text (e.g., Fig. 1) aggregates data at the plant level and by processing route to better compare production costs and decarbonization pathways across process routes. For example, the BF category in Fig. S1 (a) includes all BF equipment from Fig. 1 (a-c), regardless of their integration with steelmaking facilities. Similarly, the EAF category in Fig. S1 (e) encompasses all EAF units from Fig. 1 (e-f). Therefore, the number of data points and their representation differ between Fig. S1 and Fig. 1 in the main text.

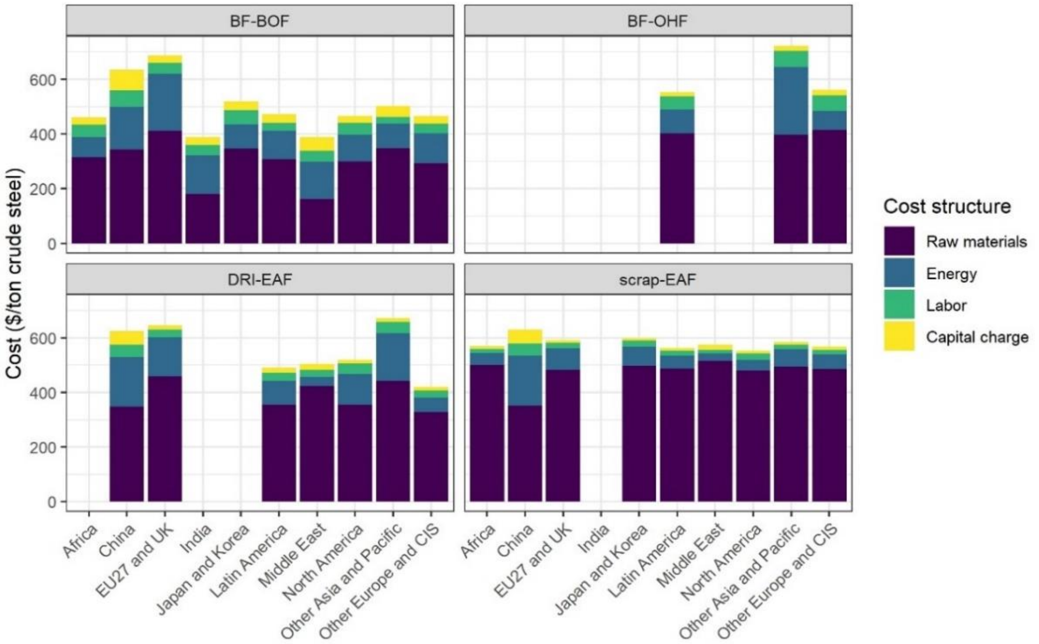

Fig. S2 The cost structure of different steelmaking technologies across regions. a)-d) presents the regional production cost of BF-BOF, DRI-EAF, BF-OHF and Scrap-EAF steel. The texts on the X-axis are the region abbreviations of Africa, European Union 27 and UK (EU), India, Japan and Korea (J&K), Latin America (LA), Middle East (ME), United States (USA), Other Asia and Pacific (A&P), Other Europe and CIS (CIS).

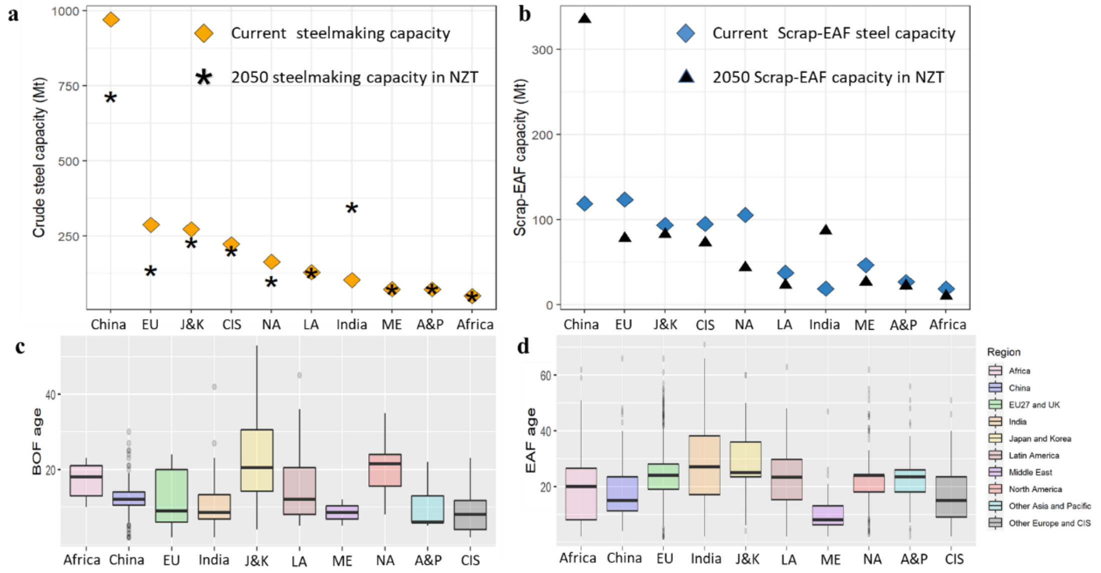

Fig. S3 Key assumptions and parameters of scenario design. A) Gap between current regional steelmaking capacity and their expected capacity by year 2050. B) Scrap-EAF steel capacity changes till 2050 caused by scrap supply increases in China and India, and steel production cut in other regions. The projected capacity of 2050 in both a) and b) were obtained from IEA Sustainable Development Scenario (SDS), while the current capacity was calculated from our plant-level database. C) and d) showed the regional age distribution of BOF and EAF, respectively.

Note S3. Cost estimation for processing routes.

Identify the primary factors of current processing routes. According to our plant-level cost databases, the production cost of each processing route in each steel plant is comprehensively detailed, encompassing 37 specific cost expense items, thereby providing an in-depth breakdown of the overall expenditure. We classify all the items into four categories and calculate their proportion in the total cost: raw materials (59-83%), energy (10-23%), labor and management (5-14%), and levelized capital investment (2-8%). It is shown that raw materials and energy are the major cost contributors (Fig. S5). Furthermore, iron ore, scrap, coke or coking coal, and electricity dominate the cost of raw materials and energy, accounting for 9-74% of the total production cost, and thus are identified as the primary factors influencing the expense of current processing routes (see details in Note S6). The production cost of an existing steel plant ($\boldsymbol{Y_{plant}}$) can be denoted as the sum of costs of iron ore, scrap, coking coal, electricity, and the others (Eq. S1).

$$Y_{plant} = Y_{raw\ material,plant} + Y_{energy,plant} + Y_{labor,plant} + Y_{capital,plant} =$$

$$Y_{iron\ ore,plant} + Y_{scrap,plant} + Y_{coking\ coal,plant} + Y_{electricity,plant} + Y_{other,plant} \quad \text{(S1)}$$

Estimate plant-varied cost of new processing routes. Promising decarbonization technologies involve 5 main types of processing routes: not only existing BF-BOF, DRI-EAF, and scrap-EAF, but also innovative DRI-BOF and SR-BOF (Extended Data Table 1). The cost of a new processing route for a specific plant ($Y_{new\ route}$) can be estimated based on the plant current cost patterns (Y_{plant}) according to Eq. S2-S5.

$$Y_{new\ route} = Y_{raw\ material,new} + Y_{energy,new} + Y_{labor,new} + Y_{capital,new} \quad \text{(S2)}$$

$$Y_{raw\ material,new} = \sum_{i=1}^{n} Y_{i,plant} \times IF_{i,new}/IF_{i,plant} \quad \text{(S3)}$$

$$Y_{energy,new} = \sum_{j=1}^{n} Y_{j,plant} \times IF_{j,new}/IF_{j,plant} \quad \text{(S4)}$$

$$Y_{labor,new} + Y_{capital,new} = Y_{labor,plant} + Y_{capital,plant} \quad \text{(S5)}$$

where,

i and j represent the items categorized as raw materials and energy, respectively;

$Y_{i,plant}$ and $Y_{j,plant}$ are the plant-specific current cost of raw material i and energy j;

$IF_{i,new}$ and $IF_{j,new}$ denote the input factors for a new processing route, while $IF_{i,plant}$ and $IF_{j,plant}$ are those for plant current processing route.

For each processing route, the input factors (*IF*) of key materials and energy (e.g., iron ore, scrap, coking coal, thermal coal, electricity, biomass) are meticulously collected from previous studies[11-20]. Yet, input factors for other less significant items of raw materials and energy, as well as the cost of labor and capital, are assumed the same as those of the plant's current processing route. Additionally, the future cost of processing

route may be significantly influenced by the price fluctuations of key raw materials and energy, as shown in Eq. S6-S7.

$$Y_{raw\ material,new,t} = \sum_{i=1}^{n} Y_{i,new,t} \times P_{i,t}/P_{i,t=o} \quad (S6)$$

$$Y_{energy,new,t} = \sum_{j=1}^{n} Y_{j,new,t} \times P_{j,t}/P_{j,t=o} \quad (S7)$$

where, P_t is the price of key item i or j in year t, $P_{t=o}$ is the initial price of key items in base year 2021 of the plant-level cost database.

In comparison to technology cost and learning rates, long-term price forecasts are subject to greater uncertainty and lack of authoritative references. Moreover, unlike the costs of novel components, which vary significantly across different steelmaking technologies, price fluctuations have a limited effect on the relative cost differences between technologies, particularly those sharing the same processing route, and on plants' choices of the least-cost technologies. Therefore, our future cost projections for each processing route in the main text assume constant prices. Yet, to ensure the comprehensiveness and robustness of the results, we explore the impact of price changes of key materials and energy, like iron ore, scrap, coke, electricity, on the cost of each technology, plants' least-cost technology choices, and carbon abatement pathways, abatement potentials, and abatement costs in our uncertainty analysis (see details in Note S7).

Note S4. Cost projection for CCS, CCUS and BECCS in steel plants.

There are two main model-based cost forecasting methods: Wright's law, which assumes the evolution of costs as a function of cumulative production or deployment, and Moore's law, which assumes the evolution of costs as a function of time[21]. Compared to projections using Moore's law[9,22,23], Wright’s law is particularly suitable for novel technologies like CCS, which develop unevenly, exhibit significant stop-and-go patterns, and have substantial spillover effects across sectors[24-27]. Therefore, in this study, we applies Wright’s law to forecast the dynamic cost of novel decarbonization technologies, emphasizing the cost reductions driving by cumulative deployment and spillover effects across regions and sectors. According to Eq. 8-9 in the main text, the parameters needed for Wright’s law cost projection are initial cost (B), learning rate (LR), and cumulative capacity (X).

Initial cost. We conducted a systematic review on CCS capture cost in steel plants following the PRISMA guidelines, identifying 16 key studies out of 1088 initially screened. From these studies, 30 cost estimates spanning 2011–2021 were extracted and converted to 2021 dollars using the Chemical Engineering Plant Cost Index (CEPCI), following Leeson et al[9]. However, significant variability in these estimates arises due to differences in methods, technical assumptions, economic factors, and geographic scopes, raising concerns about comparability (see Note S9.1 in Supplementary Information Part B). For instance, some studies modeled costs for hypothetical facilities in Europe[28-30], the United States[22,31-33], or unspecified regions[34-37], while others reviewed previous studies and reported average values[9,23,24,38] (Fig. S4). The Global CCS Institute provided cost estimates for commercial-scale CCS projects in 14 global regions, combining regional input price adjustments with a unified methodological framework to ensure internally consistent and comparable results[39]. To align with the global-scale, plant-level scope of this study and to reflect regional variations, we adopted the regional CCS cost estimates reported by the Global CCS Institute[39] as baseline initial costs (B). For uncertainty and sensitivity analyses, we applied a ±2 standard deviation range based on the distribution of all literature values, as detailed in Notes S7–S8.

Learning rate. A systematic review was conducted to assess projected cost reductions and learning rates for CCS in the steel sector and other industries (see Note S9.2 in Supplementary Information Part B). This review was not limited to the steel sector, as the technological similarity of CCS deployment across industries suggests comparable cost reduction rates[24]. Studies on the steel sector often referenced CCS cost trajectories from power plants[9]. Table S2 provides example studies illustrating the research landscape. Some studies estimated future CCS costs for steel plants using Wright's law[9,40,41], while others reviewed CCS learning rates for power plants without cost forecasts[24,42], or applied Moore's law to project cost reductions over time[9,22,23]. A detailed summary of the literature screening and characteristics is presented in Fig. S33 and Table S13 in Note S9.2. To align with this study's methodology, we identified 9

studies from the 1883 initially screened that provided original learning rate estimates based on Wright's law. An average learning rate (LR) of 7% was adopted for baseline analyses, with a ±6% range (corresponding to two standard deviations) applied in uncertainty and sensitivity analyses.

Cumulative capacity. To apply Wright's Law, it is necessary to obtain the cumulative installed capacity of each novel component from 2020 to 2050. Upon reviewing the literature, we found diverse assumptions about cumulative capacity: some papers predicted linear increases[41], others anticipated S-curve adoption[9,40]; the data forms varied from national sector capacities to technology penetration rates, and even the number of plants (Table S2). None of these assumptions suited our global scope and plant-level granularity.

Therefore, we applied a sigmoid function (S-shaped curve) to predict technology adoption of each novel component, following previous studies on energy technologies[43,44]. The equation is shown below (Eq. S1).

$$A_t = \frac{A_s}{1+\frac{A_s - A_0}{A_0} * e^{-kt}} \qquad \text{(Eq. S1)}$$

where A_t is the capture capacity of CCS in year t, A_0 is the initial capacity, A_s is the maximum capacity that will be reached long-term, and k is the growth rate. In this study, A_0 is set at 0.8 Mt CO_2, based on the capacity of the Abu Dhabi CCS project at Emirates Steel Industries, the world's first CCS project on steel plants, operational since 2016. The values of A_s and k are determined by fitting the announced CCS project capacity of the steel industry up to 2023[45], along with the IEA's projected CCS capacity for 2050 (A_{2050}=1.0 Gt CO_2)[20], to the S-shaped curve (Eq. S1). This fitting results in a growth rate (k) of 23% and a maximum capacity (A_s) of 2.3 Gt CO_2. The projected capacities for key years are shown in Table S4. The cumulative capacity [24] required for Wright's law corresponds to A_t in the derived S-shaped curve[24]. Based on the regionally varied initial cost, learning rate, and S-curve of CCS deployment obtained above, we can project the future cost of CCS as a function of cumulative capacity (as per Eq. 8-9 in the main text) and relate the forecasted cost to time using the S-curve.

For CCUS and BECCS technologies in steel plants, we use CCS as a historical analogy, assuming the same learning rate of 7%, following the methodologies of Sievert et al. (2024)[46] and Edwards et al. (2024)[44]. The initial capture cost for CCUS is \$73/tCO_2 (ranging from \$64-82/tCO_2) and for BECCS is \$156/tCO_2 (ranging from \$126-192/tCO_2), based on average values from previous research[11,12,47-49]. Furthermore, the S-curve for CCUS deployment is assumed to be the same as for CCS[45], while the future capacity of BECCS is projected by fitting the annual capture capacity of existing and announced BECCS projects[50] to the sigmoid function in Eq. S1. Please refer to Table S4 for detailed cumulative capacity projections.

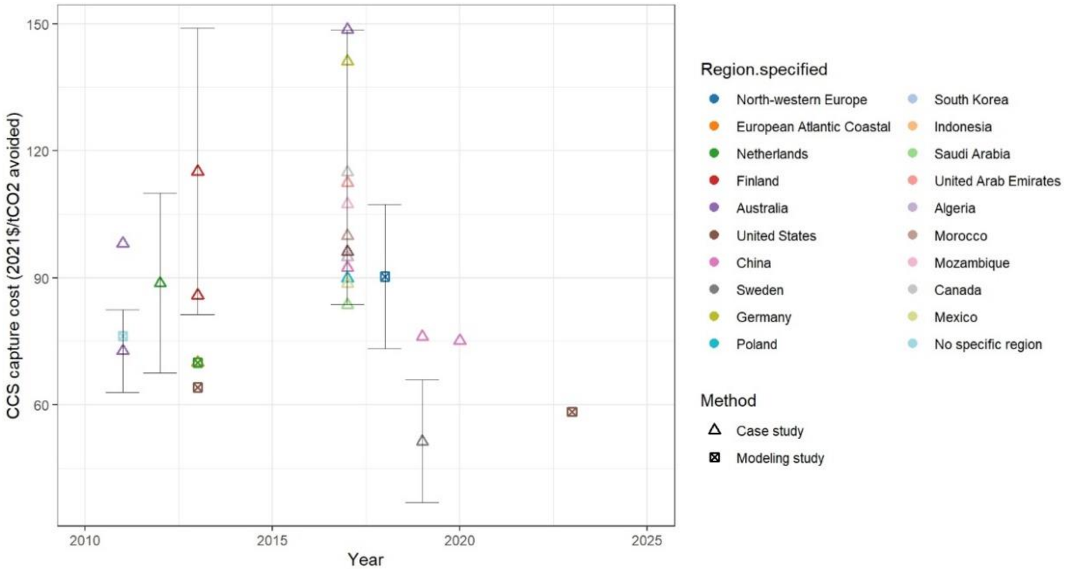

Fig. S4 Literature estimates of CCS costs in iron and steel sector. The points represent CCS capture costs in steel plants from 16 key studies identified through a systematic review. Point shapes indicate the study methods: triangles for case studies and squares for modeling studies. Point colors denote the regions of cost estimates: light blue for unspecified regions, brown for the United States, pink for China, and others. Cost values from different years were standardized to 2021 dollars using the Chemical Engineering Plant Cost (CEPC) index.

Table S2. Representative recent studies reporting CCS cost reductions in the steel and power sectors.

**Sectors**	**Author**	**Cost projection method**	**Learning rate/ Assumed cost changes**	**Scaling up trajectory**
Iron & steel	Ding et al. (2020)[40]	Wright's law	8% [b]	S curve with growth rate of 22% [b]
Iron & steel	Lee et al. (2022)[41]	Wright's law	10% [a]	Linearly increase by 3% per year [b]
Iron & steel	Leeson et al. (2017)[9]	Wright's law	3.50% [a]	S curve with growth rate of 30% [b]
Fossil power	Bui et al. (2018)[24]	Wright's law (Lit. Rev.)	5% (ranging 1.1-9.9%) [a]	Not involved
Fossil power	Malhotra and Schmidt (2020)[42]	Wright's law (Lit. Rev.)	7% (ranging 2-15%) [a]	Not involved
Iron & steel	Leeson et al. (2017)[9]	Moore's law	Cost reduces by 25%, 20%, 15%, 10%... every 5 years [b]	--
Fossil power	GCCSI (2021)[22]	Moore's law	Cost reduces by 50% from 2010-2025 [b]	--
All sectors	Grant et al. (2021)[23]	Moore's law	Cost reduces by 40-70% from 2020-2100 [b]	--

Note: This table highlights a few representative CCS cost studies. See Note S9.1 in SI Part B for the full set of reviewed literature.

[a] Derived from a literature review (abbreviated as Lit. Rev.) or individual literature sources.

[b] Study assumption without a reference provided.

[c] Data collected from actual projects.

Note S5. Cost projection for green H_2, blue H_2, and electrolyser.

The levelized cost of green hydrogen production (LCOH, \$/kgH_2) is primarily dominated by the capital cost of the electrolyser (\$/kW) and the cost of electricity (\$/MWh)[51,52]. Previous studies on electrolytic hydrogen cost projection can be categorized into three types: 1) those focusing on the cost reduction of renewable electricity (RE) while keeping electrolyser characteristics constant[31,53], 2) those analyzing the learning effect of electrolyser investment with a fixed or average electricity price[52,54-58], and 3) those considering the cost reductions of both electrolyser and renewable electricity[51,59]. However, most studies that incorporate dynamic costs for both electrolysers and renewable electricity either project only to 2030 rather than extending to 2050[51], or are confined to Europe instead of having a global scope[59], which is insufficient for this study on global steel plants over 2020-2050 (see Table S3).

Therefore, we project the future levelized cost of green hydrogen production [60] using component-based Wright's law (see Eq. S2-S6).

$$y_{H,t} = y_{RE,t} + y_{EL,t} + y_{other,t} \quad \text{(Eq. S2)}$$

$$y_{RE,t} = y_{RE,0} * \left(\frac{X_{RE,t}}{X_{RE,0}}\right)^{b_{RE}} = c_{RE} * y_{H,0} * \left(\frac{X_{RE,t}}{X_{RE,0}}\right)^{b_{RE}} \quad \text{(Eq. S3)}$$

$$y_{EL,t} = y_{EL,0} * (\frac{X_{EL,t}}{X_{EL,0}})^{b_{EL}} = c_{EL} * y_{H,0} * \left(\frac{X_{EL,t}}{X_{EL,0}}\right)^{b_{EL}} \quad \text{(Eq. S4)}$$

$$y_{other,t} = y_{other,0} * \left(\frac{X_{H,t}}{X_{H,0}}\right)^{b_{other}} = c_{other} * y_{H,0} * \left(\frac{X_{H,t}}{X_{H,0}}\right)^{b_{other}} \quad \text{(Eq. S5)}$$

$$LR_j = 1 - 2^{b_j} \quad \text{(Eq. S6)}$$

where, $y_{j,t}$ and $X_{j,t}$ represent the cost and cumulative capacity/production of component j in year t, respectively; The subscripts H, RE, EL, and $other$ indicate green hydrogen, renewable electricity, electrolyser, and other cost components, respectively. c_j denotes the proportion of the initial cost of component j in the initial cost of green hydrogen production ($y_{H,0}$); b_j is a parametric constant and LR_j is the learning rate for component j.

We assumed the cost shares of renewable electricity (c_{RE}), electrolyser (c_{EL}), and other components (c_{other}) to be 50% (30-60%), 40% (30-60%), and 10%, respectively, according to cost breakdowns in previous studies[51,52]. The learning rates of renewable electricity (LR_{RE}) and electrolyser (LR_{EL}) are assumed as 20% (10-24%) and 18% (8-28%), respectively, while the cost of other components is assumed unchanged (LR_{other} < 5%)[54-56,58,59,61,62]. Numbers in parentheses indicate literature value intervals. See Table S3 for representative recent studies and Note S10.2 in the Supplementary Information Part C for the detailed systematic review.

The cumulative capacity/production of renewable electricity ($X_{RE,t}$), electrolyser ($X_{EL,t}$), and green hydrogen ($X_{H,t}$) are projected using the S-shaped curve of technology

adoption (Eq. S1). The parameters required for S-shaped curves, including growth rate (k), initial capacity (A_{base}), and maximum capacity (A_{sat}), are derived by fitting the historical data, announced projects, and the IEA's projected demands for 2030 and 2050, following the method in previous studies[56,61,63]. Specifically, the cumulative installed capacity of electrolysers (GW) and the cumulative production of green hydrogen (Mt H_2) are determined from announced projects spanning 2000-2040 in the IEA Hydrogen Projects Database[64] and predicted demand through 2050 in the IEA NZE scenario[65]. The data for renewable electricity encompass total electricity generation from solar PV and wind for the years 2000-2022[63], with projections for 2030 and 2050 in the IEA NZE scenario[65]. The resulted growth rates for renewable electricity (k_{RE}), electrolyser (k_{EL}), and green hydrogen (k_H) are 20%, 32% and 35%, respectively. Please see Table S4 for detailed cumulative capacity projections.

To determine initial costs for green hydrogen production ($y_{H,0}$), we conducted a systematic review of 6,065 publications following the PRISMA framework. Among the screened studies, PwC (2021)[53] was identified as the most suitable source, as it provided comparable cost estimates across a wide range of regions based on consistent methodologies and assumptions—unlike studies that apply heterogeneous assumptions, focus on specific regions, or report only future projections. While the baseline initial cost was derived from PwC (2021)[53], the full set of eligible studies was used to define the uncertainty range for sensitivity analysis, applying the mean ± two standard deviations across reported values. See Note S10.1 in Supplementary Information Part C for details. Regional variations in hydrogen cost projections are generated based on the differences in initial costs across regions. Consequently, we predict the future green hydrogen cost of \$2.0-7.5/kgH_2 in 2030 and \$0.9-3.5/kgH_2 in 2050. The resulted learning rate of green hydrogen (LR_H) over 2020-2050 is about 9.5%. Additionally, when adjusting the learning rates (LR_j) and cost shares (c_j) of renewable electricity, electrolysers and other components within the range of literature values, the derived learning rate for green hydrogen (LR_H) ranges from 6% to 13%. This range will be used for sensitivity tests in Notes S6-S8.

For electrolyser, we assume an initial cost of \$1.0/W in 2020, which is the average of reported ALK and PEM costs in the literature[58] (Table S3). An average learning rate of 18% from the literature was used for the baseline analysis, with a ±12% range (i.e., two standard deviations of 6%) applied as the uncertainty interval in the sensitivity analysis (see Note S10.2 in SI Part B for details). For blue hydrogen, we used the IEA's (2023)[51] regional costs as initial values (see Table S3 below) and applied the same 7% learning rate for CCS as detailed in Note S4. The cumulative capacities of electrolyser and blue hydrogen are projected by fitting an S-curve to the respective announced projects[64] and expected capacities for 2030 and 2050 in the IEA NZE scenario[65].

Table S3. Reported cost reduction of hydrogen production ($\$/kgH_2$).

Data source	Technology type	Region	Year 2020	Year 2030	Year 2050	Driver of cost change[a]	Installed capacity
**Green hydrogen**							
PwC (2021)[53]	RE-based hydrogen	20 countries	4.4-9.0	2.6-5.6	1.4-3.5	Assumed RE cost reduction	--
IRENA (2020)[54]	RE-based hydrogen	7 countries	--	1.1-2.5	0.7-1.7	LR=18% for electrolyser	5 TW in 2050
IRENA (2022)[62]	RE-based hydrogen	33 countries	--	--	0.9-3.5	Least RE cost	--
IEA (2020, 2023)[31,51]	RE-based hydrogen	Global (no particular country)	3.4-12[31,51]	1.6-8.2[31,51]	2.3[31,51]	LR=18% for electrolyser, RE cost reduction in NZE scenario	Historical capacity[64], and 590 GW in 2030[65]
Glenk et al. (2023)[58]	Electrolytic hydrogen	United States	--	1.6-1.9	--	LR=16% for electrolyser	115-248 GW in 2030
Zeyen et al. (2023)[59]	Electrolytic and RE-based hydrogen	Europe	--	1.5-2.3	1.4-1.7	LR=16% for electrolyser, LR=10-24% for RE cost	Endogenous in the energy system model
Yang et al. (2023)[66]	Electrolytic hydrogen	China	--	2.4-5.9	2.3-2.9	Efficiency improvement	--
**Blue hydrogen**							
IEA (2020, 2023)[31,51]	Fossil fuel w/ CCS	Global	1.5-5.0	1.0-2.7	1.7-2.3	Energy price reduction	--
Fan et al. (2021)[12]	Fossil fuel w/ CCS	United States	1.93	--	--	--	--
**Electrolyser**							
Multiple reference[51,52,54-59,61]	ALK, PEM, SOC	Global	0.6-5.6 ($/W)	--	--	LR=18%[b] (8-28%) for electrolyser	Historical capacity[52,54,56,64]

Note: [a] LR represents the learning rate, RE stands for renewable electricity, and NZE denotes the IEA's Net Zero Emissions by 2050 Scenario[65].

[b] Mean value of learning rates in Refs.[51,52,54-57,59,61]

Table S4. Projected cumulative capacity of novel components.

Cumulative capacity	Year 2020	Year 2030	Year 2050	Unit	Data source
CCS/CCUS	2	19	1000	Mt CO_2 in steel sector	IEA CCUS Project Database[45], IEA NZE scenario[65].
BECCS	2	60	1000	Mt CO_2 in all sectors	IEA Bioenergy with CCS[50], IEA NZE scenario[65], Journal papers[44,67].
Green hydrogen	27	51	327	Mt H_2 in all sectors	IEA Hydrogen Production Projects Database[51], Global Hydrogen Review 2023 [64], IEA NZE scenario[65].
Blue hydrogen	1	18	89	Mt H_2 in all sectors	
Electrolyser	2	247	3300	GW in all sectors	

Note: NZE represents the Net-Zero Emissions by 2050 Scenario.

Note S6. Costs and prices of key materials.

Primary factors of steelmaking cost. There are four main types of steelmaking technologies: BF-BOF, BF-OHF, DRI-EAF, and scrap-EAF. Fig. S5 illustrates the cost structure of these existing technologies worldwide, based on our plant-level global steel cost database. Generally, raw materials and energy costs are the major contributors to the total cost, constituting 59-83% and 10-23%, respectively, while the costs of labor, maintenance, and levelized capital investment amount to 7-23%. Within raw materials, iron ore and scrap dominate the cost, accounting for 44-62% and 25-87% of raw material expenses while comprising 29-39% and 16-74% of the total steelmaking cost, respectively. In terms of energy consumption, BF-BOF and BF-OHF plants primarily rely on coking coal, which accounts for 86% and 99% of their energy costs and 20% of the total steelmaking cost, respectively. Natural gas, predominantly used in DRI production, is economically viable primarily in regions with relatively low gas prices, such as the Middle East; it represents 9% of energy costs and 1% of the total steelmaking cost. DRI-EAF and Scrap-EAF plants are significant consumers of electricity; the cost of electricity consumption constitutes 58-89% of their energy cost but less than 10% of the total steelmaking cost. Thereby, the primary factors influencing the cost of steel production with current technologies are the prices of iron ore, scrap steel, and coking coal.

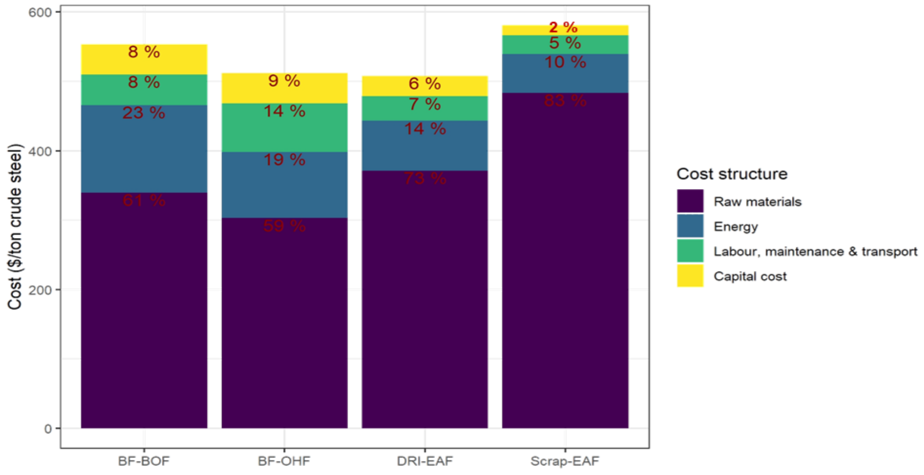

Fig. S5 Cost structure of existing steelmaking technologies worldwide. The cost value represents the capacity-weighted average for global steel plants. The cost of raw materials (depicted in purple) encompasses 28 items, including iron ore, direct reduced iron (DRI), pig iron, scrap, alloys, zinc, tin, electrodes, limestone, lime, inert gases, lubricants, acid or alkali cleaners, water, and net of by-product credits such as that from the sale of BOF slag. The cost of energy (shown in blue) covers coking coal, coke, charcoal, thermal energy, oxygen, and electricity. The cost of labor, maintenance, and transport (indicated in green) includes direct and administrative labor, maintenance supplies such as refractories, and the transportation of materials between stages of production. The capital cost (in yellow) accounts for interest, depreciation, property tax, and insurance on capital investments. Data is from our global steel cost database.

Prices of key materials and energy. As shown in Fig. S5, the prices of iron ore, coking coal, and scrap steel are the key factors of steelmaking cost of existing technologies.[1] Here, we collected the historical prices of these materials, and predicted their future prices based on historical trends.

Price of Iron ore. According to our global steel cost database, cost of iron ore accounts for 44-62% in plant cost of raw materials.[2] Since 2005, the global price of iron ore has experienced rapid increases due to high steel demand fueled by industrialization and urbanization. The first price spike occurred in 2008, followed by a notable decline in 2009 due to the severe economic downturn. Between 2012 and 2014, prices decreased again as steelmakers began acquiring their own iron ore and coking coal operations and sought new supply sources from countries with previously minor operations, aiming to reduce steelmaking costs.[3] However, post-2015, iron ore prices increased again, driven by surging steel demand and diminishing ore reserves. The prices reached a record high in 2021 Q1 again due to increased Chinese steel production during the COVID-19, but later fell after government calls for Chinese steel plants to cut production to address emissions and oversupply issues (Fig. S6).

At the plant level, iron ore prices for individual plants differ significantly, influenced by factors such as processing technology (e.g., Blast Furnace [BF], Direct Reduction [DR], Sintering [SF]), types of iron ore used (e.g., fines, pellets, lump), and geographical location, which affects access to iron ore supplies or imports (Fig. S7). Although there are notable differences in the prices of various iron ore types at any given time, their long-term trends tend to be similar. This similarity allows for the prediction of future prices of these iron ore types based on the same global trend.

Based on historical prices of global iron ore from 1960 to 2023, a linear fitting of the average price for each decade was conducted, with an R^2 value of 0.82 (Fig. S8). Since 2021, there has been a short-term decline in iron ore prices. However, in the long term, prices are expected to gradually increase due to the limited reserves of high-grade iron ore. As we are forecasting mid-to-long-term prices (2024-2050) and considering the approximately 20-year capital cycle of steel equipment, we use a more stable 10-year average forecast value for subsequent cost predictions, without factoring in the short-term impacts of economic and social environmental accidents on iron ore prices.

---

[1] https://www.steelonthenet.com/trends-3.html

[2] https://static1.squarespace.com/static/5877e86f9de4bb8bce72105c/t/61e790b43ddb95393be8dcc1/1642565821704/Global+Steel+Production+Costs+-+Jan2022.pdf

[3] https://www.steelonthenet.com/trends-3.html

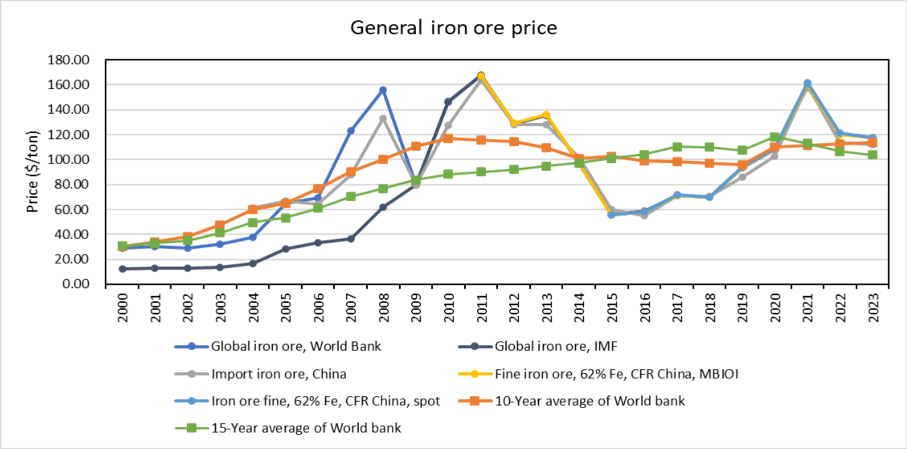

Fig. S6 Historical prices of global and typical varieties of iron ore. Historical Prices of global iron ore from the World Bank and the International Monetary Fund (IMF) are depicted in blue and dark blue, respectively. The annual average price, the Metal Bulletin Iron Ore Index (MBIOI), and the spot prices of iron ore imported by China are represented in grey, yellow, and teal, respectively. Given China's substantial share in the global steel market, these prices closely align with the global average price of iron ore and are often used as indicators to represent iron ore prices. Data collected from Wind and our global steel cost database.

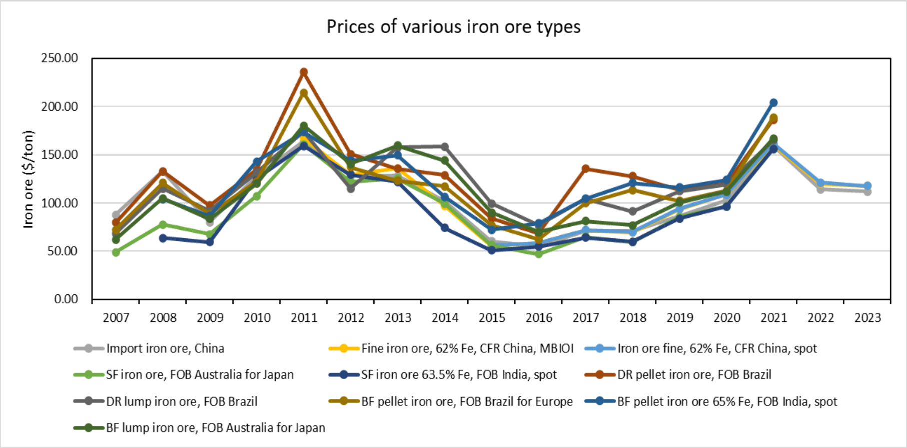

Fig. S7 Prices of various iron ore types. This plot presents the prices of different iron ore varieties (fine, pellet, lump), for various uses (SF, BF, DR), and from different exporting and importing countries (Australia, Brazil, China, India, Japan), including spot, Free on Board (FOB), and Cost and Freight (CFR) prices. It has been observed that the price fluctuations of various types of iron ore are consistent with the typical price indicators of iron ore in China (i.e., import, MBIOI, spot prices of China). This suggests that for predicting future prices of iron ore across various regions and types, it is practical to consistently reference the trends in global and Chinese iron ore prices, as illustrated in Fig. S2. Data collected from Wind and our global steel cost database.

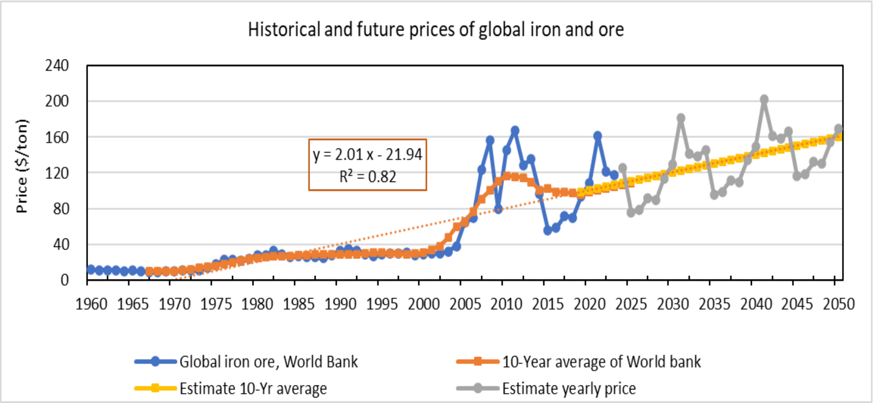

Fig. S8 Historical and projected prices of global iron ore. The blue and orange dots represent the historical prices of global iron ore from World Bank on an annual and decadal basis, respectively. The yellow dots indicate the predicted future 10-year average prices, derived from the linear trend of historical 10-year average values represented by the orange dots and depicted by the orange dashed line. The grey dots illustrate the annual prices retrodicted (inferred retrospectively) from the future 10-year average prices (indicated in yellow).

Price of scrap. The price of steel scrap is typically influenced by the prices of iron ore and coal, as these materials determine the production costs of companies that use them, setting a baseline for prices that scrap-based producers must compete with. Consequently, after 2005, scrap prices soared, significantly exceeding the costs of collection and processing. Here, we collected historical steel scrap prices from 2007 to 2023 and evaluated their long-term trend using ordinary least squares (OLS) regression. The results showed a p-value of 0.77 for the slope coefficient, indicating no significant time trend in scrap prices during this period. Accordingly, the historical average was used to project the future price, and the historical minimum and maximum values were adopted as the lower and upper bounds for sensitivity analysis (Fig. S9).

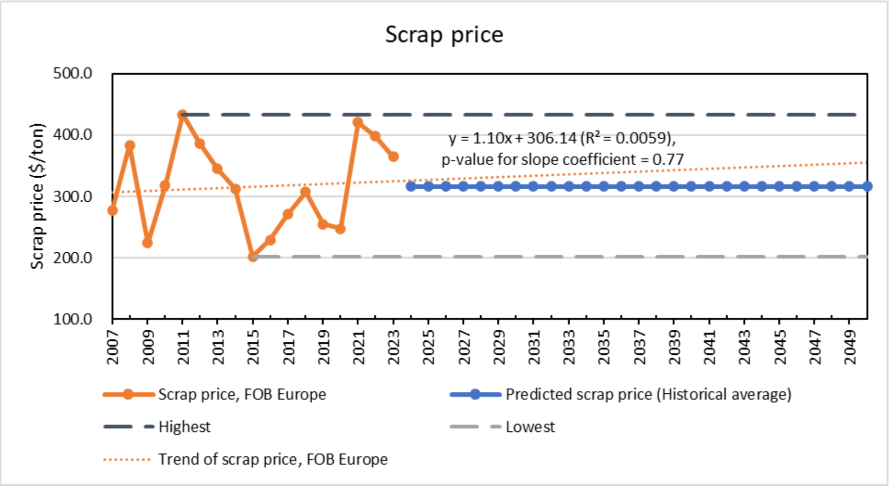

Fig. S9 Historical and projected prices of scrap. The historical Free On Board (FOB) price of steel scrap from Europe (2007–2023) is shown in orange. An ordinary least squares (OLS) regression revealed no significant time trend over this period (slope p-value = 0.77 > 0.10). The future FOB price (blue) and its upper (dark grey) and lower (light grey) bounds are linearly

extended based on the historical average, maximum, and minimum values. Data was collected from Wind, UN Comtrade, and our plant-level steel production cost database.

Price of coking coal. Similar to the price trend of iron ore, the price of coking coal, another main material, peaked in 2008, 2011, and 2022, and troughed in 2015 and 2020. Furthermore, due to the limited deposits of high-grade coking coal, future prices for good quality coal are likely to remain relatively high. Here, we predicted the future price of internationally traded coking coal based on the historical trend of the Free On Board (FOB) price from Australia, which is the primary source of globally imported coking coal (Fig. S10). It is feasible for long-term price trend forecasting when contingencies are disregarded. The credibility of our projection is further enhanced by the similarity between our projected growth trends in international coal prices and the industrial coal price trend forecasted by the U.S. Energy Information Administration (EIA). In addition, we forecasted the range of maximum and minimum future coking coal prices based on historical price extremes and average growth rate (i.e., the slope of the historical trend at 4.61).

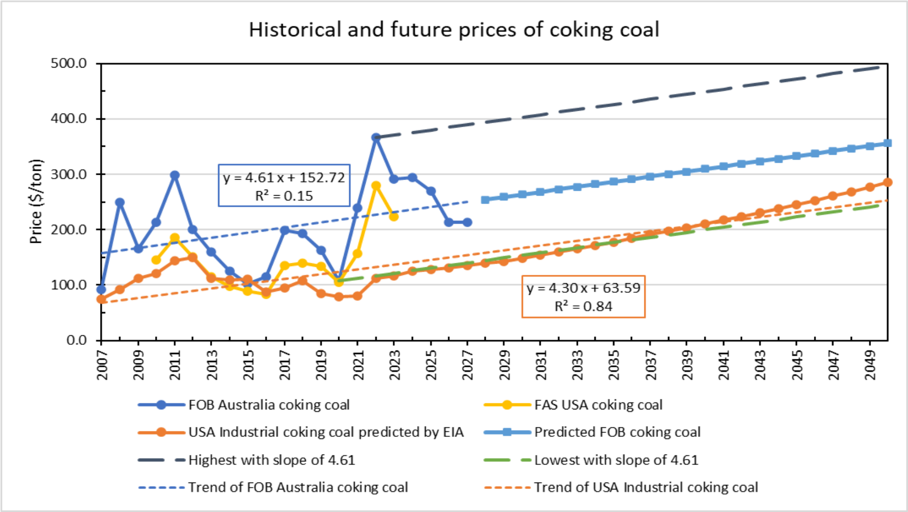

Fig. S10 Historical and projected prices of coking coal. Three key coking coal prices from 2007 to 2023 was gathered, including the Free On Board (FOB) price from Australia (dark blue), the Free Alongside Ship[60] price from the USA (yellow), and the domestic industrial coking coal price in the USA (orange), which represent typical costs of global coking coal consumption. The data were sourced from Refinitiv and our global steel cost database. The future price of industrial coking coal in the USA has been forecasted by the Energy Information Administration (EIA), while we have also projected future FOB prices (light blue) and their highest (dark grey) and lowest (green) possible ranges, following the historical trend's slope.

Note S7. Sensitivity tests of cost projections, and least-cost technology choice.

Comprehensive sensitivity analyses were conducted to examine the influence of learning rates, initial costs, and cumulative capacities of novel components, as well as the prices of key raw materials and energy, on the future cost projections of each technology and the least-cost technology choice.

Identification of the key factors. Twenty-two key factors that may significantly influence technology costs were identified, as shown in Table S5. These factors comprise four input prices, alongside the initial costs, learning rates, and cumulative capacities associated with each of the six novel components: CCS, BECCS, CCUS, green hydrogen, blue hydrogen, and electrolyser. Based on historical trends or literature, most parameters, including input prices, cumulative capacity and initial costs, are expected to vary within ±50% of their baseline values, while learning rates and the initial costs of green hydrogen may vary by more than ±70% (see details in Notes S4–S6 in SI Part A, S9 in SI Part B, and S10 in SI Part C). Uncertainty in these novel component-specific parameters will only impact technologies utilizing the corresponding components. In contrast, changes in the prices of iron ore, scrap, coke, and electricity will affect the costs of nearly all technologies.

Table S5. Key factors influencing cost projections.

Factor	Range	Influenced technology
Learning rate of CCS	-75%-75% [a]	4 technologies, including BF-BOF with CCS, etc.
Initial cost of CCS	-53%-39% [a]	
Learning rate of CCUS	-75%-75% [a]	BF-BOF with CCUS
Initial cost of CCUS	-39%-39% [a]	
Learning rate of BECCS	-75%-75% [a]	BF-BOF with BECCS
Initial cost of BECCS	-46%-46% [a]	
Learning rate of green H_2	-74%-74% [b]	4 technologies, including DRI-EAF with 100% green H_2, etc.
Initial cost of green H_2	-77%-153% [b]	
Learning rate of blue H_2	-75%-75% [b]	3 technologies, including DRI-EAF with 100% blue H_2, etc.
Initial cost of blue H_2	-51%-64% [b]	
Learning rate of electrolyser	-67%-67% [b]	Electrolyser-EAF Electrowinning-EAF
Initial cost of electrolyser	-40%-50% [b]	
Cumulative capacity of 6 novel components (CCS, BECCS, CCUS, green H_2, blue H_2, electrolyser) [c]	-50%-50% [c]	15 technologies involving novel components [c]
Price of iron ore	-30%-30% [d]	All 20 technologies
Price of scrap	-35%-35% [d]	All 20 technologies
Price of coke	-40%-40% [d]	All 20 technologies
Price of electricity	-50%-50% [d]	All 20 technologies

Note: [a] Uncertainty ranges are determined by the mean ±2 standard deviations (SD) of available literature data, where applicable. Percentages indicate the relative deviation of the uncertainty bounds from the baseline value. See Note S4 in SI Part A and Note S9 in SI Part B for details.

[b] Where data are insufficient or the mean ±2 SD range includes negative values, uncertainty is defined by the minimum and maximum values reported in the literature. See Note S5 in SI Part A and Note S10 in SI Part C for details.

[c] This row represents six separate uncertainty factors, each corresponding to the cumulative capacity of a novel component.

[d] Price data are sourced from Wind, Bloomberg, IRENA (2022), and our plant-level steel cost database. Uncertainty ranges are projected from historical price trends. See Note S6 in the SI for details.

Influence on 22 technologies costs. Figs. S11–S13 illustrate the sensitivity of each technology's cost to various cost-driving factors. Fig. S11 presents a single-factor sensitivity analysis, examining the impact of 22 key variables. These include four input prices, as well as the initial costs, learning rates, and cumulative capacities of six novel components (as detailed in Table S5). Fig. S12a-c provide a pairwise sensitivity analysis, demonstrating how technology costs respond to simultaneous variations in any two categories of learning curve parameters (initial costs, learning rates, and cumulative capacities) while holding the third category constant. The combined effect of joint variation across all three categories is shown in Fig. S12d, with an interactive version (Fig. S12d_three_factor_plot.html) available on the open-access platform (see links in Data Availability section). Finally, Fig. S13 presents a multi-factor sensitivity analysis that incorporates all three learning curve parameter categories along with input prices.

In brief, a 50% increase in the prices of iron ore, scrap, coke, and electricity would lead to cost increases for the most sensitive technologies of 20%, 34%, 10%, and 21%, respectively (Fig. S11). Specifically, BOF-based technologies are most vulnerable to iron ore price fluctuations, with their costs rising by 12-20%. EAF-based technologies, on the other hand, are most sensitive to scrap prices, resulting in a 14-34% increase in their costs. Compared to iron ore and scrap, higher electricity prices typically have a limited effect on steelmaking costs, leading to only a 1-10% cost increase for most BOF and EAF technologies, although electricity-intensive ones like electrolyser-EAF and electrowinning-EAF would see larger increases of 20-21%.

Moreover, a 50% increase in the initial costs (IC) of CCS, CCUS, BECCS, blue hydrogen (BH_2), green hydrogen (GH_2), and electrolyser results in corresponding technology cost rises of 6-7%, 5%, 12%, 5-10%, 6-12%, and 10%, respectively, when other factors remain unchanged (Fig. S11). When accounting for variations in the other two learning curve factors, i.e., learning rate (LR) and cumulative capacity (Xt), the resulting cost increases span a wider range: from 0.1% to 17% for CCS, 0.1% to 11% for CCUS, 0.8% to 26% for BECCS, 0.1% to 23% for BH_2, –6.0% to 43% for GH_2, and –12% to 78% for electrolysers (Fig. S12). For learning rates, a 50% increase in green hydrogen and electrolysers leads to cost reductions of up to 11% and 14%, respectively, under single-factor variation, while changes in LR for other novel components result in more modest cost decreases of 3-6%. When IC and Xt also vary, the cost reductions across all affected technologies expand to a range of –6% to 19%. The influence of

cumulative installed capacity is even smaller: a 50% increase in cumulative capacity reduces technology costs by less than 2% when other factors are constant, although the cost changes can extend to -18-65% with concurrent variations of IC and LR. These findings highlight that initial cost (IC) and learning rate (LR) have a substantially greater impact on cost projections than cumulative capacity (Xt).

Overall, the impact of fluctuations in major input prices on the most sensitive steelmaking costs (10–34%) is more substantial than that of variations in the initial costs of novel components (6–12%), learning rates (3-14%), or cumulative capacities (0.4-2%), as observed in the single-factor analysis (Fig. S11). However, these price changes have limited influence on the selection of lowest-cost technology options, because the prices of key materials and energy tend to affect multiple technologies simultaneously without significantly altering their relative cost differences.

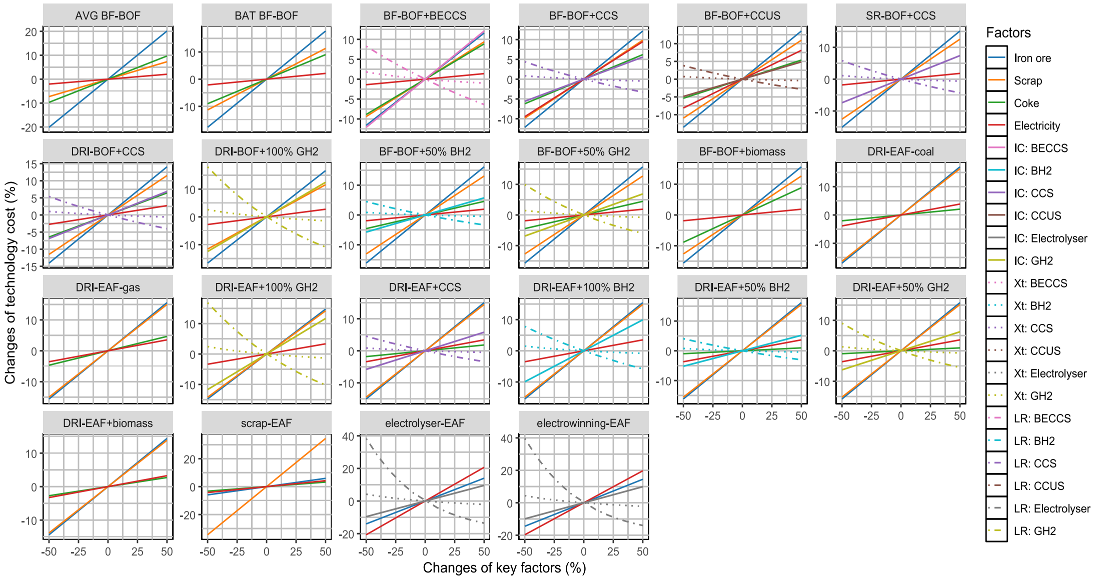

Fig. S11 Cost sensitivity of 22 technologies to key input prices, initial capital costs, learning rates, and cumulative capacities of novel components by 2050. Price fluctuations in iron ore, scrap, coke, and electricity are considered as four primary cost drivers in steelmaking. It also examines variations in initial cost (IC), learning rate (LR), and cumulative capacity (Xt) for novel components, including CCS, BECCS, CCUS, green hydrogen (GH_2), blue hydrogen (BH_2), and electrolysers. Colors differentiate input types and novel components, while line styles distinguish parameter types for each novel component: solid lines represent initial costs, dot-dashed lines indicate learning rates, and dotted lines denote cumulative capacities.

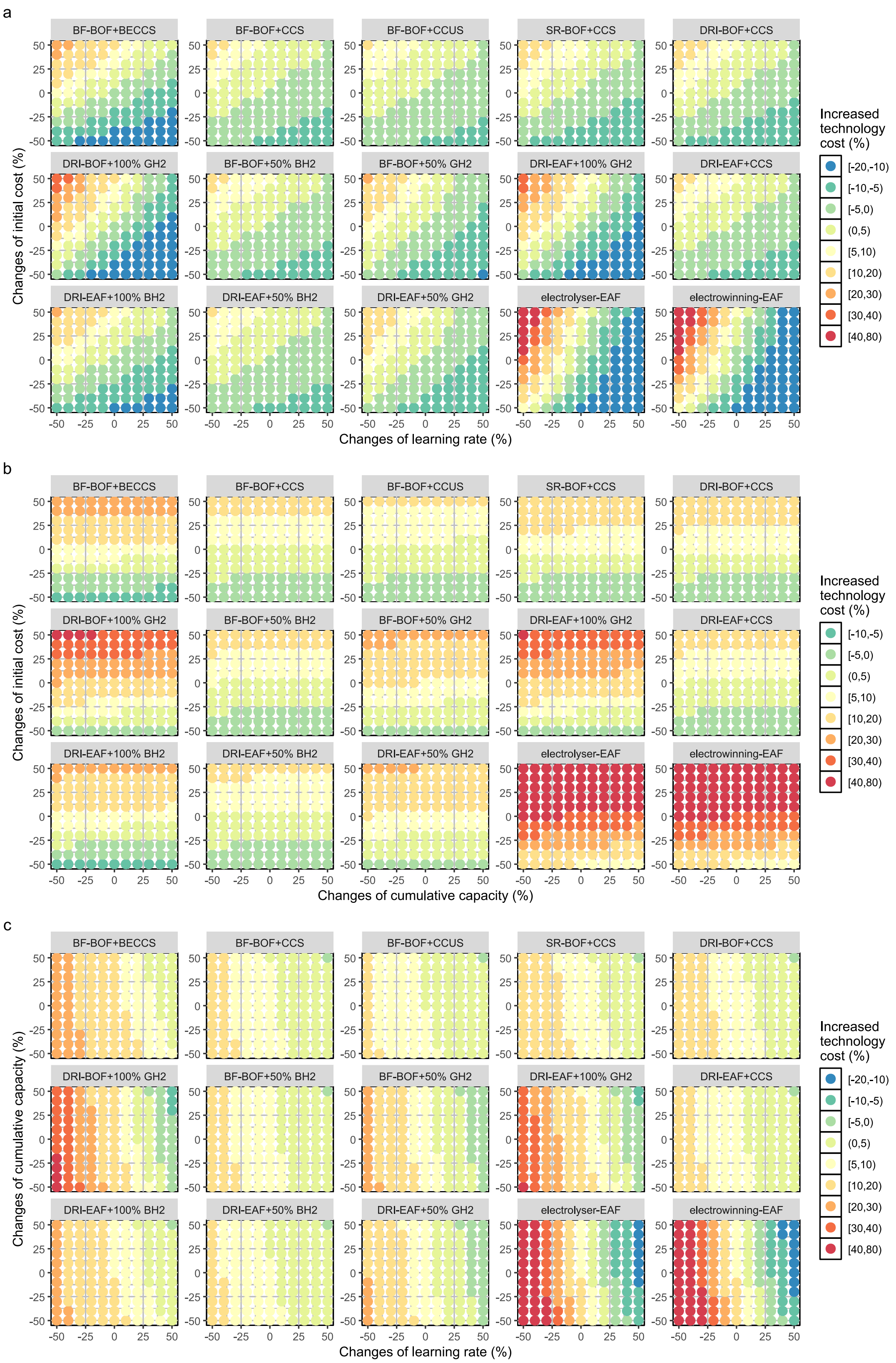

Fig. S12 Combined effects of learning curve parameter uncertainty on novel technology costs in 2050. This plot shows the pairwise cost sensitivity of novel technologies under simultaneous variation of learning curve parameters. Panels (a), (b), and (c) specifically

illustrate the interactive effects of initial cost (IC) and learning rate (LR); IC and cumulative capacity (Xt); and LR and Xt, respectively. For each panel, the third learning curve parameter not shown on the axes is held constant at a 50% increase. The novel components of each technology, including CCS, BECCS, CCUS, green hydrogen (GH_2), blue hydrogen (BH_2), and electrolysers, align with those presented in Fig. S11.

Multi-factor sensitivity analysis. We conducted a comprehensive multi-factor sensitivity analysis to assess the probabilistic cost distributions of 22 low-carbon technologies by accounting for the simultaneous uncertainty in all 22 cost drivers. For each parameter, we defined an uncertainty range of ±2 standard deviations (SD) around the mean and initially considered 100 possible values. This setup yields a theoretical input space with approximately 10^{44} possible combinations—far too large for practical computation. To generate a manageable yet representative sample, we applied the Latin Hypercube Sampling (LHS) method to select 1,000 input combinations. LHS is a stratified random sampling technique designed to efficiently explore high-dimensional input spaces with relatively few samples, ensuring broad coverage of each parameter's distribution (Saltelli et al., 2000; Lempert et al., 2013). By systematically varying all parameters simultaneously, LHS allows us to capture the combined effects of multiple uncertain inputs on technology cost projections—thereby revealing joint sensitivity in model outcomes.

The resulting cost distributions are presented in Fig. S13. When all cost drivers are varied together, the relative standard deviations (RSDs) of projected costs remain moderate, ranging from 8% to 24% across technologies. Technologies that rely on electrolysers—such as 100% green hydrogen, electrowinning-EAF, and electrolyser-EAF—show the highest RSDs (exceeding 15%), reflecting their heightened sensitivity to uncertainties in electrolyser costs and learning rates. To further characterize uncertainty, we also report the quartile coefficient of dispersion (QCD), which ranged from 6% to 16%. Technologies such as scrap-EAF, electrolyser-based systems, and 100% hydrogen had QCDs above 10%, indicating broader interquartile spreads, yet still within acceptable bounds. Taken together, the multi-factor sensitivity analysis confirms that our cost projections are robust even when accounting for the compounded uncertainties of all input parameters. These results reinforce the reliability of our key findings and conclusions.

We acknowledge as a limitation that our LHS implementation assumes statistical independence among input parameters, which may not reflect real-world interdependencies such as co-variation between initial costs, learning rates, cumulative capacities, and input prices. This assumption was necessary due to the absence of consistent empirical data on parameter covariances across technologies and regions. We encourage future research to address this issue by developing and incorporating better-informed correlation structures as more empirical evidence becomes available, to enhance the realism and precision of uncertainty propagation in techno-economic modeling.

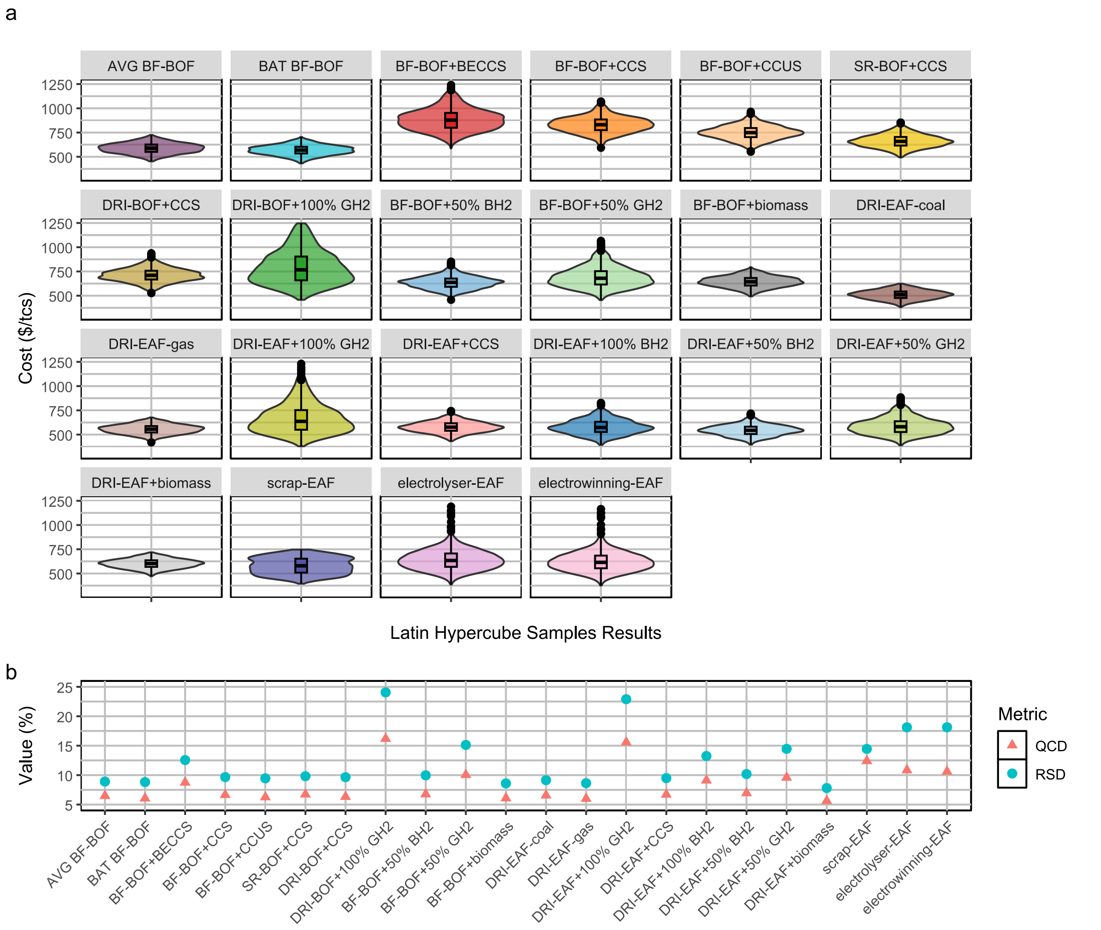

Fig. S13. Multi-factor sensitivity analysis of 2050 cost estimates for 22 technologies. All 22 cost drivers identified in Table S5 and Fig. S11 were simultaneously varied in the 1,000 cost combinations drawn by the Latin-hypercube sampling method from the input space (10^{44} possible combinations). a) Box-and-whisker plots display the median (line), interquartile range (box), and 5th–95th percentiles (whiskers) for each technology, with violin plots representing the probability density of cost distributions. b) Statistical summary of the distributions in (a), reporting the relative standard deviation (RSD) and quartile coefficient of dispersion (QCD) for each technology. RSD is defined as the ratio of the standard deviation to the mean, while QCD is the interquartile range (Q3 − Q1) divided by the sum of the third and first quartiles (Q3 + Q1). Both RSD and QCD values are shown as percentages in this plot.

Influence on the least-cost technology choice. Figs. S14-S15 illustrate the sensitivity of the least-cost technology options to variations in input prices, initial costs, and learning rates of novel components. All input data are represented as global averages, corresponding to technology choices suitable for the average steel plant worldwide. The results indicate that, in most cases, the cost-effective low-carbon and zero-carbon technology choices are consistent and robust.

Specifically, for a BOF and an EAF steel plant at the global average level, the most

cost-effective low-carbon options are respectively BAT BF-BOF and DRI-EAF injected with 50% blue hydrogen (DRI-EAF+50%BH_2), while the least-cost zero-carbon options are SR-BOF+CCS and DRI-EAF+CCS, respectively. However, with fluctuations of prices, initial costs and learning rates, the cheapest technologies might shift to DRI-BOF+100%GH_2, DRI-EAF+100%GH_2, DRI-EAF+50%GH_2 and DRI-EAF+100%BH_2. For instance, if the learning rate or initial cost of green H_2 varies by more than 20-40%, the cheapest zero-carbon option will shift from CCS to green hydrogen for both BOF and EAF plants (i.e., from SR-BOF+CCS to DRI-BOF with 100% GH_2, and from DRI-EAF+CCS to DRI-EAF with 100% GH_2). In addition, partial injection of green hydrogen (DRI-EAF with 50% GH_2) becomes a more cost-effective low-carbon option than blue hydrogen (DRI-EAF with 50% BH_2) for EAF plants.

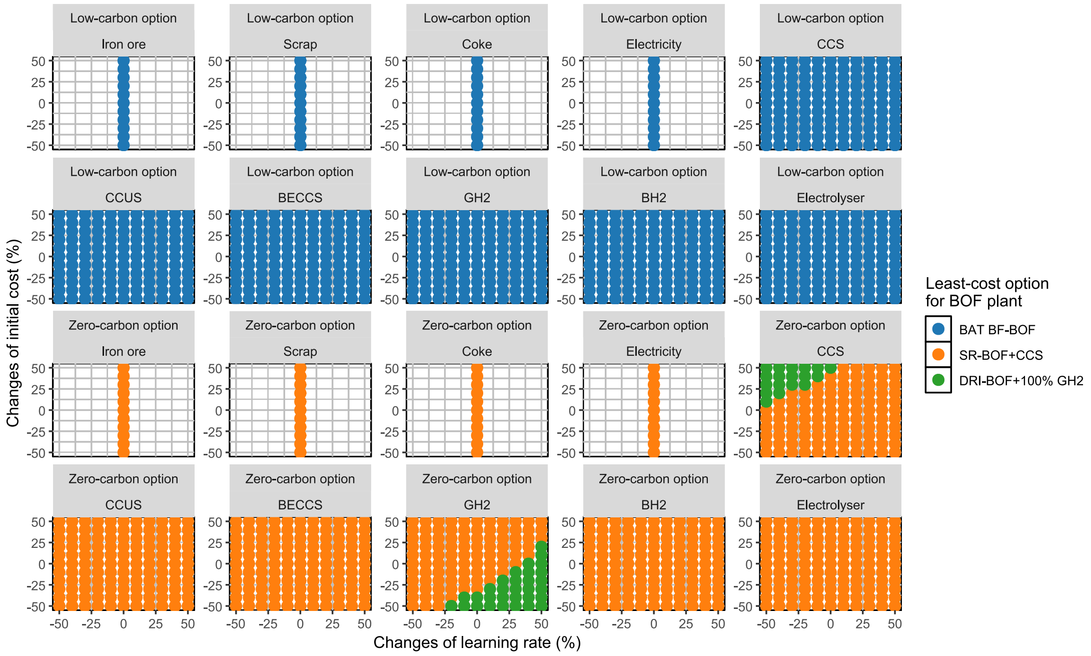

Fig. S14 Least-cost technology option for BOF steel plants on a global-average basis in 2050. The initial cost, learning rate, and prices are set at the global average level. Each subFig. is titled to identify the technology category as either low-carbon or zero-carbon. The subtitle specifies the key component whose price, initial cost, or learning rate is subject to change. Cumulative capacity is assumed unchanged due to its limited impact on technology costs. Points represent the least-cost technology option under various factor changes, with colors indicating the technology name. Scrap-EAF is excluded, as it is generally the lowest-cost zero-carbon option for BOF plants in the absence of supply constraints.

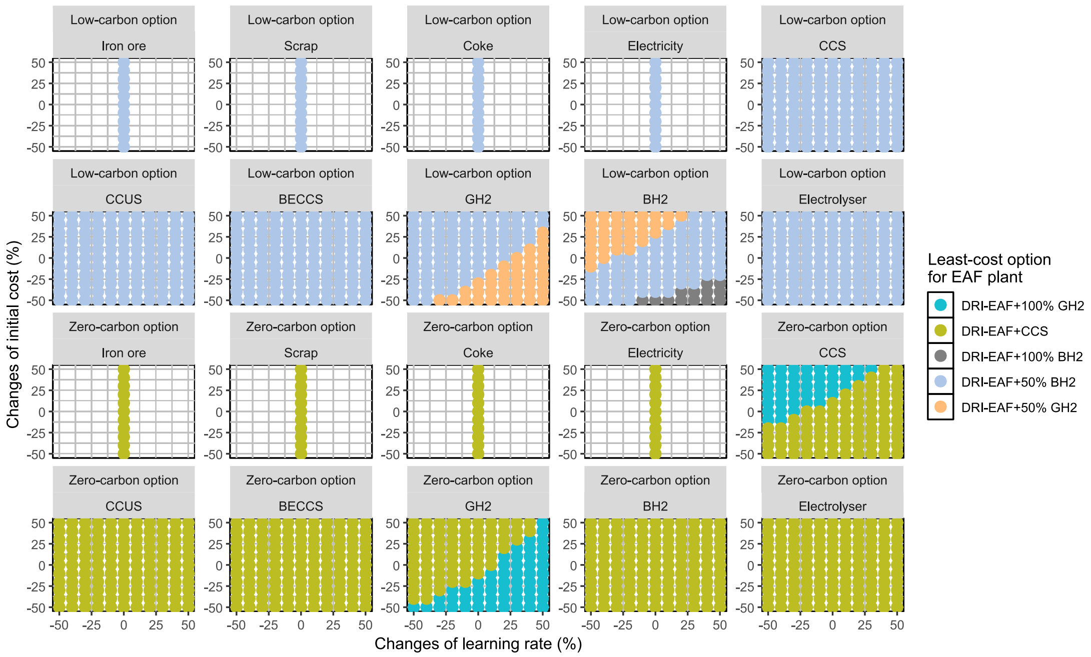

Fig. S15 Least-cost technology option for EAF steel plants on a global-average basis in 2050. The initial cost, learning rate, and prices are set at the global average level. Each subFig. is titled to identify the technology category as either low-carbon or zero-carbon. The subtitle specifies the key component whose price, initial cost, or learning rate is subject to change. Cumulative capacity is assumed unchanged due to its limited impact on technology costs. Points represent the least-cost technology option under various factor changes, with colors indicating the technology name. Scrap-EAF is excluded as it is generally the lowest-cost zero-carbon option when supply constraints are not considered.

Note S8. Sensitivity tests of decarbonization pathways, abatement potential and cost.

Identification of the key factors. Based on the sensitivity analyses of technology costs and cost-effective technology choices (as shown in Note S7), we identified scrap price, and the initial cost and learning rate of green hydrogen and CCS as the most influential factors shaping the least-cost technology pathway. Additionally, we considered the potential changes in electricity prices and emissions due to energy system transition, as well as longer retrofitting intervals. Therefore, twelve sensitivity scenarios were designed to assess the impacts of these key factors on global cost-effective pathways, abatement potential, and abatement costs (Figs. S16–S28). The scenario input parameters, reflecting uncertainties in the literature or historical data (see details in Notes S4–S6), are summarized in Table S6.

Table S6. Scenarios parameters for sensitivity analysis of technology pathways.

Scenario Name	Description
Baseline	Base price and cost with 20-year retrofit cycle [c]
S35	+35% increase in scrap price [a]
ICH2	+50% increase in initial cost of green hydrogen [b]
LRH2	+50% increase in learning rate of green hydrogen [b]
ICCCS	+50% increase in initial cost of CCS [b]
LRCCS	+50% increase in learning rate of CCS [b]
E30	−30% decrease in electricity price [c]
E50	+50% increase in electricity price [c]
All	Combination of all above price and cost variations [ab]
RY35	Retrofit cycle set to every 35 years [c]
RY50	Retrofit cycle set to every 50 years [c]
All_RY35	All price and cost variations combined with a 35-year retrofit cycle [abc]
Scope 1+2	Emissions include both scope 1 and scope 2 [c]

Note: [a] Derived from the upper bound of historical price. See Note S6 in SI Part A for details.
[b] Approximated using the relative standard deviation (RSD) of literature values. See Note S4 in SI Part A and Notes S9-S10 in SI Part B and C for details.
[c] Based on IEA (2020)[20] and IRENA (2022)[70].

Influence of prices, initial cost and learning rate. Figs. S19–S25 present the global abatement potential and cost of the least-cost technology pathway under varying input parameters, including scrap price (S35 scenario), electricity price (E30 and E50 scenarios), and the initial costs and learning rates of green hydrogen (ICH2 and LRH2 scenarios) and CCS (ICCCS and LRCCS scenarios). Fig. S26 summarizes the combined effects of all price and cost changes (All scenario), while Fig. S27 incorporates these changes alongside an extended retrofit cycle (All_RY35 scenario). In addition, the effects of extended retrofit intervals (RY35 and RY50 scenarios) and

the inclusion of indirect emissions (Scope 1+2 scenario) are shown in Figs. S18 and S28, respectively. Figs. S16–S17 provide an overview of the differences between all sensitivity scenarios and the baseline results discussed in the main text.

In brief, compared to the cost-effective pathway in the main text (Fig. 5),

1) S35 scenario: a 35% increase in scrap price reduces the abatement contribution of scrap-EAF while increasing that of SR-BOF+CCS across all three policy scenarios. Additionally, it results in greater deployment of BAT BF-BOF in medium scenario and DRI-BOF+CCS in early scenario. Furthermore, the abatement costs for nearly all BOF-based technologies rise significantly due to their dependence on scrap feedstock, while the costs for DRI-EAF-based technologies, such as DRI-EAF+CCS, experience a slight increase due to their accelerated and expanded deployment. Overall, the average abatement costs notably increase to \$44/tCO2, \$45/tCO2, and \$68/tCO2 in late, medium, and early scenarios, respectively (Fig. S19).

2) ICH2 scenario: a 50% increase in the initial cost of green hydrogen increases the abatement contribution of SR-BOF+CCS by 0.3 Gt CO_2 in each scenario, with additional increases of 0.3–0.4 Gt from DRI-EAF with 50% blue hydrogen and DRI-EAF with biomass in medium scenario, and 0.1–0.2 Gt from DRI-EAF+CCS and BF-BOF+CCUS in early scenario. These shifts reflect the substitution of more expensive green hydrogen technologies with economically viable options such as CCS, blue hydrogen, CCUS, and biomass. Consequently, the average cost of abatement remains largely unchanged across all scenarios (Fig. S20).

3) LRH2 scenario: a 50% rise in the learning rate of green hydrogen enhances the abatement potential of green hydrogen-based technologies as substitutes for CCS. Specifically, DRI-BOF with 100% green hydrogen increases by 1 Gt, 1 Gt, and 11 Gt CO_2 in late, medium, and early scenarios, respectively; DRI-EAF with 100% green hydrogen increases by 1 Gt in early scenario; and DRI-EAF with 50% green hydrogen increases by 0.3 Gt in medium scenario. The total cumulative CO2 abatement potential remains comparable to the pathway in the main text, while the average abatement cost decreases slightly by \$3–9/tCO_2, reaching \$22/tCO_2, \$21/tCO_2, and \$45/tCO_2 in late, medium, and early scenarios, respectively (Fig. S21).

4) ICCCS scenario: a 50% rise in the initial cost of CCS increases the abatement contribution of BF-BOF+CCUS and DRI-EAF with 100% green hydrogen in all scenarios by 2-6 Gt and 0.9-1Gt CO2, respectively, replacing the currently more expensive SR-BOF+CCS and DRI-EAF+CCS. In the early scenario, DRI-BOF with 100% green hydrogen provides an additional 6.5 Gt of abatement as a cost-effective alternative to early deployment of CCS technologies. With the loss of CCS's cost advantage, the decarbonization pathway shifts toward greater reliance on more expensive green hydrogen technologies, leading to an increase in average

abatement costs by \$4–18/tCO_2, reaching \$33/tCO_2, \$29/tCO_2, and \$72/tCO_2 in the late, medium, and early scenarios, respectively (Fig. S22).

5) LRCCS scenario: a 50% increase in learning rate of CCS has the opposite effect of increasing its initial cost. In the early scenario, abatement from DRI-EAF+CCS and SR-BOF+CCS increases by 0.1 Gt CO_2 and 0.4 Gt CO_2, respectively, replacing more expensive green hydrogen and CCUS technologies. Moreover, the accelerated cost reduction enables SR-BOF+CCS to become cheaper than existing BF-BOF in some plants, facilitating voluntary technology transition and mitigation even under the BAU scenario. As a result, the additional abatement from SR-BOF+CCS in policy scenarios relative to BAU is reduced. The average abatement cost decreases by \$2–9/tCO_2 across all scenarios, reaching \$22/tCO_2 in late and medium scenarios and \$44/tCO_2 in early scenario (Fig. S23).

6) E30 scenario: a 30% decrease in electricity price from solar PV reduces the abatement contribution of SR-BOF+CCS, which is replaced by increased mitigation from BF-BOF+CCUS (2.0–3.5 Gt CO_2) and DRI-BOF with 100% green hydrogen (0.1–0.5 Gt CO_2) across all three scenarios. Consequently, the average abatement costs in late, medium, and early scenarios slightly decrease by \$1-2/tCO_2, compared to the corresponding scenarios in the main text (Fig. S24).

7) E50 scenario: a 50% increase in electricity price due to wind electricity leads to increased deployment of SR-BOF+CCS across all scenarios, while reducing the adoption of DRI-BOF with 100% green hydrogen in late and medium scenarios, and of BF-BOF+CCUS in early scenario. Therefore, the average abatement costs increase by \$1-3/tCO_2, reaching \$29/tCO_2, \$26/tCO_2, and \$56/tCO_2 in late, medium, and early scenarios, respectively (Fig. S25).

8) All scenario: the combined effect of increased scrap and electricity prices, along with higher initial costs and learning rates for green hydrogen and CCS, leads to an increase in cumulative abatement by 1.7 Gt and 1.2 Gt CO_2 in late and medium scenarios, respectively. Meanwhile, the average abatement cost rises by \$21–27/tCO_2 across all scenarios, reaching \$47/tCO_2, \$48/tCO_2, and \$81/tCO_2 in late, medium, and early scenarios, respectively. The abatement contributions of DRI-BOF with 100% green hydrogen (increased by 1.1–8.7 Gt CO_2), Scrap-EAF (2.6 Gt CO_2), and BF-BOF with CCUS (1.2–2.4 Gt CO_2) rise across all scenarios, with DRI-EAF using 100% green hydrogen also contributing an additional 0.8 Gt CO_2 in the early scenario. In contrast, the abatement potential of SR-BOF with CCS decreases substantially by 3–11 Gt CO_2 in all scenarios. These results suggest that under the combined influence of multiple factors, CCS-based technologies become less cost-competitive, while alternative options such as green hydrogen, Scrap-EAF, and CCUS gain economic viability (Fig. S26).

9) All_RY35 scenario: extending the retrofitting cycle significantly reduces cumulative abatement by 42–60% relative to the baseline results presented in the

main text (or 48–60% compared to the All scenario), while average abatement costs rise further to \$53/tCO_2 in the late and medium scenarios, and to \$52/tCO_2 in the early scenario. The abatement contributions of DRI-BOF with 100% green hydrogen and BF-BOF with CCUS consistently increase by 0.4–5.0 Gt CO_2 across all scenarios, whereas that of Scrap-EAF declines by 5–6 Gt CO_2 due to constrained scrap availability during a delayed and compressed retrofitting window approaching the net-zero target year (Fig. S27).

Influence of electricity transition. Electricity-based steelmaking strategies have gained substantial momentum, such as DRI-EAF and scrap-EAF, with great hope placed on using renewable electricity for decarbonizing the iron and steel industry[5]. The transition of electricity generation forms may have influence on the carbon mitigation potential and cost-effectiveness of corresponding steelmaking technologies[6], thus affecting the exploration of the decarbonization pathways for the iron and steel plants. Therefore, we conducted two sensitivity tests focusing on changes in electricity prices and indirect emissions to discuss the impact of electricity transition on the mitigation potential and cost of each technology.

1) Changes in electricity prices:

Due to the spectacular cost reduction of renewable electricity, till 2021, the costs of electricity generation by solar PV and onshore wind are already 29% lower and merely 71% higher, respectively, than those of fossil fuel electricity[70]. Therefore, we conducted a sensitivity test of global mitigation potential and cost to the varied electricity price in range of -30% and +50% (Figs. S24-S25).

In brief, a 50% change in electricity price alters the costs of most technologies by only 1–10%, and up to 21% for the highly electricity-intensive direct electrolysis route (Fig. S11). Furthermore, changes in electricity prices below 50% have limited impact on the selection of least-cost decarbonization technologies, except for scrap-EAF (Figs. S14-S15). As a result, a 30% decrease in electricity price leads to the substitution of SR-BOF+CCS with BF-BOF+CCUS and DRI-BOF with 100% green hydrogen, reducing the abatement potential of SR-BOF+CCS by 16–36% (2–4 Gt CO_2) across all scenarios. The average abatement cost decreases slightly to \$24/tCO_2, \$23/tCO_2, and \$52/tCO_2 in the late, medium, and early scenarios, respectively. Conversely, a 50% increase in electricity price enhances the abatement potential of SR-BOF+CCS by 4-7% across all scenarios, raising the abatement cost from \$25-26/tCO_2 to \$26-29/tCO_2 in the late and medium scenarios, and from \$54/tCO_2 to \$57/tCO_2 in the early scenario (Figs. S16-S17 and Figs. S19-S25).

2) Impact of indirect emissions:

In the main text, we focused on direct emissions of steel plants and decarbonization technologies, which will not be affected by electricity transition. However, given the rapid development of renewable electricity, we added a sensitivity test for indirect CO_2

emissions, which is estimated based on the IEA's projection of carbon intensities of global electricity generation[20], and on electricity consumption factors of decarbonization technologies as reported in previous studies[5,6,11,71].

The results are presented in Fig. S28 and summarized in Table S7. In brief, when indirect emissions from electricity consumption are considered, the annual CO_2 emissions of the global steel sector in 2050 increase significantly by 19–117% across all scenarios. However, cumulative abatement potential and average abatement cost exhibit only minor variations, ranging from –0.2% to +8% and –8% to +0.3% respectively, compared to results based solely on direct emissions. Moreover, the key technologies contributing most to global decarbonization remain consistent, with only limited changes in their individual abatement potential and cost. These findings indicate the limited influence of indirect emissions on global cost-effective decarbonization pathway.

Table S7. The influence of indirect emissions on global abatement potential and cost.

Results	BAU	Late scenario	Medium scenario	Early scenario
2050 emissions ($Gt\ CO_2$)	2.8→3.4 (19%)	1.3 →1.8 (35%)	1.1→1.5 (44%)	0.3→0.6 (117%)
Cumulative abatement ($Gt\ CO_2$)	3.3→3.3 (0%)	17→17 (-0.2%)	26→26 (-0.1%)	56→61 (8%)
Average abatement cost ($\$/tCO_2$)	72→72 (0%)	26→26 (0.3%)	25→25 (0.1%)	54→50 (-8%)
Top mitigation technologies	BAT BF-BOF.	SR-BOF+CCS, Scrap-EAF.	SR-BOF+CCS, Scrap-EAF, BAT BF-BOF.	SR-BOF+CCS, DRI-EAF+CCS, Scrap-EAF.

Note: Values before the arrows represent the results for direct emissions (Fig. 5 in the main text), while values after the arrows represent the results when indirect emissions are included (Fig. S28). Numbers in parentheses indicate percentage changes.

Influence of retrofitting intervals. According to IEA (2020)[20], the typical capital cycle for steel plants is 20 years, suggesting that retrofitting equipment older than 20 years would not result in stranded assets or additional economic losses. However, to conduct a comprehensive sensitivity analysis, we simulated scenarios with extended retrofitting intervals of 35 and 50 years for comparison. For each assumption of retrofitting year, we obtained the most cost-effective decarbonization pathways for each steel plant worldwide, recalculated their cumulative abatement potential and costs on a plant-by-plant basis, and finally aggregated the plant-level results to regional and global levels.

The comparison of different retrofitting intervals reveals that retrofitting every 20 years resulted in the highest abatement potential and the lowest average abatement cost, both worldwide and in key regions (Fig. S18). It highlights the importance of early

retrofitting for cost-effective global mitigation and achieving climate goals. In this context, even though some blast furnaces (BFs) may operate for up to 50 years with high emissions currently, meeting national net-zero targets requires an expedited decarbonization transition in the future. Therefore, we assumed a 20-year retrofit interval in our main text to explore the cost-effective technology pathway aligned with national carbon neutrality targets.

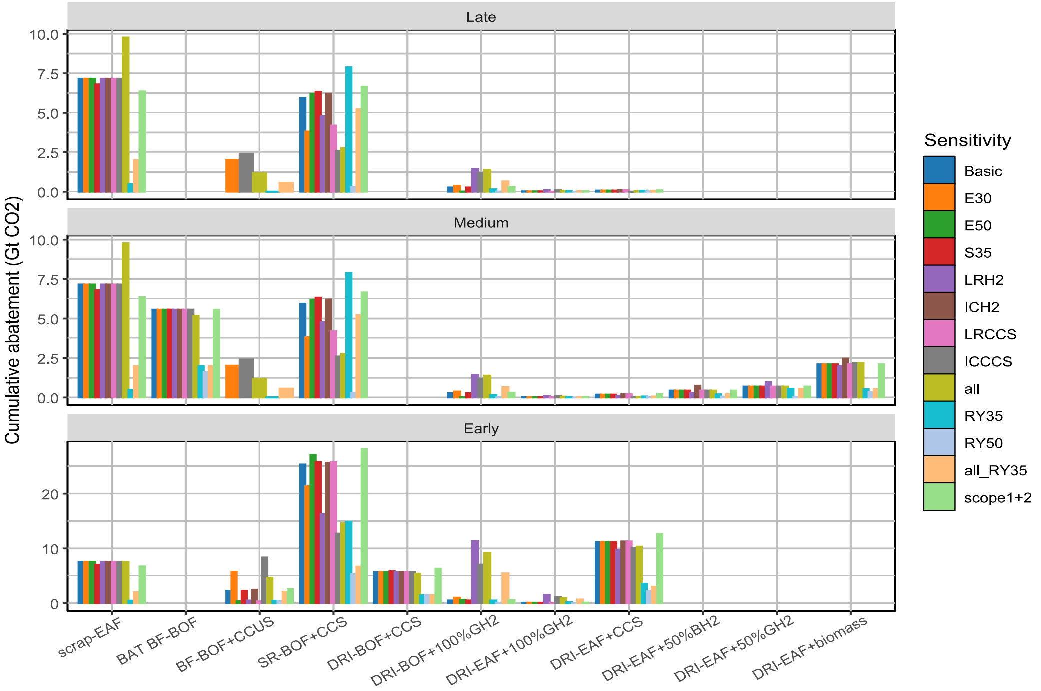

Fig. S16 Difference in global abatement potential under varying assumptions. The basic scenario refers to the one used in the main text, while the other scenarios incorporate variations in scrap prices (S35), green hydrogen costs (ICH2 and LRH2), CCS costs (ICCCS and LRCCS), and electricity prices (E30 and E50), as well as indirect emissions (Scope 1+2), extended retrofitting intervals (RY35 and RY50), and the combined effects of all price and cost changes, both with (All_RY35) and without (All) extended retrofitting intervals. Detailed assumptions are provided in Table S6.

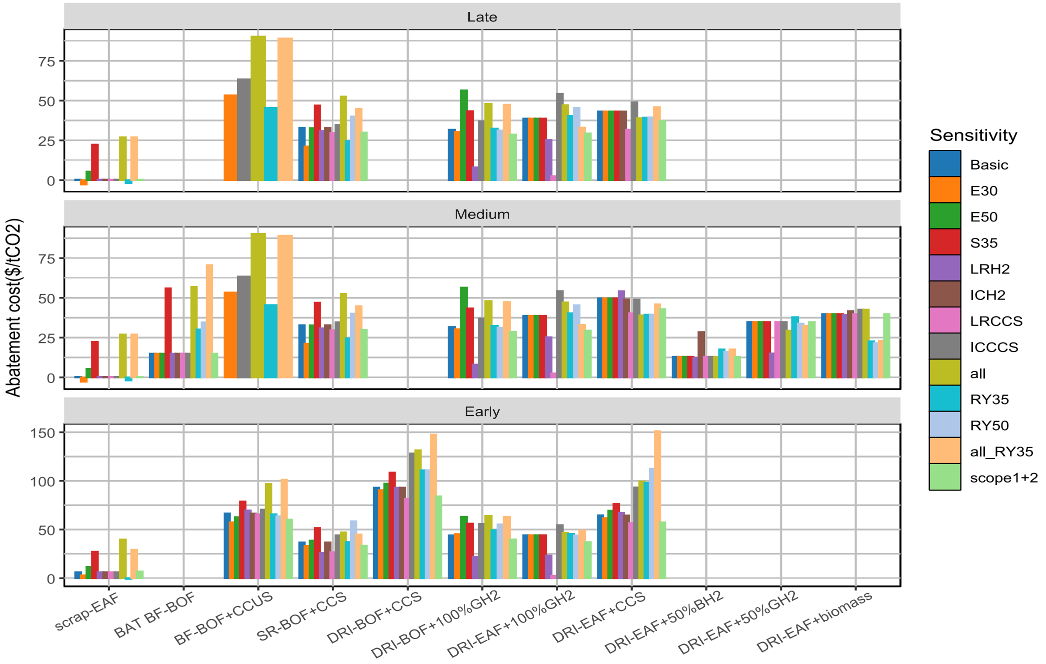

Fig. S17 Difference in global abatement cost under varying assumptions. The basic scenario refers to the one used in the main text, while the other scenarios incorporate variations in scrap prices (S35), green hydrogen costs (ICH2 and LRH2), CCS costs (ICCCS and LRCCS), and electricity prices (E30 and E50), as well as indirect emissions (Scope 1+2), extended retrofitting intervals (RY35 and RY50), and the combined effects of all price and cost changes, both with (All_RY35) and without (All) extended retrofitting intervals. Detailed assumptions are provided in Table S6.

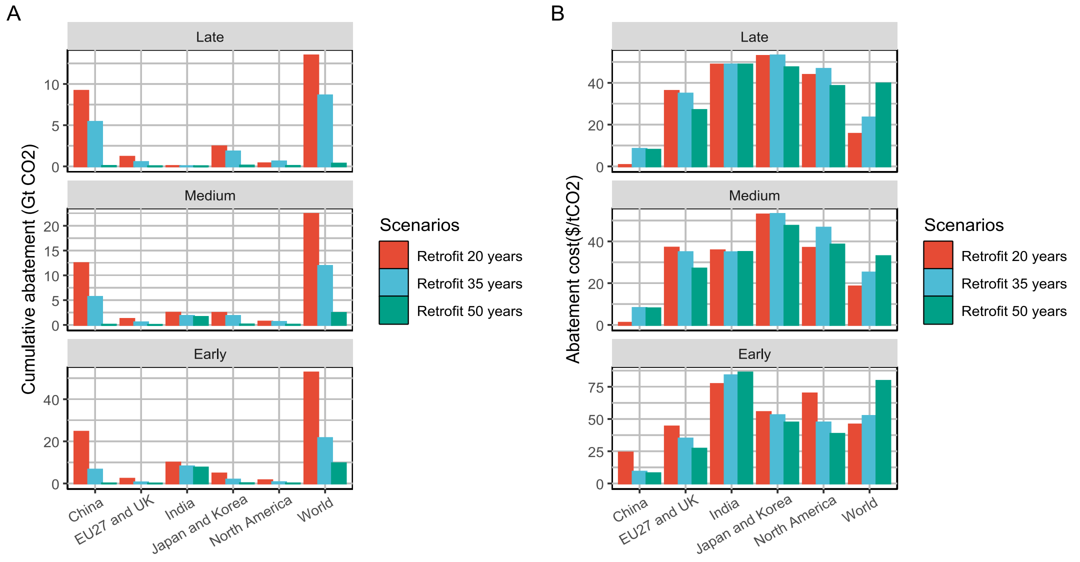

Fig. S18 Global abatement potential and abatement cost of the cost-effective decarbonization pathway with retrofitting every 20, 35 and 50 years. A) and B) present the abatement potential and abatement cost of key regions and the world, respectively. The Late, Medium, and Early scenarios represent different paces of zero-carbon technology deployment, as outlined in the main text.

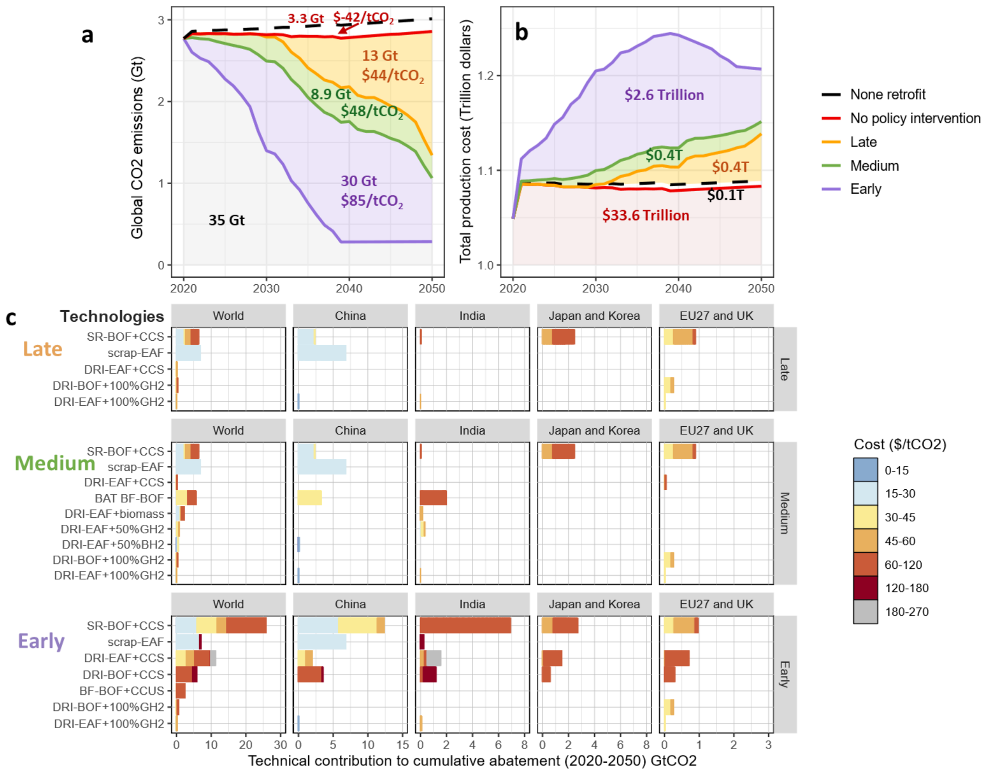

Fig. S19 Global abatement potential and cost with a 35% increase in scrap price (S35 scenario). Numbers in yellow, green, and purple indicate the differences between adjacent scenarios in terms of a) cumulative CO_2 abatement and average abatement cost, and b) increases in total production cost, corresponding to the areas between neighboring solid lines. c) presents the abatement potentials and abatement costs of various low and zero-carbon technologies across the globe and major regions over 2020-2050 under the Late, Medium, and Early policy scenarios, compared to reference scenarios without policy intervention.

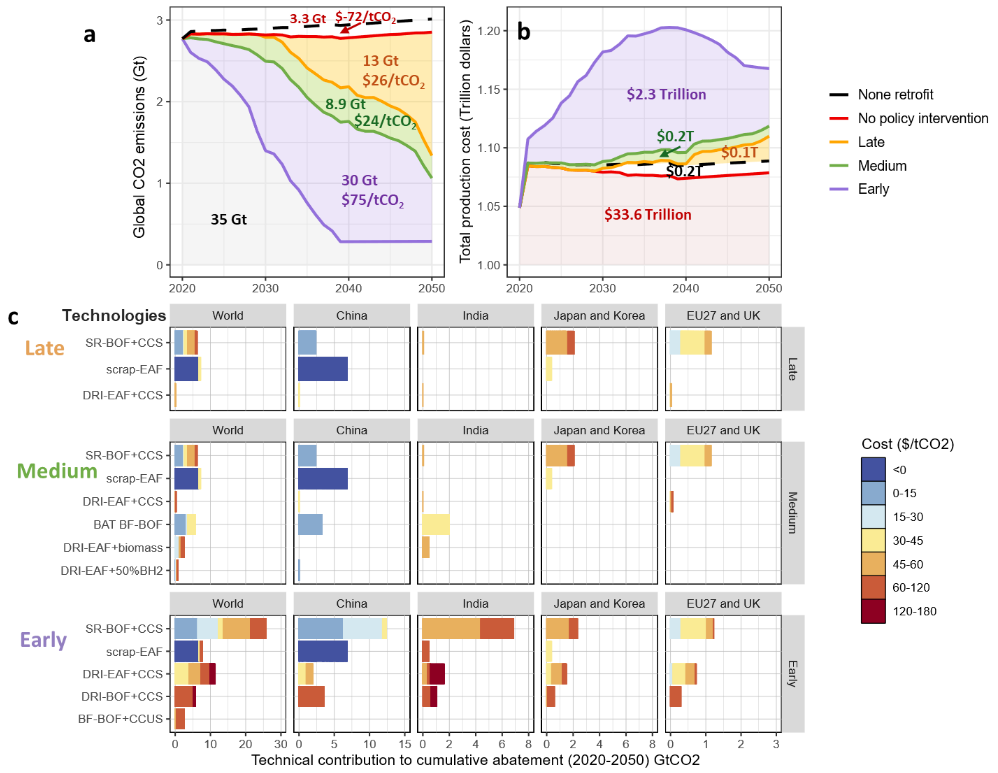

Fig. S20 Global abatement potential and cost with a 50% increase in initial cost of green hydrogen (ICH2 scenario).

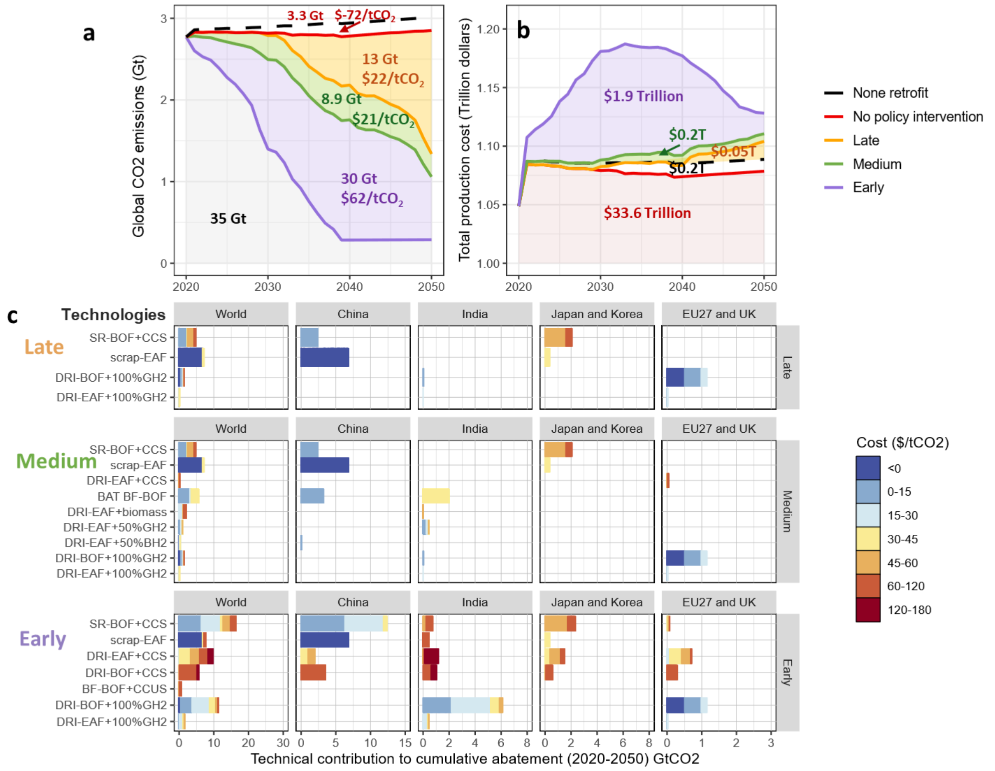

Fig. S21 Global abatement potential and cost with a 50% increase in learning rate of green hydrogen (LRH2 scenario).

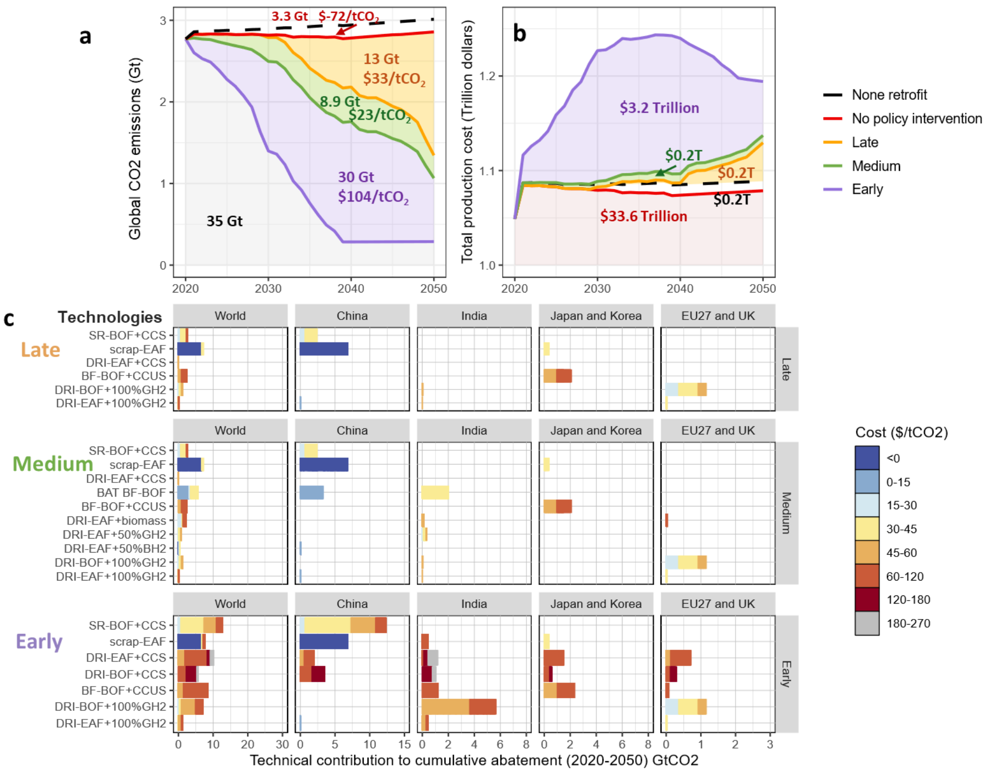

Fig. S22 Global abatement potential and cost with a 50% increase in initial cost of CCS (ICCCS scenario).

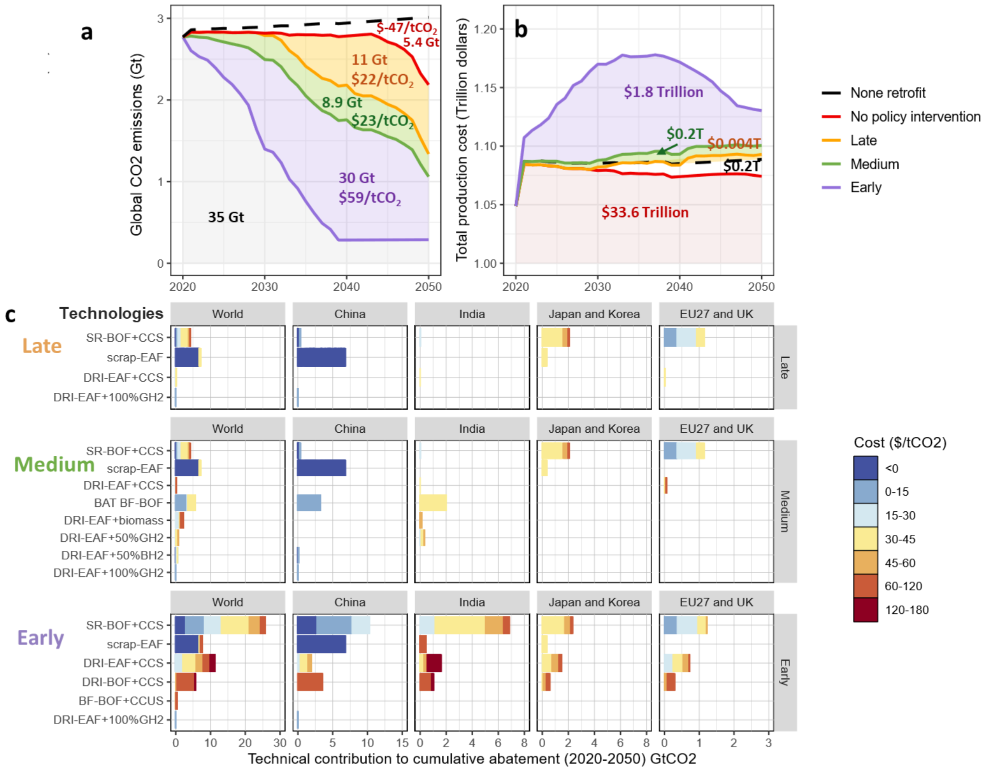

Fig. S23 Global abatement potential and cost with a 50% increase in learning rate of CCS (LRCCS scenario).

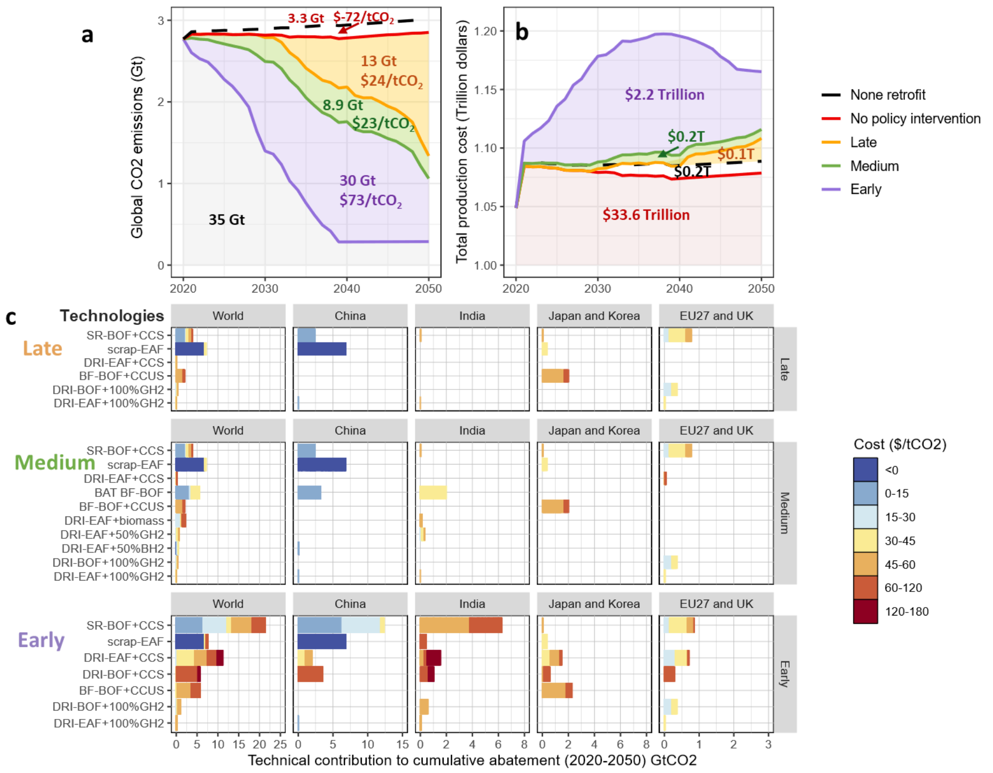

Fig. S24 Global abatement potential and cost with a 30% reduction in electricity price due to renewable electricity application (E30 scenario).

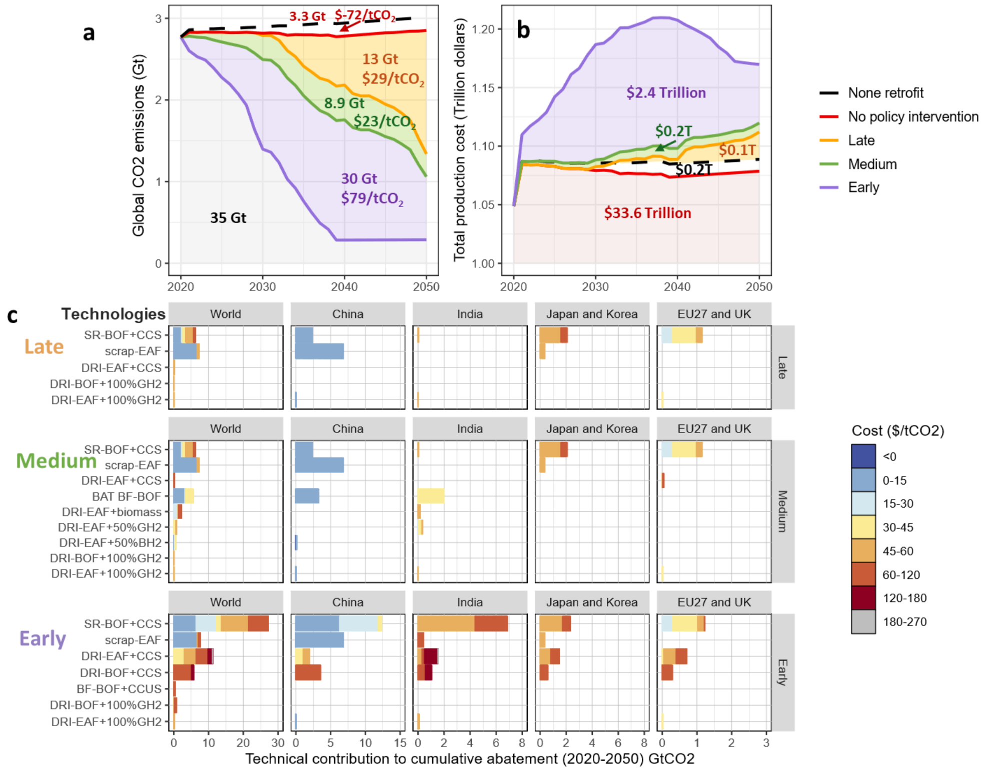

Fig. S25 Global abatement potential and cost with 50% increase in electricity price due to renewable electricity application (E50 scenario).

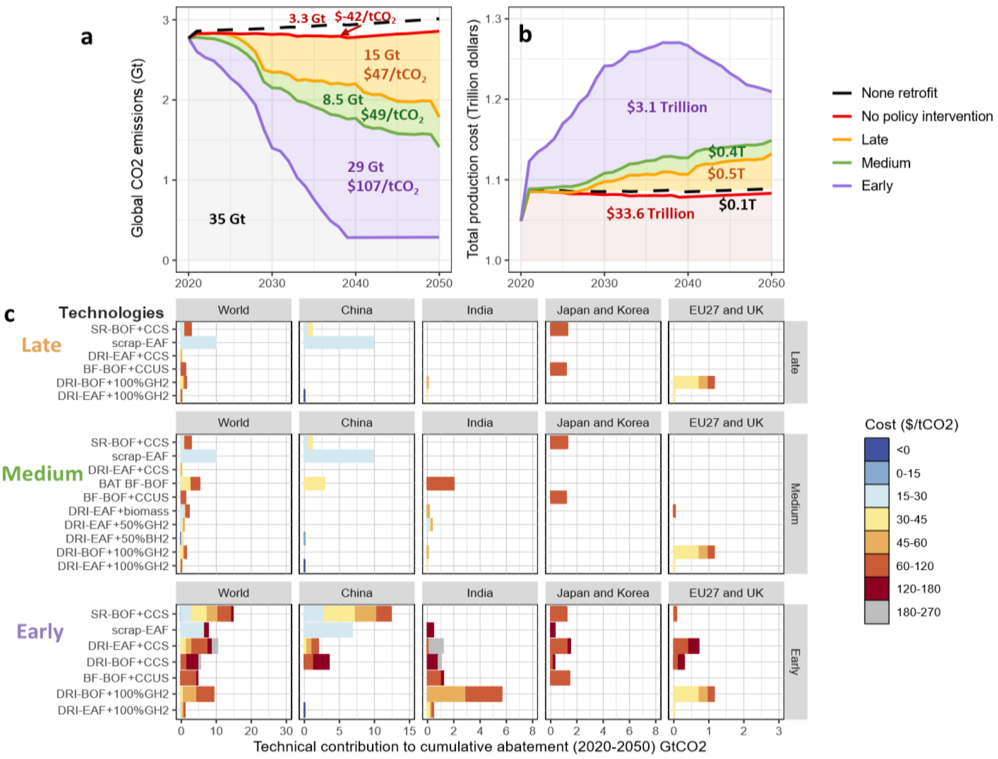

Fig. S26 Global abatement potential and cost under simultaneous changes in scrap and electricity prices, as well as the initial costs and learning rates of green H2 and CCS (All scenario).

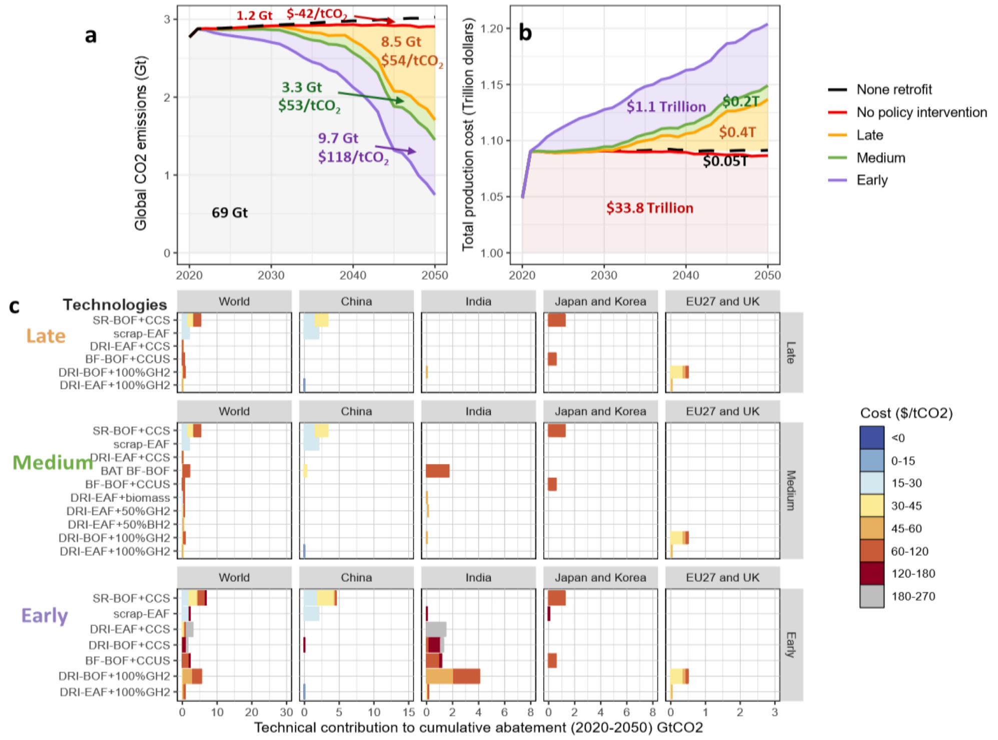

Fig. S27 Global abatement potential and cost under simultaneous changes in scrap and electricity prices, the initial costs and learning rates of green hydrogen and CCS, and an extended retrofit cycle of 35 years (All_RY35 scenario).

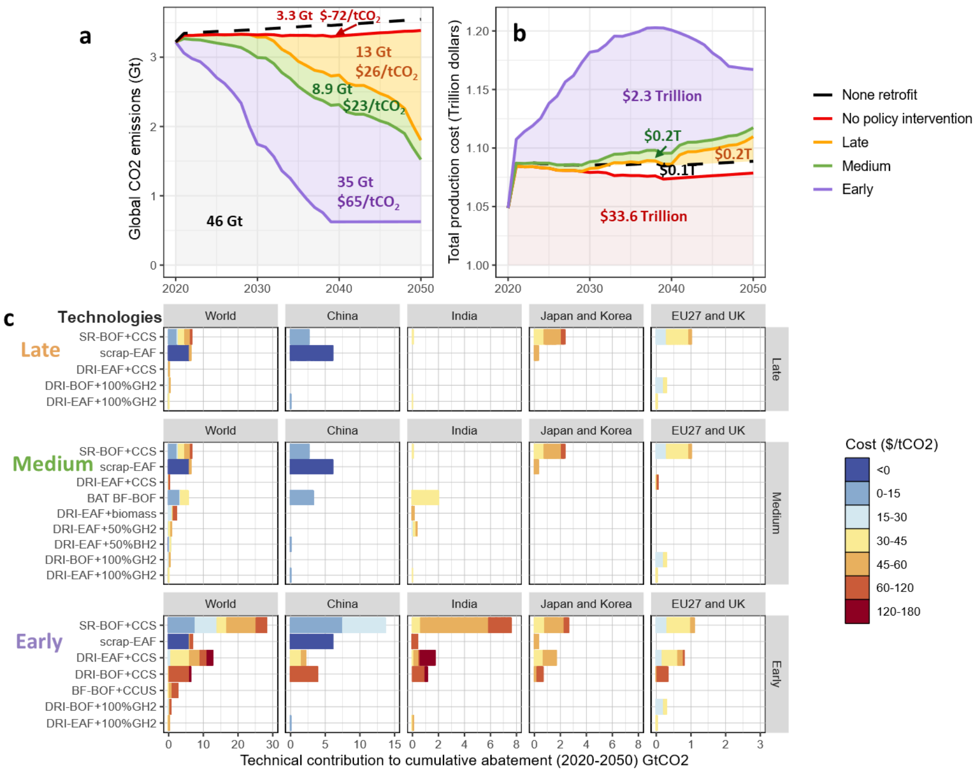

Fig. S28 Global abatement potential and cost including both Scope 1 and Scope 2 emissions (Scope 1+2 scenario).

Table S8. Summary of data source information.

Data	Description	Source	Website
Plant-level production database	Covering 4900+ operating steelmaking and processing plants with 20000+ facilities in 127 countries worldwide	World Crude Steel Capacity and Production Database	https://www.steelonthenet.com/plant.html
Plant-level cost database	Providing the production cost and cost structures of thousands of individual steel plants worldwide	Global Iron and steel Cost Database; Metalinfo Steel Cost Database	https://www.transitionzero.org/products/global-steel-cost-tracker; http://www.metalinfo.cn
Learning rates and initial costs	The learning rates and initial costs collected from high-quality literature for cost projections	Systematic literature review. See Note S9-S10 in Supplementary Information on CCS and H2 for details	
Technology parameters	Emission factors, input consumption factors, and technical readiness of each studied decarbonization technologies	Refer to previous studies. See the Methodology section in main text for details.	

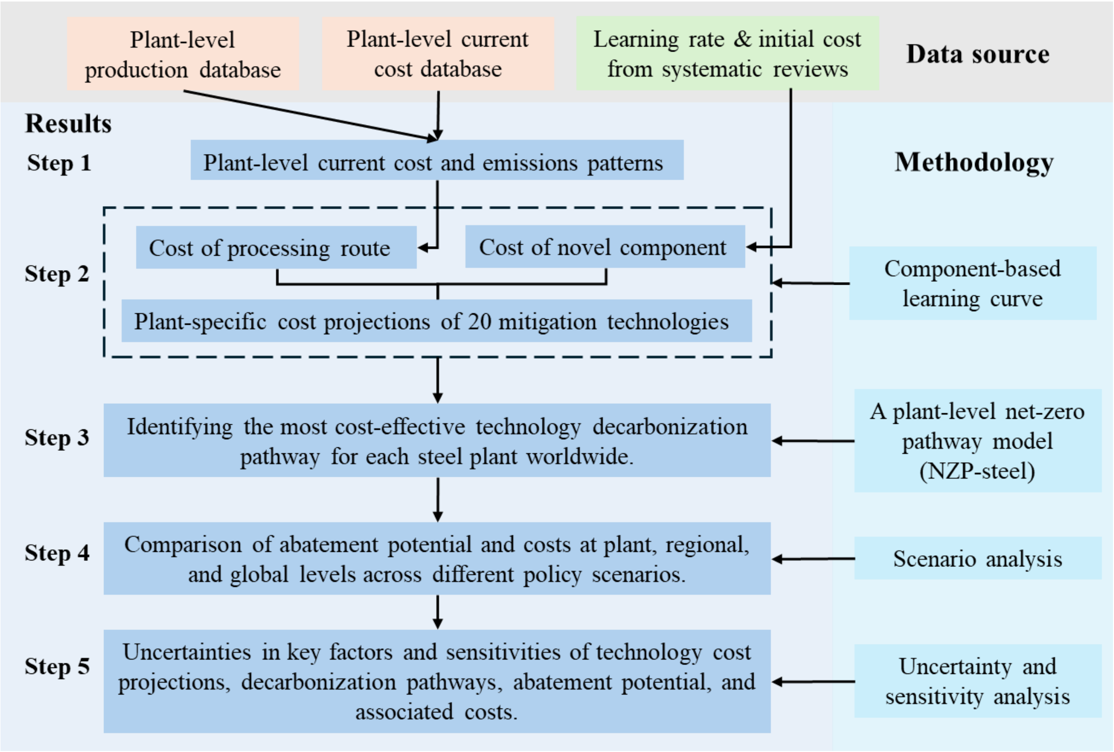

Fig. S29 Framework of data source, methodology and results in this study. The study draws on two primary data types: plant-level production and cost databases (orange) and parameters from literature and authoritative reports (green). The plant-level databases serve as the core data foundation, integrated with various methodologies (shown in cyan) to generate results across all stages (shown in blue). Data from extensive literature reviews primarily inform cost projections for novel components, determining key input parameters for the learning curve, such as learning rates and initial costs. Sources for other necessary parameters with less impact on cost projections, such as emission factors, are cited in the Methods section but not detailed here.

Reference for SI Part A

1 Brinckerhoff, P. Industrial Decarbonisation & Energy Efficiency Roadmaps to 2050: Iron and Steel. (2015).

2 Speizer, S. *et al.* Rapid implementation of mitigation measures can facilitate decarbonization of the global steel sector in 1.5°C-consistent pathways. *One Earth* **6**, 1494-1509, doi:10.1016/j.oneear.2023.10.016 (2023).

3 Yang, X., Nielsen, C. P., Song, S. & McElroy, M. B. Breaking the hard-to-abate bottleneck in China's path to carbon neutrality with clean hydrogen. *Nature Energy*, doi:10.1038/s41560-022-01114-6 (2022).

4 Li, J., Xie, C., Cai, W., Wang, J. & Wang, C. A Facility-Level Phaseout Strategy for China's Blast Furnaces to Address Multiple Policy Objectives. *Environmental Science & Technology* **57**, 10501-10511, doi:10.1021/acs.est.3c01289 (2023).

5 Li, Z. & Hanaoka, T. Plant-level mitigation strategies could enable carbon neutrality by 2060 and reduce non-CO2 emissions in China's iron and steel sector. *One Earth* **5**, 932-943,

doi:10.1016/j.oneear.2022.07.006 (2022).

6 Xu, R. *et al.* Plant-by-plant decarbonization strategies for the global steel industry. *Nature Climate Change* **13**, 1067-1074, doi:10.1038/s41558-023-01808-z (2023).

7 Lei, T. *et al.* Global iron and steel plant CO(2) emissions and carbon-neutrality pathways. *Nature* **622**, 514-520, doi:10.1038/s41586-023-06486-7 (2023).

8 Vogl, V., Olsson, O. & Nykvist, B. Phasing out the blast furnace to meet global climate targets. *Joule* **5**, 2646-2662, doi:10.1016/j.joule.2021.09.007 (2021).

9 Leeson, D., Mac Dowell, N., Shah, N., Petit, C. & Fennell, P. S. A Techno-economic analysis and systematic review of carbon capture and storage (CCS) applied to the iron and steel, cement, oil refining and pulp and paper industries, as well as other high purity sources. *International Journal of Greenhouse Gas Control* **61**, 71-84, doi:10.1016/j.ijggc.2017.03.020 (2017).

10 Devlin, A., Kossen, J., Goldie-Jones, H. & Yang, A. D. Global green hydrogen-based steel opportunities surrounding high quality renewable energy and iron ore deposits. *Nature Communications* **14**, doi:10.1038/s41467-023-38123-2 (2023).

11 ETC. Net-zero Steel Sector Transition Strategy. (Energy Transitions Commission, 2021).

12 Fan, Z. & Friedmann, S. J. Low-carbon production of iron and steel: Technology options, economic assessment, and policy. *Joule* **5**, 829-862, doi:10.1016/j.joule.2021.02.018 (2021).

13 WSA. *Fact sheet: biomass*, <https://worldsteel.org/publications/fact-sheets/> (2021).

14 WSA. *Fact sheet: carbon capture and storage*, <https://worldsteel.org/publications/fact-sheets/> (2021).

15 WSA. *Fact sheet: electrolysis*, <https://worldsteel.org/publications/fact-sheets/> (2021).

16 WSA. *Fact sheet: hydrogen*, <https://worldsteel.org/publications/fact-sheets/> (2021).

17 WSA. *Fact sheet: scrap*, <https://worldsteel.org/publications/fact-sheets/> (2021).

18 WSA. *Fact sheet: steel and energy use*, <https://worldsteel.org/publications/fact-sheets/> (2021).

19 WSA. *Fact sheet: steel and raw material*, <https://worldsteel.org/publications/fact-sheets/> (2021).

20 IEA. Iron and Steel Technology: Towards More Sustainable Steelmaking. (International Energy Agency, 2020).

21 Meng, J., Way, R., Verdolini, E. & Diaz Anadon, L. Comparing expert elicitation and model-based probabilistic technology cost forecasts for the energy transition. *Proc Natl Acad Sci U S A* **118**, doi:10.1073/pnas.1917165118 (2021).

22 GCCSI. Technology readiness and cost of CCS. (Global CCS Institute, 2021).

23 Grant, N., Hawkes, A., Napp, T. & Gambhir, A. Cost reductions in renewables can substantially erode the value of carbon capture and storage in mitigation pathways. *One Earth* **4**, 1588-1601, doi:10.1016/j.oneear.2021.10.024 (2021).

24 Bui, M. *et al.* Carbon capture and storage (CCS): the way forward. *Energy & Environmental Science* **11**, 1062-1176, doi:10.1039/c7ee02342a (2018).

25 Reiner, D. M. Learning through a portfolio of carbon capture and storage demonstration projects.

Nature Energy **1**, doi:Artn 15011
10.1038/Nenergy.2015.11 (2016).

26 Li, S., Zhang, X., Gao, L. & Jin, H. Learning rates and future cost curves for fossil fuel energy systems with CO2 capture: Methodology and case studies. *Applied Energy* **93**, 348-356, doi:10.1016/j.apenergy.2011.12.046 (2012).

27 Rubin, E. S., Yeh, S., Antes, M., Berkenpas, M. & Davison, J. Use of experience curves to estimate the future cost of power plants with CO2 capture. *International Journal of Greenhouse Gas Control* **1**, 188-197, doi:10.1016/S1750-5836(07)00016-3 (2007).

28 IEAGHG. Cost of CO_2 capture in the industrial sector: cement and iron and steel industries. (IEA Greenhouse Gas R&D Programme, 2018).

29 Garcia, M. & Berghout, N. Toward a common method of cost-review for carbon capture technologies in the industrial sector: cement and iron and steel plants. *International Journal of Greenhouse Gas Control* **87**, 142-158, doi:10.1016/j.ijggc.2019.05.005 (2019).

30 IEAGHG. Iron and Steel CCS Study Iron and Steel CCS Study (Techno-economics integrated steel mill). (IEA Greenhouse Gas R&D Programme, 2013).

31 IEA. CCUS in clean energy transitions. (International Energy Agency, 2020).

32 IEA. Energy Technology Perspectives 2012. (International Energy Agency, 2012).

33 IEA. Energy Technology Perspectives 2013. (International Energy Agency, 2013).

34 Arasto, A., Tsupari, E., Kärki, J., Sihvonen, M. & Lilja, J. Costs and Potential of Carbon Capture and Storage at an Integrated Steel Mill. *Energy Procedia* **37**, 7117-7124, doi:10.1016/j.egypro.2013.06.648 (2013).

35 Wiley, D. E., Ho, M. T. & Bustamante, A. Assessment of opportunities for CO2 capture at iron and steel mills: An Australian perspective. *Energy Procedia* **4**, 2654-2661, doi:10.1016/j.egypro.2011.02.165 (2011).

36 Kuramochi, T., Ramírez, A., Turkenburg, W. & Faaij, A. Comparative assessment of CO2 capture technologies for carbon-intensive industrial processes. *Progress in Energy and Combustion Science* **38**, 87-112, doi:10.1016/j.pecs.2011.05.001 (2012).

37 Tsupari, E., Kärki, J., Arasto, A. & Pisilä, E. Post-combustion capture of CO2 at an integrated steel mill – Part II: Economic feasibility. *International Journal of Greenhouse Gas Control* **16**, 278-286, doi:10.1016/j.ijggc.2012.08.017 (2013).

38 Budinis, S., Krevor, S., Dowell, N. M., Brandon, N. & Hawkes, A. An assessment of CCS costs, barriers and potential. *Energy Strategy Reviews* **22**, 61-81, doi:10.1016/j.esr.2018.08.003 (2018).

39 GCCSI. Global costs of carbon capture and storage. (Global CCS Institute, 2017).

40 Ding, H., Zheng, H., Liang, X. & Ren, L. Getting ready for carbon capture and storage in the iron and steel sector in China: Assessing the value of capture readiness. *Journal of Cleaner Production* **244**, doi:10.1016/j.jclepro.2019.118953 (2020).

41 Lee, H., Lee, J. & Koo, Y. Economic impacts of carbon capture and storage on the steel industry–A hybrid energy system model incorporating technological change. *Applied Energy* **317**, doi:10.1016/j.apenergy.2022.119208 (2022).

42 Malhotra, A. & Schmidt, T. S. Accelerating Low-Carbon Innovation. *Joule* **4**, 2259-2267,

doi:10.1016/j.joule.2020.09.004 (2020).

43 Link, S., Stephan, A., Speth, D. & Plötz, P. Rapidly declining costs of truck batteries and fuel cells enable large-scale road freight electrification. *Nature Energy*, doi:10.1038/s41560-024-01531-9 (2024).

44 Edwards, M. R. *et al.* Modeling direct air carbon capture and storage in a 1.5 °C climate future using historical analogs. *P Natl Acad Sci USA* **121**, doi:ARTN e2215679121

10.1073/pnas.2215679121 (2024).

45 IEA. CCUS Projects Database. (2024).

46 Sievert, K., Schmidt, T. S. & Steffen, B. Considering technology characteristics to project future costs of direct air capture. *Joule* **8**, doi:10.1016/j.joule.2024.02.005 (2024).

47 Morris, J. *et al.* Representing the costs of low-carbon power generation in multi-region multi-sector energy-economic models. *International Journal of Greenhouse Gas Control* **87**, 170-187, doi:10.1016/j.ijggc.2019.05.016 (2019).

48 Fajardy, M. *et al.* The economics of bioenergy with carbon capture and storage (BECCS) deployment in a 1.5 °C or 2 °C world. *Global Environmental Change* **68**, doi:10.1016/j.gloenvcha.2021.102262 (2021).

49 Mandova, H. *et al.* Achieving carbon-neutral iron and steelmaking in Europe through the deployment of bioenergy with carbon capture and storage. *Journal of Cleaner Production* **218**, 118-129, doi:10.1016/j.jclepro.2019.01.247 (2019).

50 IEA. Tracking Bioenergy with Carbon Capture and Storage. (2024).

51 IEA. Global Hydrogen Review 2023. (2023).

52 Schoots, K., Ferioli, F., Kramer, G. J. & van der Zwaan, B. C. C. Learning curves for hydrogen production technology: An assessment of observed cost reductions. *Int J Hydrogen Energ* **33**, 2630-2645, doi:10.1016/j.ijhydene.2008.03.011 (2008).

53 PwC. The green hydrogen economy: Predicting the decarbonisation agenda of tomorrow. (PwC Global, 2021).

54 IRENA. Green hydrogen cost 2020. (2020).

55 Böhm, H., Goers, S. & Zauner, A. Estimating future costs of power-to-gas – a component-based approach for technological learning. *Int J Hydrogen Energ* **44**, 30789-30805, doi:10.1016/j.ijhydene.2019.09.230 (2019).

56 Schmidt, O., Hawkes, A., Gambhir, A. & Staffell, I. The future cost of electrical energy storage based on experience rates. *Nature Energy* **2**, doi:ARTN 17110

10.1038/nenergy.2017.110 (2017).

57 Schmidt, O. *et al.* Future cost and performance of water electrolysis: An expert elicitation study. *Int J Hydrogen Energ* **42**, 30470-30492, doi:10.1016/j.ijhydene.2017.10.045 (2017).

58 Glenk, G., Holler, P. & Reichelstein, S. Advances in power-to-gas technologies: cost and conversion efficiency. *Energy & Environmental Science* **16**, 6058-6070, doi:10.1039/d3ee01208e (2023).

59 Zeyen, E., Victoria, M. & Brown, T. Endogenous learning for green hydrogen in a sector-coupled energy model for Europe. *Nature Communications* **14**, doi:10.1038/s41467-023-39397-

2 (2023).

60 Nava, V. *et al.* Plastic debris in lakes and reservoirs. *Nature* **619**, 317-322, doi:10.1038/s41586-023-06168-4 (2023).

61 Way, R., Ives, M. C., Mealy, P. & Farmer, J. D. Empirically grounded technology forecasts and the energy transition. *Joule* **6**, 2057-2082, doi:10.1016/j.joule.2022.08.009 (2022).

62 IRENA. Global Hydrogen Trade Costs 2022. (2022).

63 Odenweller, A., Ueckerdt, F., Nemet, G. F., Jensterle, M. & Luderer, G. Probabilistic feasibility space of scaling up green hydrogen supply. *Nature Energy* **7**, 854-865, doi:10.1038/s41560-022-01097-4 (2022).

64 IEA. Hydrogen Production Projects Database. (2024).

65 IEA. Net Zero Roadmap: A Global Pathway to Keep the 1.5 °C Goal in Reach. (2023).

66 Yang, B., Zhang, R., Shao, Z. & Zhang, C. The economic analysis for hydrogen production cost towards electrolyzer technologies: Current and future competitiveness. *Int J Hydrogen Energ* **48**, 13767-13779, doi:10.1016/j.ijhydene.2022.12.204 (2023).

67 Nemet, G., Greene, J., Müller-Hansen, F. & Minx, J. C. Dataset on the adoption of historical technologies informs the scale-up of emerging carbon dioxide removal measures. *Communications Earth & Environment* **4**, doi:ARTN 397

10.1038/s43247-023-01056-1 (2023).

68 Saltelli, A., Chan, K. & Scott, E. M. *Sensitivity analysis: Gauging the worth of scientific models*. (John Wiley & Sons, 2000).

69 Lempert, R. *et al.* Ensuring robust flood risk management in Ho Chi Minh City. *World Bank Policy Research Working Paper* (2013).

70 IRENA. Renewable power generation costs in 2022. (International Renewable Energy Agency, 2022).

71 WSA. Sustainability Indicator 2023 Report. (World Steel Association, 2023).

Part B - Systematic review of CCS initial cost and learning rate

Note S9. PRISMA of CCS initial cost and learning rate.

9.1 Initial cost of CCS.

9.1.1 Background and motivation.

CCS plays a crucial role in the decarbonization of the iron and steel sector and is compatible with multiple production routes, including BF-BOF, DRI-EAF, SR-BOF, and DRI-BOF (Extended Data Table 1). Our component-based cost forecast method divides the cost of decarbonization technologies involving CCS into processing route costs and CCS costs. The former is estimated from our plant-level production and cost databases, while the latter is derived from Wright's learning curve (see details in Methodology section in the main text and Note S3 in Supplementary Information). To estimate the learning curve for CCS, three input parameters are required: initial cost, learning rate, and cumulative capacity. The first two parameters were determined through two separate systematic literature reviews due to the absence of historical actual data, and the cumulative capacity was based on the IEA CCS project databases (see in Note S4 in Supplementary Information). This section details our literature search, screening, selection, and data extraction processes, following PRISMA guidelines where applicable, to enhance the transparency and reproducibility of our literature analysis.

9.1.2 Research question and objectives.

We conducted a systematic review to determine the initial capture cost of CCS in steel plants. Notably, due to significant variations in CCS costs across different sectors, influenced by varying CO_2 emission concentrations and patterns, we specifically focused our literature search on CCS applications in the iron and steel industry. Additionally, during the literature screening process, any reporting format of CO_2 capture costs (e.g., cost of CO_2 capture vs. cost of CO_2 avoidance) are acceptable, despite their different meanings and values. We will address these differences further during the data extraction process.

9.1.3 Search strategy.

To begin with, a comprehensive literature search was conducted using the Web of Science and Scopus databases due to their extensive coverage of high-quality, peer-reviewed journals. The search strategies outlined in Table S9 were applied to article titles, abstracts, and keywords. We also manually screened the reference lists of included studies to ensure no significant articles were missed. Additionally, grey literature from major industry and climate organizations, such as the International Energy Agency (IEA), Global CCS Institute (GCCSI), and International Energy Agency Greenhouse Gas R&D Programme (IEAGHG), was manually included to complement the academic literature. To qualify for inclusion, grey literature reports needed to satisfy two criteria: they must have been cited in academic studies and contain

the required quantitative data.

Table S9 Search strategy in the Web of Science and Scopus databases.

Objectives	Keywords	Field of research	Period
Initial CCS capture cost	(“carbon capture” OR CCS OR “CO2 capture” OR CCUS) AND (cost OR techno-economic*) AND (steel*) NOT (mineral*) NOT ("hydrogen production")	Title, Abstract, Keywords	Start date-2024
Learning rate of CCS capture cost	(“carbon capture” OR CCS OR “CO2 capture” OR CCUS) AND ("learning curve*" OR "experience curve*" OR "techn* learning" OR "cost reduction" OR "future cost" OR "learning rate*"OR "experience rate*")	Title, Abstract, Keywords	Start date-2024

9.1.4 Literature screening, inclusion and exclusion criteria.

As illustrated in Fig. S30, 1088 records were initially retrieved from the Web of Science and Scopus databases using the search strategy outlined in Table S9. We removed 247 duplicates and 97 records that were categorized as patents, news, and editorial materials, leaving 744 articles, conference papers, thesis, or book chapters for further screening.

We then screened the titles and abstracts of each record, excluding 628 studies that were not relevant to our research question. This involved studies from unrelated fields such as mathematics, physics, chemistry, agronomy, medicine, and materials science, as well as those lacking at least one of the three key elements of our study: CCS technology, the steel sector, and cost analysis. Furthermore, 47 studies focusing on costs of CO_2 storage and transportation, and 8 studies on cost optimization of supply chain for CCS in steel plants were also excluded because they did not focus on capture or avoidance costs of CCS.

After abstract screening, 61 articles remained for full-text eligibility assessment. One could not be retrieved, and the remaining 60 were reviewed in full. During the review, key characteristics of each study were documented, including data sources, methods, and technical, economic, and regional assumptions, as detailed in the *Data Extraction and Processing* section. These characteristics are crucial as they significantly impact the estimated CCS costs in different studies. By systematically comparing these attributes, we refined the inclusion criteria and identified studies that aligned with the requirements outlined in Table S10. This process ensured the inclusion of studies with reliable data sources and methods, fostering consistency in research subject, scope, and assumptions, thereby enhancing the quality and comparability of the cited literature values in this paper.

As a result, 27 full-text articles were excluded for lacking original and reliable estimates of initial CCS costs. Specifically, those studies: a) did not provide cost values, b) were

reviews or relied on previous studies, or c) lacked detailed data sources or calculation methods. Since our focus was on extracting initial costs rather than future projections, studies like Leeson et al. (2017), which projected future costs of CCS based on a review of existing initial cost estimates, were also excluded. To further improve comparability, an additional 21 full-text articles were excluded due to assumptions inconsistent with most other literature. Those studies: d) considered only capital cost while omitting operating costs, e) focused on capture technologies other than MEA, f) reported cost of CO_2 capture instead of cost of CO_2 avoided (see Table S11).

Ultimately, only 12 of the 60 reviewed full-text academic articles were included. In addition, we identified 6 institutional reports from climate-related organizations through reference checking. Of these, 2 were excluded based on criterion f) for not reporting CO_2 avoidance costs. Therefore, a total of 16 academic articles and institutional reports were finally included for further review.

All screened references are detailed in the PRISMA Literature Dataset, available as supplementary material (see Data Availability section in the main text).

Table S10 Eligibility criteria for full-text assessment.

Inclusion Criteria	Exclusion Criteria
- Articles, conference papers, review papers, thesis, or book chapter	- Other document types (e.g., patents, news, editorial materials)
- Focus on CCS initial costs for steel plants	- Non-steel sectors or missing cost values
- Capture cost explicitly provided	- Focus only on transport, storage, or supply chain costs
- Provision of original CCS cost estimates	- Lack of original CCS cost estimates
- Clear data sources and methodologies	- Unclear data sources or methodologies
- Both capital and operating costs analyzed	- Studies considering only capital cost
- Commonly studied capture technology (e.g., MEA-based CCS systems)	- Focus on other advanced capture technologies
- Costs expressed as per ton of CO_2 avoided	- Costs expressed as per ton of CO_2 captured

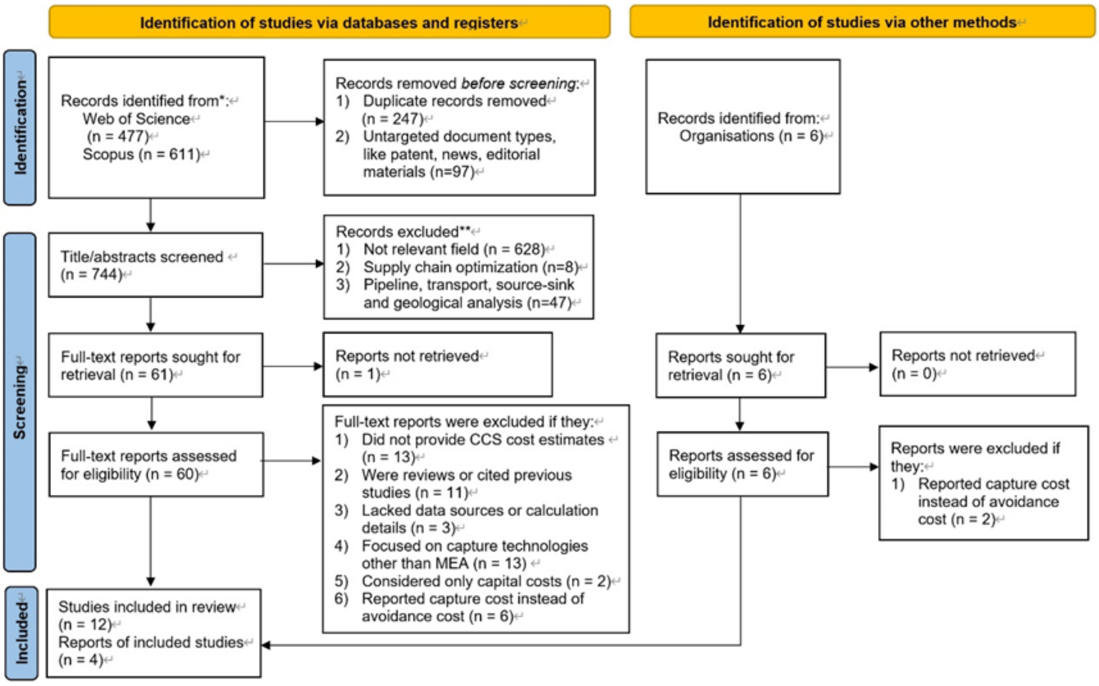

Fig. S30 Literature search procedures PRISMA flow diagram illustrates the procedures (i.e., identification, screening, eligibility and inclusion) for obtaining literature values for CCS initial capture costs in steel plants.

9.1.5 Data extraction and processing.

Literature characteristics and selection. Multiple factors significantly influence the initial CCS costs reported across studies, including the capture technologies employed, the methodologies applied for cost estimation, the choice of cost metrics, the scope of cost components quantified, and the geographic context[1]. During the full-text eligibility assessment, we initially identified 33 studies that provided original CCS capture cost estimates with detailed data sources and methodologies, of which 16 were included and 17 were excluded. Considerable variations in their technical, economic, and regional assumptions raised concerns about the comparability of their results. To address these issues, ensure consistency in assumptions, and improve the comparability of cited cost estimates, we systematically extracted key characteristics from the literature alongside the reported cost estimates. These characteristics included authorship, study type (academic article or organizational report), capture technologies, estimation methods, cost metrics, and specific geographic focus, as summarized in Table S11.

The CCS capture technologies evaluated in these studies include MEA, VPSA, MDEA, calcium looping, SEWGS, PEM, and membrane, each characterized by distinct technical and economic differences. Among these, MEA stands out for its technical maturity and has been extensively investigated by 21 out of 33 studies; therefore, we used the reported cost of this technology as an inclusion criterion for further review. Two metrics are commonly used in the literature to report CCS capture costs: the cost

of CO_2 capture and the cost of CO_2 avoidance. The former represents the direct expenses of capturing CO_2, including separation, compression, and sometimes transportation, while the latter measures the incremental cost of reducing CO_2 emissions compared to a baseline without CCS, factoring in changes in energy use, efficiency, and associated emissions[2]. Although these two metrics are interrelated, converting between them requires additional parameters that are often not disclosed in the literature. To avoid introducing uncertainties during this conversion process and to ensure the comparability of cited values, we included only studies reporting the cost of CO_2 avoidance. This metric is not only better suited for comparing technologies, aligning with the focus of our study, but is also more widely reported, with 16 of the 21 MEA studies in Table S11 providing this data. Additionally, all 16 included studies accounted for both capital and operational costs, providing a comprehensive view of the cost components of CCS capture. In contrast, studies that considered only capital cost were excluded in earlier steps and are therefore not represented in the literature characterization table.

This review applied rigorous inclusion and exclusion criteria to select studies with transparent data, reliable methodologies, and clearly articulated assumptions, aiming to minimize the risk of bias in the included research. However, due to the lack of commercial-scale CCS applications and data in steel plants, most studies rely heavily on model-based assumptions, potentially introducing systematic errors. Consequently, the cost estimates reported in the literature may deviate from future real-world values, a discrepancy that remains challenging to quantify until empirical data becomes available. To address this, we performed a sensitivity analysis on the initial CCS cost projections to assess the potential impacts of systematic errors and enhance the robustness of our results as much as possible (see the *Data selection and uncertainty* section below).

Table S11 Key literature characteristics on CCS costs in steel production.

No.	Author	Literature type	Capture type	Method	Cost type	Region	Comment
1	GCCSI (2017)	Organization report	MEA	Case study	Avoid cost	14 regions	Included
2	IEAGHG (2018)	Organization report	Multiple	Modeling study	Avoid cost	North-western EU	Included
3	IEAGHG (2013)	Organization report	MEA	Case study	Avoid cost	Netherlands	Included
4	IEA (2013)	Organization report	MEA	Modeling study	Avoid cost	United States	Included
5	Arasto et al. (2013)	Academic article	MEA	Case study	Avoid cost	Finland	Included
6	Biermann et al. (2019)	Academic article	MEA	Case study	Avoid cost	Sweden	Included
7	Ding et al. (2020)	Academic article	MEA	Case study	Avoid cost	China	Included
8	Ho et al. (2011)	Academic article	MEA	Case study	Avoid cost	Australia	Included
9	Kuramochi et al. (2011)	Academic article	MEA	Case study	Avoid cost	Netherlands	Included
10	Kuramochi et al. (2012)	Academic article	MEA	Data reprocessing	Avoid cost	Not specify	Included
11	Liang et al. (2019)	Academic article	MEA	Case study	Avoid cost	China	Included
12	Tsupari et al. (2013)	Academic article	MEA	Case study	Avoid cost	Finland	Included
13	Wiley et al. (2011)	Academic article	MEA	Case study	Avoid cost	Australia	Included
14	Ho et al. (2013)	Academic article	MEA、VPSA	Case study	Avoid cost	Australia	Included
15	Zang et al. (2023)	Academic article	MEA	Modeling	Avoid cost	USA	Included
16	Hooey et al. (2013)	Academic article	MEA	Modeling	Avoid cost	South Europe	Included
17	IEA (2020)	Organization report	Not specigy	Modeling	Capture cost	Global	Excluded
18	GCCSI (2021)	Organization report	Not specify	Modeling	Capture cost	Not specify	Excluded
19	Leeson et al. (2017)	Academic article	MEA、VPSA	Data reprocessing	Capture cost	Not specify	Excluded
20	Johnsson et al. (2020)	Academic article	MEA	Case study	Capture cost	Sweden	Excluded
21	Yang et al. (2023)	Academic article	MEA	Case study	Capture cost	China	Excluded
22	Skagestad et al. (2014)	Academic article	MEA	Case study	Capture cost	North Europe	Excluded
23	Bains et al. (2017)	Academic article	MEA	Modeling	Capture cost	USA	Excluded
24	Tsupari et al. (2015)	Academic article	VSPA	Case study	Avoid cost	Finland	Excluded
25	Cormos et al. (2020)	Academic article	Calcium looping	Case study	Avoid cost	-	Excluded
26	Zecca et al. (2023)	Academic article	SEWGS	Case study	Avoid cost	-	Excluded
27	Manzolini et al. (2020)	Academic article	SEWGS	Case study	Avoid cost	-	Excluded
28	Perpinan et al. (2023)	Academic article	PEM	Modeling	-	-	Excluded
29	Yun et al. (2021)	Academic article	Membrane	Modeling	-	-	Excluded
30	Jeong et al. (2023)	Academic article	FINEX	Modeling	-	-	Excluded
31	Chung et al. (2018)	Academic article	Membrane	Modeling	-	-	Excluded
32	Lie et al. (2007)	Academic article	Membrane	Modeling	-	-	Excluded
33	Khallaghi et al. (2022)	Academic article	MDEA	Modeling	-	-	Excluded

Note: a) The 33 studies comprise 16 that were included after a full-text eligibility assessment and 17 that, although providing original and reliable cost estimates, were excluded due to their differences in technical and economic assumptions compared to most studies. This exclusion aims to enhance the comparability of the included literature. b) The cost types "avoid cost" and "capture cost" in this table refer to the cost metrics discussed above: the cost of CO_2 avoidance and the cost of CO_2 capture, respectively. c) The data reprocessing method involves the harmonization and standardization of values extracted from literature. d) More reference information is provided in the PRISMA Literature Dataset (see Data Availability section in the main text).

Data extraction. We extracted 30 estimates of CO_2 avoidance costs for CCS using MEA technology in steel production from 16 eligible studies. For each estimate, we also collected literature details, including authorship, publication year, year of the cost estimate, currency year, research method, and regional context. To enable cross-regional and temporal comparisons, all cost values originally reported in various currencies and currency years were standardized to 2021 USD using historical exchange rates and the Chemical Engineering Plant Cost Index (CEPCI), following the approach of Leeson et al. (2017).

As shown in Fig. S31, although costs generally decline over time, notable differences persist even among studies conducted in the same region, by the same authors, or within

the same year. Despite focusing on the same technology and cost metric, the included studies show substantial variability in data sources, methods, and geographic scopes. Based on methods and data types, these studies are categorized into three groups: 11 case studies that conduct techno-economic assessments for specific plants, 4 regional modeling studies that use generalized data and assumptions to estimate costs at national or regional scales, and 1 literature review that harmonizes previous estimates by standardizing assumptions, currencies, and reference years. Geographically, most studies are confined to a single plant or country, including the Netherlands, Finland, Sweden, Australia, China, and the United States. Only a few address cross-regional comparisons, such as IEAGHG (2018) examining multiple countries in Northwest Europe, Hooey et al. (2013) evaluating several Southern European nations, and GCCSI (2017) comparing CCS costs across 14 global regions and countries.

To illustrate the cost variability, Fig. S32 presents descriptive statistics, boxplots, and subgroup comparisons of the collected literature values. The mean and median reflect central tendency, with the latter being less sensitive to outliers. Dispersion is measured using the interquartile range (IQR) and standard deviation (SD). The relative standard deviation (RSD), calculated as SD divided by the mean, allows comparison across groups with different average costs. For all 30 extracted estimates, the overall mean was \$92/tCO_2, with a SD of \$22.6/tCO_2, corresponding to an RSD of approximately 25%. Fig. S32 also displays cost distributions by subgroup, categorized by methodological approach, study type, and region, with RSDs for these subgroups ranging from 11% to 37%.

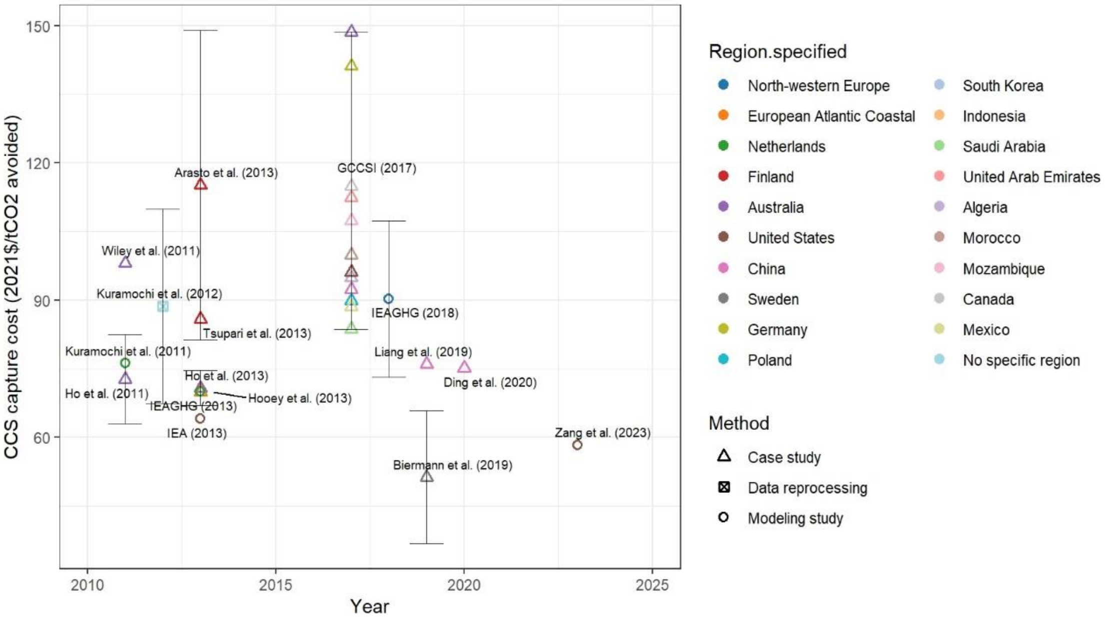

Fig. S31 Literature values on initial CCS costs in steel production. Cost estimates were extracted from 16 key studies identified through full-text screening based on the PRISMA framework. The year shows the year of each estimate; if not reported, the publication year was used as a substitute. Original cost values were standardized to 2021 USD using historical

exchange rates and the Chemical Engineering Plant Cost Index (CEPCI), following the approach of Leeson et al. (2017). Each point represents the mean value reported in a study, with bars indicating the range between minimum and maximum values. Colors indicate geographic regions, and point shapes denote the cost estimation methods. Additional reference details are provided in the PRISMA Literature Dataset (see Data Availability section in the main text).

Data selection and uncertainty. This systematic review aimed to identify initial CCS cost estimates for steelmaking technologies, serving as the input parameter for a learning curve model projecting decarbonization costs in global steel plants. Rather than synthesizing a single pooled estimate across all studies, we screened studies based on methodological compatibility, sectoral specificity, and cost metric alignment, to derive internally consistent parameter ranges for our modeling. Moreover, to ensure compatibility with our global-scope, plant-level granularity framework, we prioritized literature estimates that reflect regional variations.

Among all extracted data, 14 region-specific cost estimates from GCCSI (2017) stood out for their uniform methodology, consistent technical-economic assumptions, and broad geographic coverage, ensuring the regional cost differences internally coherent and reliably comparable. These values were used as region-level initial cost inputs for our learning curve model, generating results shown in the main text. For regions not covered by literature, missing values were imputed using averages from geographically proximate regions.

To account for uncertainty, we defined the range bounds as the mean ± two standard deviations (±2 SD) of all 30 extracted cost estimates, thereby capturing the total observed variability across methodological, technical, economic, and geographical assumptions. We then performed two sets of sensitivity tests (i.e., a marginal effect type and an LHS method-based joint test) based on this broad range, examining the impact of initial CCS cost on technology cost projections. As detailed in Notes S7, the results demonstrate that this level of uncertainty has a limited effect on the projected costs of 20 low-carbon technologies, as well as on the optimal decarbonization pathways, emission reduction potential, and abatement costs, thereby underscoring the robustness of our projections even in the presence of input variability.

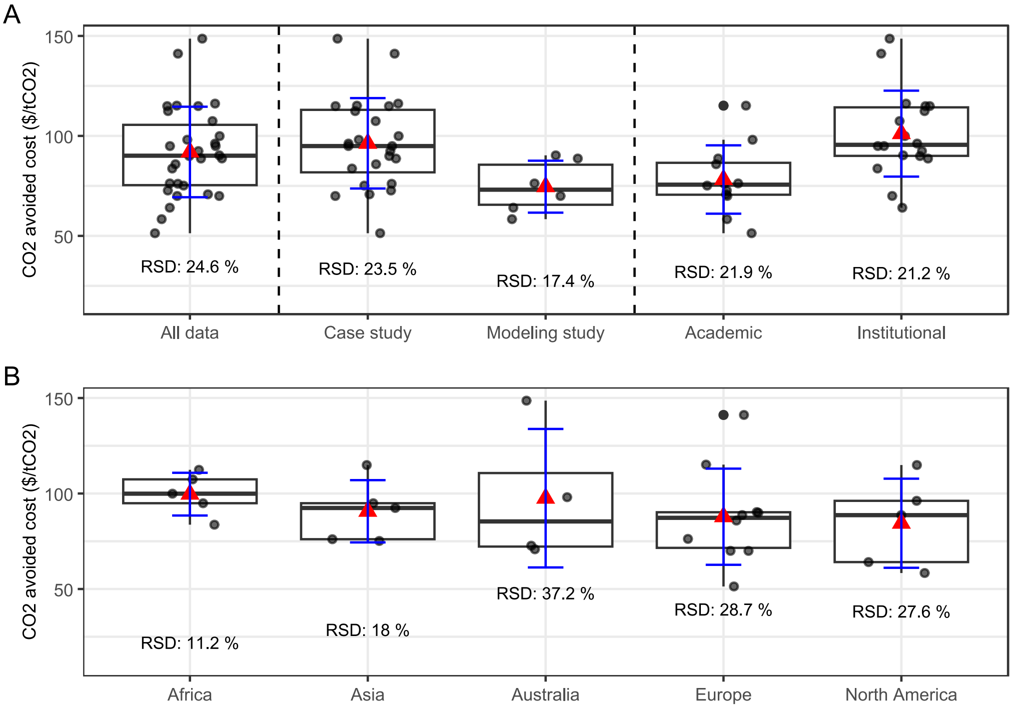

Fig. S32 Boxplot and summary statistics of literature values for initial CCS costs. Each column represents literature data grouped by specific characteristics: A) from left to right, columns display all data from the 16 included studies, data from case studies or modeling studies, and data from academic papers or organization reports; B) columns show data specific to regions, including Africa, Asia, Australia, Europe, and North America. Grey points indicate standardized cost values, red triangles denote means, and blue bars show standard deviations (SD) to represent data dispersion. The boxplot displays the first quartile (Q1), median (Q2), and third quartile (Q3). Black whiskers extend to 1.5 times the interquartile range (IQR) above and below the box, with data points beyond this range considered outliers. Relative standard deviations (RSD) are also presented for each group to facilitate comparisons of data dispersion across groups with very different means. This plot excludes cost data from Kuramochi et al. (2012), as it is derived from reprocessed literature data without a specified region.

9.2 Learning rate of CCS.

9.2.1 Background and motivation.

The costs of emerging technologies typically decrease with accumulated experience and advancements in technical performance, a phenomenon known as the technology learning effect. This effect is modeled in existing studies either by exogenously specifying future cost trends or by employing learning curves that link future costs to key drivers such as experience, research and development spending, and investments. Among these models, Wright's law is the most widely used for projecting the costs of novel technologies with limited historical data. In Wright's law, the learning rate serves as a critical parameter, quantifying the percentage cost reduction with each doubling of cumulative production. However, unlike well-studied and commercialized technologies such as renewable energy and batteries, the learning rate for CCS remains fragmented and inconsistent across the literature. To support robust projections of CCS cost, a thorough review and synthesis of appropriate CCS learning rates from existing research is essential.

9.2.2 Research question and objectives.

This systematic review aims to identify the learning rate of CCS for use in Wright's law learning curve model. Despite our primary focus on iron and steel plants, we extended the scope to review CCS learning rates across various industries without sectoral limitations. This broader focus was prompted by our finding that few studies in the steel sector addressed learning rates, and those that did typically referenced CCS applications in the power sector.

9.2.3 Search strategies.

A comprehensive literature search on CCS learning rates was conducted in the Web of Science and Scopus databases using the search strategies detailed in Table S9, without sectoral restrictions. The search targeted titles, abstracts, and keywords, encompassing studies published from the earliest available year to 2024. To ensure no significant studies were missed, we manually screened the reference lists of the included articles. Additionally, grey literature from relevant organizations, such as the International Energy Agency (IEA), the Global CCS Institute (GCCSI), and the International Energy Agency Greenhouse Gas R&D Programme (IEAGHG), was included to complement the academic literature, provided these sources had been cited in scholarly studies.

9.2.4 Literature screening, inclusion and exclusion criteria.

Literature identification. As shown in Fig. S33, an initial total of 1883 records were retrieved from the Web of Science and Scopus databases. Of these, 308 records were removed due to the following reasons:

- Duplicates (n = 270), or
- Untargeted document types, such as patents, news, and editorial materials (n = 38).

Title and Abstract Screening. Two researchers independently screened the titles and abstracts of the remaining 1575 studies, applying the predetermined inclusion and exclusion criteria outlined in Table S12. This process resulted in the exclusion of 1500 records unrelated to CCS capture and learning effects, as follows:

- 1129 records were from unrelated research areas (e.g., mathematics, physics, chemistry, agriculture, medicine, biology, materials science, education, and sociology),
- 177 records focused on other technologies, such as renewable energy and hydrogen production, rather than CCS,
- 106 records concentrated on CO_2 transport and storage or on socio-environmental and policy assessments of CCS, rather than on capture processes and costs,
- 88 records addressed only technical and static cost assessments (TEA) of CCS capture without examining technology learning effects.

Full-text Screening. After the abstract screening, 75 records remained for full-text eligibility assessment based on their mention of CCS and learning effects, with the potential to include learning rate estimates for CCS. During this phase, additional exclusion criteria were applied, resulting in the removal of:

- 11 records that focused on technical or environmental learning effects rather than economic learning effects,
- 16 records that employed assumed cost reductions over time, endogenous learning curves, learning-by-researching (i.e., cost reductions driven by R&D), or non-linear optimization methods; only one-factor experience curves consistent with Wright's law were included,
- 4 records that provided learning rates only for capital and O&M costs at the component level, rather than for total costs,
- 14 records that did not report learning rate values or lacked transparent sources and calculation methods,
- 22 records that cited CCS learning rates from previous studies without presenting original estimates.

As a result, only 8 academic articles were included. Additionally, eight studies from authoritative organizations, such as the International Energy Agency (IEA), the Global CCS Institute (GCCSI), and the International Energy Agency Greenhouse Gas R&D Programme (IEAGHG), along with other related projects, were manually identified through reference checking. Of these, seven studies were excluded because they either reported learning rates only for component costs of CCS, reviewed energy technologies unrelated to CCS, or lacked clear data sources and methodological details. Only one studies was included to supplement academic literature. In total, 9 studies including

academic articles and organizational reports were selected for further review and data extraction.

All screened references are detailed in the PRISMA Literature Dataset, available as supplementary material (see Data Availability section in the main text).

Table S12 Eligibility Criteria for Full-Text Assessment.

Inclusion Criteria	Exclusion Criteria
- Articles, conference papers, reviews, thesis, or book chapters	- Other document types (e.g., patents, news, editorial materials)
- Focus on CCS capture process	- Focus on other technologies
- Dynamic costs of CCS capture	- Static cost assessments without learning effects
- Investigation of learning effects on economic costs	- Advancements in technical performance or environmental effects
- Utilization of learning curves	- Assumption of cost reductions over time
- Use of experience curves based on Wright's law	- Use of endogenous or dual-factor learning curves (e.g., involving R&D) or optimization methods
- Provision of learning rate estimates	- Lack of learning rate values or unclear sources
- Learning rates for total costs	- Learning rates for capital or O&M costs only
- Original estimation of learning rates	- Secondary citations of learning rates

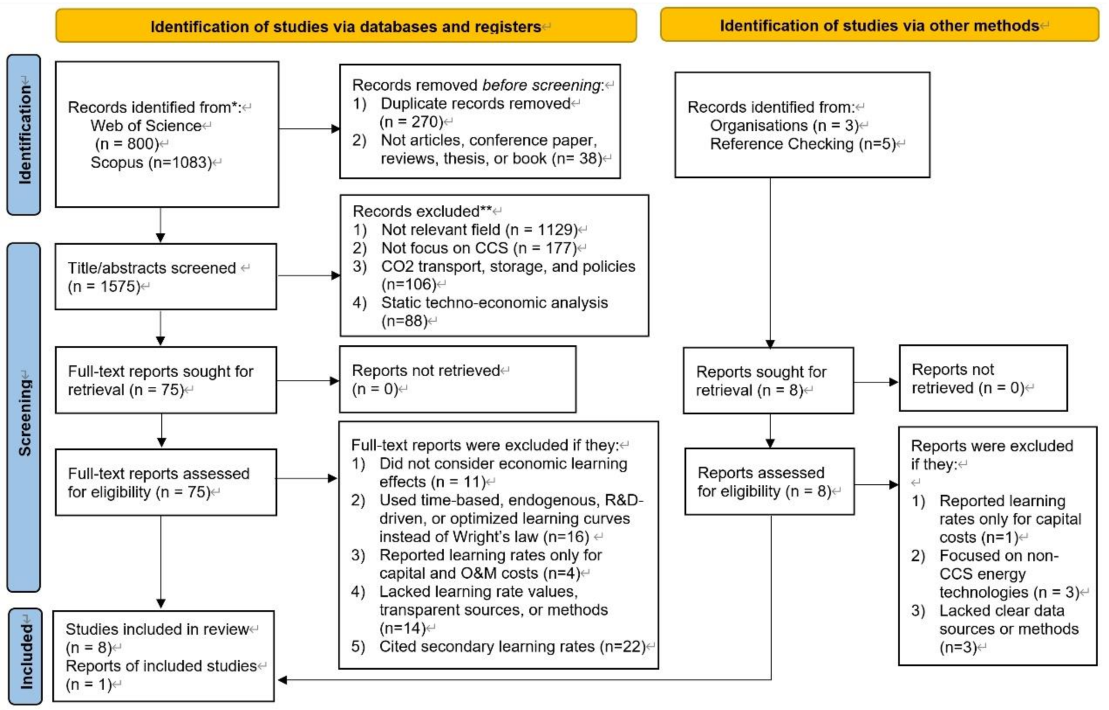

Fig. S33 Literature search procedures. PRISMA flow diagram illustrates the procedures (i.e., identification, screening, eligibility and inclusion) for obtaining literature values for the learning rate of CCS capture cost.

9.2.5 Data extraction and processing.

Literature characteristics. Our systematic review identified nine studies that contain

original, quantitative learning-rate estimates for CCS (Table S13). These studies span 2004-2023 and differ in estimation methods, technical assumptions, cost metrics, data sources, and sectoral scope. To contextualize these results, we also examined 18 frequently cited but ultimately excluded studies that either cited previous estimates without recalculation, lacked clear data sources, or reported learning rates for cost metrics inconsistent with those defined in our study. Although excluded during the literature screening, these studies provided useful context for interpreting the reported learning rates.

Drawing on existing studies, learning rate estimation methods can be classified into five categories[3-6]: a) empirical curves fitting based on historical cost and capacity trends, b) technology analogies (e.g., SO_2/NO_x capture systems), c) component-based approaches that aggregate individual component learning rates, d) expert elicitation, and e) hybrid models combining top-down learning curves with bottom-up engineering-economic analyses. Unlike mature technologies such as solar PV and batteries, limited commercial deployment of CCS and scarce historical cost data constrain the applicability of empirical learning curves[7]. Early studies therefore treated CO_2 capture as technically analogous to SO_2/NO_x capture in coal-fired power plants and inferred learning rates for CCS capital cost accordingly (e.g. Riahi et al., 2004; Rubin et al., 2004). IEAGHG (2006) was the first to apply a component-based approach, providing separate learning rates for capital and operating & maintenance (O&M) costs in CCS power plants. Building on this method and its estimates, subsequent research, including Rubin et al. (2007), van den Broek et al. (2009), and Li et al. (2014), projected learning rates for electricity generation costs from CCS-equipped plants, while Li et al. (2012a), Zhou et al. (2018), and Kang et al. (2020) reported learning rates for CO_2 avoidance costs across various types of power plants. In contrast, studies such as Liu et al. (2022) and van de Spek et al. (2017) applied the same approach to project electricity cost reductions without explicitly estimating learning rates.

More recent research has expanded sectoral coverage beyond power generation. In the industrial sector, such as steel and chemical production, studies have employed expert elicitation (Ding et al., 2020), transferred learning rates from the power sector (Leeson et al., 2017), or drawn analogies with other low-carbon energy technologies (Moglaensi et al., 2023; Rutinger et al., 2021). Several influential institutional reports (e.g., IEA, 2020; GCCSI, 2019; NETL, 2013) also reference CCS learning rates in their multi-sector analyses but lack traceable data sources.

Table S13 Key characteristics of literature on CCS learning rates.

No	Author	Original estimates	Methods	Data source	Learning rates (LR)	Research outcomes/ Use case	Comments
1	Rubin et al. (2007)	Yes	Component-based learning curves	IEAGHG (2006), Rubin et al. (2007), van den Broek (2009)	2.1-5.0% (1.1-7.6%)	LR for electricity generation cost	Included
2	van den Broek et al. (2009)	Yes	Component-based learning curves		5% (2-7%)	LR for electricity generation cost	Included
3	Li et al. (2012a)	Yes	Component-based learning curves		7.6-14.2% (5.7-14.3%)	LR for electricity generation cost	Included
4	Li et al. (2014)	Yes	Component-based learning curves		7.6-10.8% (5.7-14.3%)	LR for electricity generation cost	Included
5	Wu et al. (2016)	Yes	Component-based learning curves		5.3-10.2% (3.6-10.2%)	LR for electricity generation cost	Included
6	Li et al. (2012b)	Yes	Component-based learning curves	van den Broek (2009)	10.34% (6.4-14.6%)	LR for CO2 avoidance cost in power plants	Included
7	Kang et al. (2020)	Yes	Component-based learning curves	van den Broek (2010)	5.6-10.2% (1.8-15.1%)	LR for CO2 avoidance cost in power plants	Included
8	Zhou et al. (2018)	Yes	Component-based learning curves	van den Broek (2011)	7.3% (3.4-13.3%)	LR for CO2 avoidance cost in coal-to-liquid	Included
9	Ding et al. (2020)	Yes	Expert elicitation	Elicitation of insights from 10 experts	8%	LR for CCS in steel plants	Included
10	Riahi et al. (2004a)	No	Analogy to SO2 capture	Empirical learning rates of capital cost of SO2 captue	13% for capital cost	LR for CCS capital cost in power plants	Excluded
11	Riahi et al. (2004b)	No					Excluded
12	Rubin et al. (2004)	No	Analogy to SO2 and NOx capture	Empirical learning rates of capital cost of SO2 and NOx capture	11-12% for capital cost	LR for CCS capital cost in power plants	Excluded
13	IEAGHG (2006)	No	Analogy to SO2 capture	Empirical learning rates of capital and O&M costs of SO2	12% (6-18%) for capital cost; 22% (10-30%) for O&M costs	LR for CCS components in power plants	Excluded
14	Rochedo et al. (2013)	No	Engineering economic model and learning curves	Optimal CCS technical performance	12% for capital cost; 23% for O&M costs	LR for optimal CCS performance in power plants	Excluded
15	Wendling et al. (2019)	No	Referenced CCS literature	IEAGHG (2006), Rubin et al. (2007), van den Broek (2009)	Same with IEAGHG (2006)	Only electricity cost projections	Excluded
16	Zhai et al. (2019)	No				Only electricity cost projections	Excluded
17	Azarabadi et al. (2020)	No				Only electricity cost projections	Excluded
18	Liu et al. (2022)	No				Only CO2-to-chemical cost projections	Excluded
19	Leeson et al. (2017)	No	Referenced CCS and energy-related technologies	Rubin et al. (2007)	3.5%	Industrial decarbonization cost, including steel, cement, oil, and paper	Excluded
20	Lee et al. (2022)	No		Ding et al. (2020)	10%	Steel decarbonization cost	Excluded
21	Mogliaensi et al. (2023)	No		Boham et al. (2018)	10.4% (5-15.8%)	Steel decarbonization cost	Excluded
22	van der Spek et al. (2017)	No		Rubin et al. (2004), Mcdonald & Schrattenholzer (2001)	11% (10-14%)	Only electricity cost projections	Excluded
23	Fan et al. (2018)	No				Only electricity cost projections	Excluded
24	Rutinger et al. (2021)	No		Zauner et al. (2019)	11%	Transport decarbonization cost	Excluded
25	NETL (2013)	No	Unknown	Unknown	3%	Electricity generation cost	Excluded
26	GCCSI (2019)	No	Unknown	Unknown	12%	CCS cost trends in all sectors	Excluded
27	IEA (2020)	No	Unknown	Unknown	12%	CCS application potential in all sectors	Excluded

Note: a) The 27 studies consist of 9 that passed the full-text eligibility assessment (colored in brown) and 18 that, although excluded, are presented here for comparison (no color). b) Unless stated otherwise, the learning rate (LR) represents the rate of cost reduction in total cost, including both capital and operating & maintenance (O&M) costs. c) Data source indicates the origin of CCS learning rate used in the literature. d) More reference information is provided in the PRISMA Literature Dataset (see Data Availability section in the main text).

Data extraction. From the eligible and representative studies listed in Table S13, we extracted 38 learning rate estimates, along with key contextual information such as estimation methods, data sources, cost metrics, and sectoral scopes. As illustrated in Fig. S34, the original learning rate estimates were categorized into five groups based on their underlying methodology: analogies to SO_2 and NO_x capture technologies (11-13%)[8-10], expert elicitation (8%)[11], hybrid models combining learning curves with engineering optimization (12%)[12], and component-based learning curves applied to either electricity generation costs (2-14%)[13-15] or CO_2 avoidance costs (6-10%)[16,17,18]. Secondary learning rate estimates were classified according to their data sources: either referencing previous CCS or energy technology learning rates (3.5-12%)[19-21], or lacking

a clearly specified source (3-12%)[22-24].

Fig. S35B presents descriptive statistics of all extracted data, grouped by methodological approach. Learning rates derived from hybrid models and analogies to SO_2 and NO_x capture technologies are slightly higher than those obtained from expert elicitation and component-based learning curves for CO_2 avoidance and electricity costs. This difference likely arises because the former report learning rates only for capital or O&M costs, while the latter are based on total costs. Since our review requires learning rates corresponding to total initial CCS costs rather than capital or O&M costs alone, estimates based on hybrid models and SO_2/NO_x analogies were excluded, despite their foundational role and frequent citation. Estimates cited from previous studies or lacking clear sources were also excluded (see Table S12). Consequently, 20 original learning rate estimates were retained, yielding an average of 7% and a standard deviation of 3% (as shown in Fig. S35A).

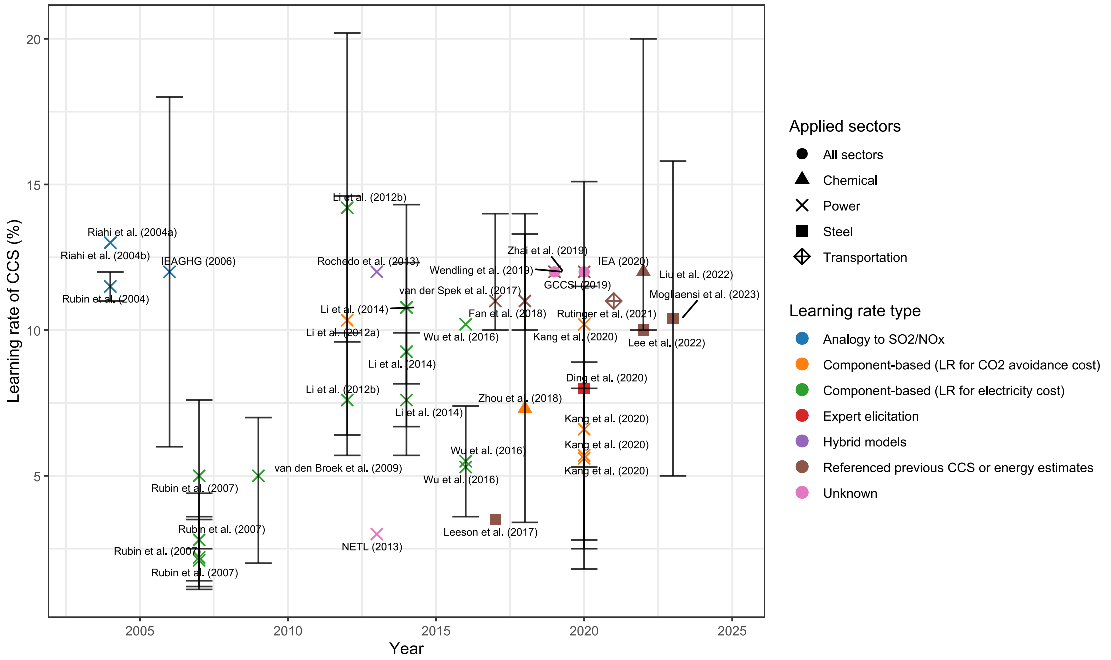

Fig. S34 Literature-derived learning rates for CCS. Learning rate estimates were extracted from 27 key studies identified through literature screening following the PRISMA framework. These include 20 original CCS learning rate estimates from 9 included studies, along with 18 estimates from 18 representative excluded studies for comparison. The points represent the primary results reported in each study, while the bars indicate the minimum and maximum values provided in the corresponding literature. Colors signify key characteristics and factors influencing the estimates, such as methods, metrics, and analogous technologies, and the shapes of the points represent the sectors to which the CCS learning rates were applied. More reference information is provided in the PRISMA Literature Dataset (see Data Availability section in the main text).

Data selection and uncertainty. To identify appropriate literature values for our learning curve model, we applied strict exclusion criteria and selected studies whose assumptions aligned with our modeling needs. Although we prioritized evidence from the steel industry, only one study provided an original learning rate estimate for this sector, prompting us to consider studies from other sectors as well. In terms of cost metrics, we favored learning rates based on total cost rather than capital or O&M costs alone, and therefore included learning rates for the total cost of either CO_2 avoidance or electricity generation with CCS. The former aligns more directly with our initial cost units (measured in terms of CO_2 avoided), while the latter is frequently cited in influential steel-sector research (Leeson et al. 2017) and various CCS learning rate reviews (e.g., Bui et al., 2018; Thomassen et al., 2020; Malhotra and Schmidt, 2020).

Based on the included estimates, a mean CCS learning rate of 7% was used as the primary input for our models. To capture uncertainty, we applied a range of ±2 standard deviations around this mean (i.e., 7% ± 6%) to test the sensitivity of our main findings. The results demonstrate that this level of uncertainty has a limited impact on the projected costs of 20 low-carbon technologies, as well as on the optimal decarbonization pathways, emission reduction potential, and abatement costs. This highlights the robustness of our projections and supports the reliability of our core conclusions (see Notes S7–S8 for details).

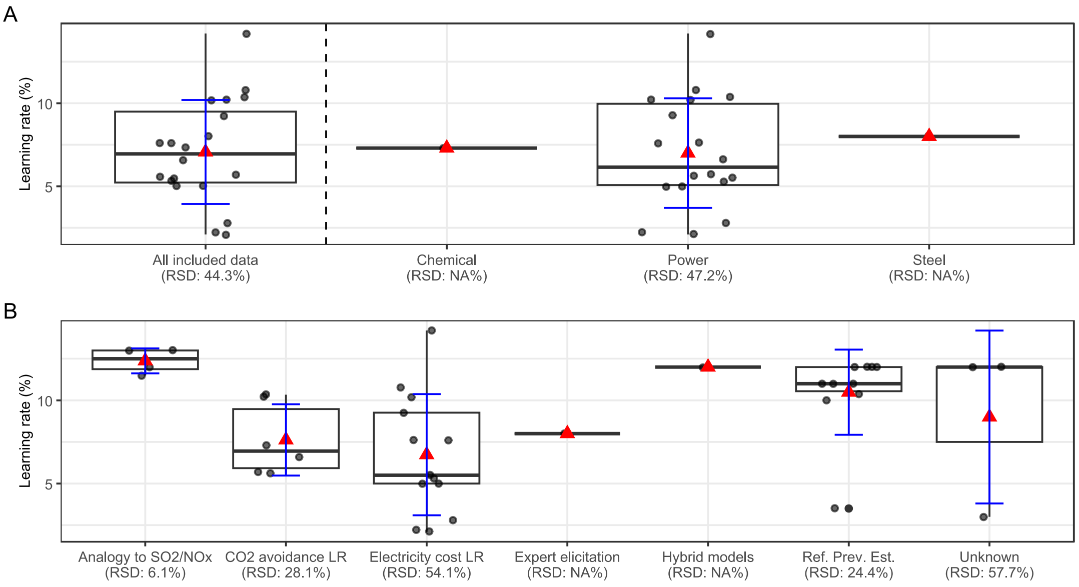

Fig. S35 Boxplot and summary statistics of literature-derived CCS learning rates. A) Columns show 20 learning rate estimates extracted from the 9 included studies, grouped by application sector (i.e., chemical, power, steel). B) Columns show 38 estimates from the 27 key studies listed in Table S13, grouped by methodological approach and cost metric: learning rates for CO_2 avoidance and electricity costs (both from included component-based studies), expert-elicited estimates (from included expert elicitation studies), and estimates from excluded studies based on analogies to SO_2/NO_x capture, hybrid learning-curve/optimization models,

references to previous CCS or energy technologies (Ref. Prev. Est.), or unspecified sources and methods. Grey points indicate standardized cost values, red triangles denote means, and blue bars show standard deviations (SD) to represent data dispersion. The boxplot displays the first quartile (Q1), median (Q2), and third quartile (Q3). Black whiskers extend to 1.5 times the interquartile range (IQR) above and below the box, with data points beyond this range considered outliers. Relative standard deviations (RSD) are also presented for each group to facilitate comparisons of data dispersion across groups with very different means.

Selected references of SI Part B (All references are detailed in the PRISMA Literature Dataset, provided as supplementary data. Please refer to the Data Availability section in the main text for more information.)

1 Garcia, M. & Berghout, N. Toward a common method of cost-review for carbon capture technologies in the industrial sector: cement and iron and steel plants. *International Journal of Greenhouse Gas Control* **87**, 142-158, doi:10.1016/j.ijggc.2019.05.005 (2019).

2 IEAGHG. Cost of CO_2 capture in the industrial sector: cement and iron and steel industries. (IEA Greenhouse Gas R&D Programme, 2018).

3 Thomassen, G., Van Passel, S. & Dewulf, J. A review on learning effects in prospective technology assessment. *Renew Sust Energ Rev* **130**, doi:10.1016/j.rser.2020.109937 (2020).

4 Rubin, E. S., Mantripragada, H., Marks, A., Versteeg, P. & Kitchin, J. The outlook for improved carbon capture technology. *Progress in Energy and Combustion Science* **38**, 630-671, doi:10.1016/j.pecs.2012.03.003 (2012).

5 Malhotra, A. & Schmidt, T. S. Accelerating Low-Carbon Innovation. *Joule* **4**, 2259-2267, doi:10.1016/j.joule.2020.09.004 (2020).

6 Sievert, K., Schmidt, T. S. & Steffen, B. Considering technology characteristics to project future costs of direct air capture. *Joule* **8**, doi:10.1016/j.joule.2024.02.005 (2024).

7 Bui, M. *et al.* Carbon capture and storage (CCS): the way forward. *Energy & Environmental Science* **11**, 1062-1176, doi:10.1039/c7ee02342a (2018).

8 Riahi, K., Rubin, E. S. & Schrattenholzer, L. Prospects for carbon capture and sequestration technologies assuming their technological learning. *Energy* **29**, 1309-1318, doi:10.1016/j.energy.2004.03.089 (2004).

9 Riahi, K., Rubin, E. S., Taylor, M. R., Schrattenholzer, L. & Hounshell, D. Technological learning for carbon capture and sequestration technologies. *Energy Economics* **26**, 539-564, doi:10.1016/j.eneco.2004.04.024 (2004).

10 Rubin, E. S., Taylor, M. R., Yeh, S. & Hounshell, D. A. Learning curves for environmental technology and their importance for climate policy analysis. *Energy* **29**, 1551-1559, doi:10.1016/j.energy.2004.03.092 (2004).

11 Ding, H., Zheng, H., Liang, X. & Ren, L. Getting ready for carbon capture and storage in the iron and steel sector in China: Assessing the value of capture readiness. *Journal of Cleaner Production* **244**, doi:10.1016/j.jclepro.2019.118953 (2020).

12 Rochedo, P. R. R. & Szklo, A. Designing learning curves for carbon capture based on chemical absorption according to the minimum work of separation. *Applied Energy* **108**, 383-391, doi:10.1016/j.apenergy.2013.03.007 (2013).

13 IEAGHG. Estimating the Future Trends in the Cost of CO_2 Capture Technologies. (IEA Greenhouse Gas R&D Programme, 2006).

14 Rubin, E. S., Yeh, S., Antes, M., Berkenpas, M. & Davison, J. Use of experience curves to estimate the future cost of power plants with CO2 capture. *International Journal of Greenhouse Gas Control* **1**, 188-197, doi:10.1016/S1750-5836(07)00016-3 (2007).

15 van den Broek, M., Hoefnagels, R., Rubin, E., Turkenburg, W. & Faaij, A. Effects of technological learning on future cost and performance of power plants with CO capture. *Progress in Energy and Combustion Science* **35**, 457-480, doi:10.1016/j.pecs.2009.05.002 (2009).

16 Li, S., Zhang, X., Gao, L. & Jin, H. Learning rates and future cost curves for fossil fuel energy systems with CO2 capture: Methodology and case studies. *Applied Energy* **93**, 348-356, doi:10.1016/j.apenergy.2011.12.046 (2012).

17 Kang, J. N. *et al.* The Prospects of Carbon Capture and Storage in China's Power Sector under the 2 °C Target: A Component-based Learning Curve Approach. *International Journal of Greenhouse Gas Control* **101**, doi:10.1016/j.ijggc.2020.103149 (2020).

18 Zhou, L., Duan, M. S., Yu, Y. D. & Zhang, X. L. Learning rates and cost reduction potential of indirect coal-to-liquid technology coupled with CO capture. *Energy* **165**, 21-32, doi:10.1016/j.energy.2018.09.150 (2018).

19 Leeson, D., Mac Dowell, N., Shah, N., Petit, C. & Fennell, P. S. A Techno-economic analysis and systematic review of carbon capture and storage (CCS) applied to the iron and steel, cement, oil refining and pulp and paper industries, as well as other high purity sources. *International Journal of Greenhouse Gas Control* **61**, 71-84, doi:10.1016/j.ijggc.2017.03.020 (2017).

20 Lee, H., Lee, J. & Koo, Y. Economic impacts of carbon capture and storage on the steel industry–A hybrid energy system model incorporating technological change. *Applied Energy* **317**, doi:10.1016/j.apenergy.2022.119208 (2022).

21 Moglianesi, A., Keppo, I., Lerede, D. & Savoldi, L. Role of technology learning in the decarbonization of the iron and steel sector: An energy system approach using a global-scale optimization model. *ENERGY* **274**, doi:10.1016/j.energy.2023.127339 (2023).

22 NETL. Technology Learning Curve (FOAK to NOAK), Quality Guidelines for Energy Systems Studies. (National Energy Technology Laboratory 2013).

23 IEA. CCUS in clean energy transitions. (International Energy Agency, 2020).

24 GCCSI. Policy Priorities to Incentivise Large Scale Deployment of CCS. (Global CCS Institute, 2019).

Part C - Systematic review of initial hydrogen cost and learning rate

Note S10. PRISMA of initial hydrogen cost and learning rate.

10.1 Initial cost of hydrogen production.

10.1.1 Background and motivation.

Hydrogen is a key solution for decarbonizing the iron and steel sector. However, the production of hydrogen remains immature, leading to high costs and significant uncertainties. Over the past two decades, numerous studies have projected hydrogen cost reductions, but reviews by Frieden et al. (2024) and Miyagawa et al. (2022) reveal substantial variability in these forecasts due to differences in methodologies, assumptions, and perspectives, which limits their direct application[1,2]. To address this, we independently projected future hydrogen costs through a learning curve model and prioritized systematic reviews of key inputs such as initial costs and learning rates instead of relying on existing cost forecasts. This approach provides a solid basis for modeling the cost trajectory of hydrogen-based steelmaking technologies and their role in achieving low-carbon steel production.

10.1.2 Research question and objectives.

This section aims to determine the initial levelized cost of hydrogen through a systematic review. Among the various hydrogen production technologies, our focus is on hydrogen produced via water electrolysis powered by renewable energy, while excluding grey hydrogen based on fossil fuels and hydrogen derived from biomass. Niche producing processes, like photoelectrochemical or photocatalytic water splitting, are also excluded. Additionally, we concentrate on hydrogen production rather than its applications in other sectors such as chemical processing, transportation, energy storage, or fuel use, in order to minimize the influence of additional technical assumptions and external factors on reported cost estimates.

10.1.3 Search strategy.

A comprehensive literature search was conducted using the Web of Science and Scopus databases due to their extensive coverage of high-quality, peer-reviewed journals. The search strategies outlined in Table S14 were applied to article titles, abstracts, and keywords. We also manually screened the reference lists of included studies to ensure no significant articles were missed. Additionally, grey literature from major industry and climate organizations, such as the International Energy Agency (IEA), International Renewable Energy Agency (IRENA), and Hydrogen Council, was manually included to complement academic literature. To qualify for inclusion, grey literature reports needed to satisfy two criteria: they must have been cited in academic studies and contain the required quantitative data.

Table S14 Search strategy in the Web of Science and Scopus databases.

Objectives	Keywords	Field of research	Period
Initial levelized cost of hydrogen	(hydrogen OR H2) AND (“cost reduction*” OR “future cost*” OR “cost projection” OR “cost forecast”)	Title, Abstract, Keywords	Start date-2024
Learning rate of hydrogen cost	(hydrogen OR H2) AND ("learning curve*" OR "experience curve*" OR "techn* learning" OR "cost reduction*" OR "future cost*" OR "learning rate*" OR "experience rate*")	Title, Abstract, Keywords	Start date-2024

10.1.4 Literature screening, inclusion and exclusion criteria.

Literature identification. As shown in Fig. S36, a total of 6065 records were initially retrieved from the Web of Science and Scopus databases using the search strategy detailed in Table S14. Following the removal of 691 records due to duplication, or categorization as patents, news or editorials, 5374 articles, conference papers, review articles, and book chapters remained for further screening. Additionally, 155 studies from previous systematic and quantitative reviews on hydrogen production costs were incorporated as supplementary sources after duplicates were removed.

Title and Abstract Screening. The remaining 5374 records underwent independent screening by two researchers based on the inclusion and exclusion criteria detailed in Table S15. This process excluded 5183 studies unrelated to hydrogen production or its cost assessment, categorized as follows:

a) 2834 studies unrelated to hydrogen production processes, including:

- 2453 studies from unrelated fields (e.g., mathematics, physics, agronomy, medicine).
- 381 studies mentioning hydrogen in their titles or abstracts but primarily focusing on unrelated technologies, such as carbon capture and storage (CCS), direct air capture (DAC), or catalysts.

b) 1623 studies addressing hydrogen applications or integration in other sectors. These studies explored the use of hydrogen as:

- Feedstock for ammonia or methanol production.
- Fuel cells in transportation (e.g., vehicles, aviation).
- An energy storage medium in renewable energy systems.

While some of these studies discussed hydrogen costs, they were excluded because integrating hydrogen with other sectors introduced technical assumptions and design complexities beyond the scope of production cost assessment.

c) 389 studies focusing on other aspects of hydrogen systems:

These studies investigated hydrogen storage, transportation, pipelines, leakage, supply chains, and international trade. They were excluded as they did not directly address hydrogen production.

d) 337 studies focused on technical advancements in hydrogen production, such as electrolyzer development or renewable energy system optimization. While some performed techno-economic assessments, they did not focus on common hydrogen production technologies. These also included studies on policies supporting hydrogen development or public awareness impacts.

Full-text Screening. After abstract screening, 186 out of 191 retained articles were assessed for full-text eligibility, with 5 studies excluded due to the unavailability of its full text. To identify levelized cost estimates for green hydrogen, 95 studies were excluded for the following reasons:

e) 81 studies reviewed or cited previous research on hydrogen costs without presenting original cost estimates.

f) 14 studies exclusively provided cost estimates for grey or blue hydrogen or alternative production methods, such as photochemical or bio-hydrogen production, instead of green hydrogen via water electrolysis.

As a result, 91 articles from the original 6065 records were included. In addition, 5 organizational reports from reputable sources (e.g., IEA, IRENA, PwC, and Hydrogen Council) and 15 academic studies were manually identified through citation searching. In addition to our own search, 50 non-duplicate studies on green hydrogen identified from previous reviews were included, resulting in a total of 161 studies for data extraction.

All screened references are detailed in the PRISMA Literature Dataset, available as supplementary material (see Data Availability section in the main text).

Table S15 Eligibility criteria for full-text assessment.

Inclusion Criteria	Exclusion Criteria
- Articles, conference papers, reviews, thesis, or book chapter	- Other document types (e.g., patents, news articles)
- Related to hydrogen-related processes	- Unrelated fields or technologies
- Hydrogen as a primary subject	- Use of hydrogen as a medium (e.g., ammonia production, energy storage)
- Addresses hydrogen production costs	- Limited to hydrogen transportation, storage, supply chain, leakage, or trade
- Provides original cost estimates	- Lacks original cost estimates
- Covers water electrolysis with renewable energy as a production method	- Focuses on alternative production methods (e.g., photochemical, bio-hydrogen)

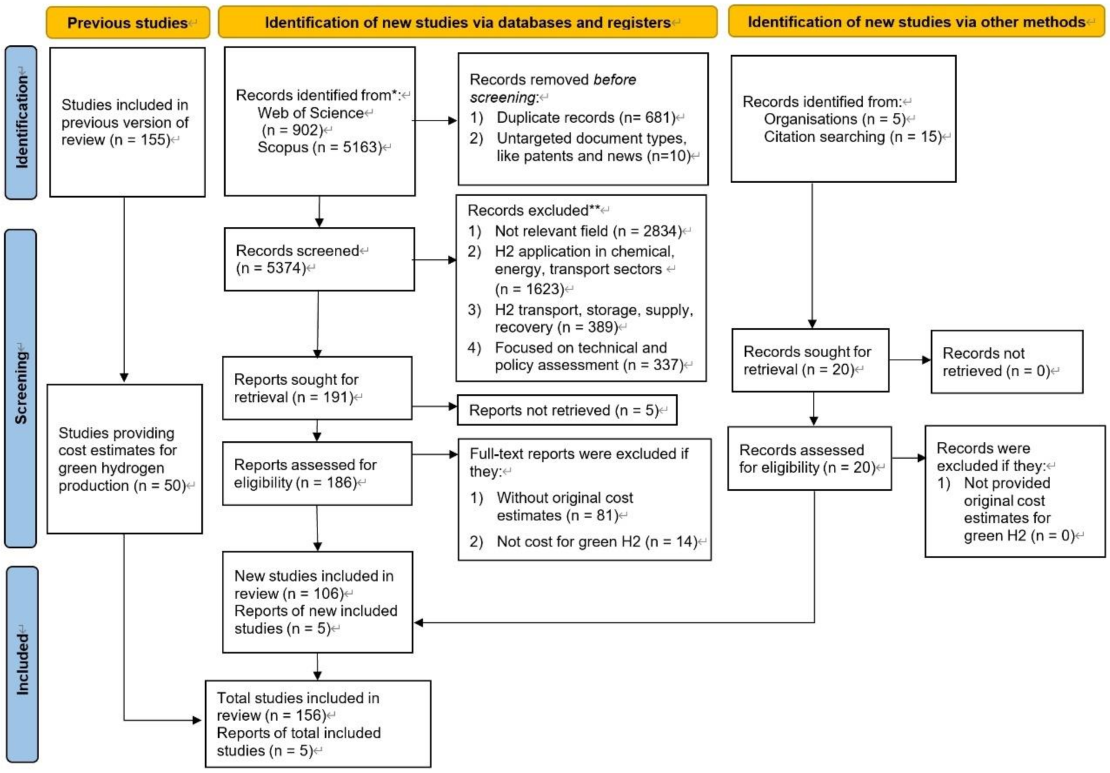

Fig. S36 Literature search procedures. PRISMA flow diagram illustrating the procedures (i.e., identification, screening, eligibility and inclusion) for obtaining literature values for the initial levelized cost of hydrogen production via water electrolysis.

10.1.5 Data extraction and processing.

Literature characteristics and selection. The cost of hydrogen production is primarily determined by the cost of renewable electricity and the cost of electrolyzers. Factors affecting either of these components can significantly influence the levelized cost of hydrogen (LCOH), leading to considerable uncertainty in cost estimates. Previous reviews on hydrogen production costs have underscored the substantial variation in reported LCOH values. For example, Miyagawa et al. (2022) compiled cost estimates from articles published between 1979 and 2020, with a focus on the energy sources used. Frieden et al. (2024) reviewed academic and grey literature from 2000 to 2023, examining cost differences across electrolysis technologies and production locations. Gómez et al. (2024) summarized updated cost estimates for various countries from 2020 to 2024. To enhance the comparability of literature values, we systematically extracted key characteristics and cost estimates from studies identified through our PRISMA screening process. The extracted characteristics included authorship, publication year, year of estimation, energy source (e.g., solar, wind, or unspecified renewable electricity), electrolyzer technology (ALK, PEM, AEM, SOEC, or unspecified), cost units and currency, and geographic focus, as summarized in Table S16. This approach enables consistent comparisons and clarifies the assumptions underlying LCOH estimates.

Table S16 Key literature characteristics on the cost of green hydrogen.

Type	Category	Count
Energy source	Energy: Hybrid system	14
	Energy: Solar	51
	Energy: Wind	50
	Energy: Geothermal	1
	Energy: Hydroelectric	4
	Energy: Not specified	74
Electrolysis technology	Electrolysis: ALK	28
	Electrolysis: PEM	64
	Electrolysis: SOEC	54
	Electrolysis: Not specified	89
Geographic location	Locations: Africa	10
	Locations: Europe	61
	Locations: Middle East	12
	Locations: North America	20
	Locations: South America	10
	Locations: Asia	27
	Locations: Pacific	4
	Locations: Not specified	23

Note: Each type includes a total of 161 studies, comprising 106 academic articles systematically retrieved and screened from the Web of Science and Scopus, 5 reports identified through citation searching, and 50 non-duplicated studies from previous reviews conducted by other researchers.

Data extraction and summary statistics. We extracted 371 estimates of the levelized cost of hydrogen (LCOH) via renewable electrolysis from 161 included studies. Costs reported in various units, such as \$/MWh, \$/kWh, \$/GJ, and \$/kmol, were converted to \$/kg H_2 using the conversion rates provided by Miyagawa et al. (2022). To facilitate cross-regional and temporal comparisons, we standardized the cost values—originally reported in different currencies and years—into 2021 USD using historical exchange rates and the Chemical Engineering Plant Cost Index (CEPCI), following the methodology of Leeson et al. (2017).

As shown in Fig. S37, LCOH values exhibit a gradual downward trend over time, ranging from a peak of \$50/kg H_2 in 2007 to a low of \$1.3/kg H_2 in 2020, reflecting both technological progress and variations in regional and techno-economic assumptions. Descriptive statistics on all extracted estimates show a mean cost of \$7.0/kg H_2, accompanied by a high standard deviation (SD) of 4.8 and a relative standard deviation (RSD) of 69% (Fig. S38A). We then conducted subgroup analyses on estimation year, geographic region, energy source, and electrolysis technology. Driven largely by declining renewable-electricity costs, post-2020 estimates are generally lower and more tightly clustered than earlier values. Regionally, mean LCOH spans \$3.80/kg H_2 in South America to \$7.10/kg H_2 in Europe, with intra-regional RSDs of 30–76% (Fig. S38B). Cost differences across electrolysis technologies and energy sources within each region remain minor (Fig. S38C-D), likely because methodological inconsistencies and varied assumptions in the literature obscure true performance

distinctions.

Data selection and uncertainty. This systematic review aims to identify region-specific hydrogen production cost estimates as inputs for our learning-curve model. Rather than averaging disparate studies which risks conflating fundamentally different methodologies and obscuring true regional differences, we prioritized multi-region estimates to ensure consistent, comparable assumptions across all regions.

Among all included studies, fifteen provided cost estimates for two to six countries each, while only four covered more than ten countries. PwC (2021) stands out by providing initial LCOH estimates for 20 countries worldwide, spanning all ten regions used in our modeling, and aligning closely with the ranges reported in the other multi-region studies. In contrast, the remaining three studies lack global regional coverage: Janssen et al. (2022) focused on 30 European countries; Gado et al. (2021) evaluated 10 Middle Eastern countries; and Kigle et al. (2024) produced high-resolution estimates for 94 countries (256 locations), but detailed location-level data remain publicly unavailable. Given its broad geographic coverage and methodological consistency, we adopted PwC's region-specific LCOH estimates as the baseline inputs for our learning-curve model, which underpin results in the main text.

To capture uncertainty, we defined bounds at the mean ± 2 SD of all post-2020 estimates, covering roughly 95 % of data regardless of distribution shape. Because the lower bound (mean – 2 SD) fell below zero, we substituted the minimum literature value and retained mean + 2 SD as the upper bound, yielding a range of \$1.32–14.30/kg H_2. Sensitivity analyses over this interval show that such variation in initial hydrogen costs has only a modest effect on projected low-carbon technology costs, optimal decarbonization pathways, emission-reduction potential, and abatement costs, underscoring the robustness of our model projections (see details in Note S7-S8 in SI Part A).

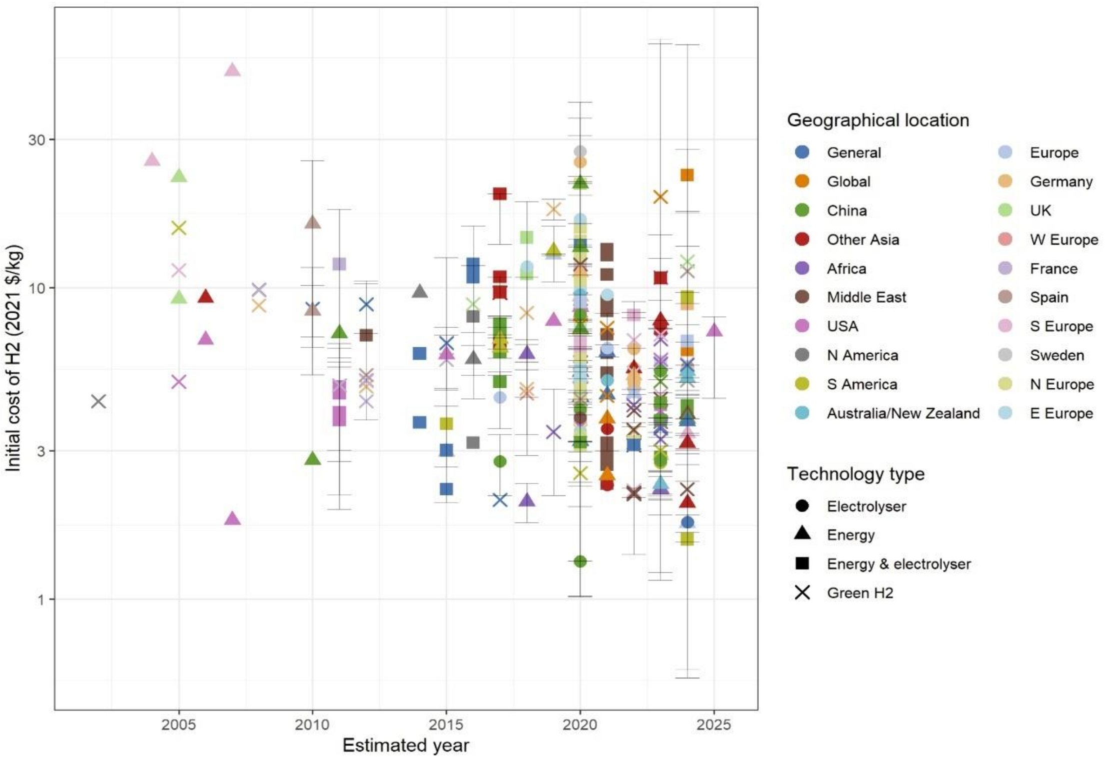

Fig. S37 Literature values on initial cost of hydrogen production via renewable electrolysis. Cost estimates were extracted from 161 key studies included after full-text eligibility assessment using the PRISMA framework. The year shown reflects the target year of the estimate, or the publication year when the former was not reported. The original values, reported in various currencies and years, were standardized to 2021 USD using historical exchange rates and the Chemical Engineering Plant Cost Index (CEPCI), following the methodology of Leeson et al. (2017). The points represent the average values, while the bars depict the range between the minimum and maximum values reported in the same literature. Colors denote geographical regions. The shapes of the points indicate whether the studies emphasize the energy type, the electrolysis technology, both, or simply refer to "green hydrogen" without detailing technical assumptions. More reference information is provided in the PRISMA Literature Dataset (see Data Availability section in the main text).

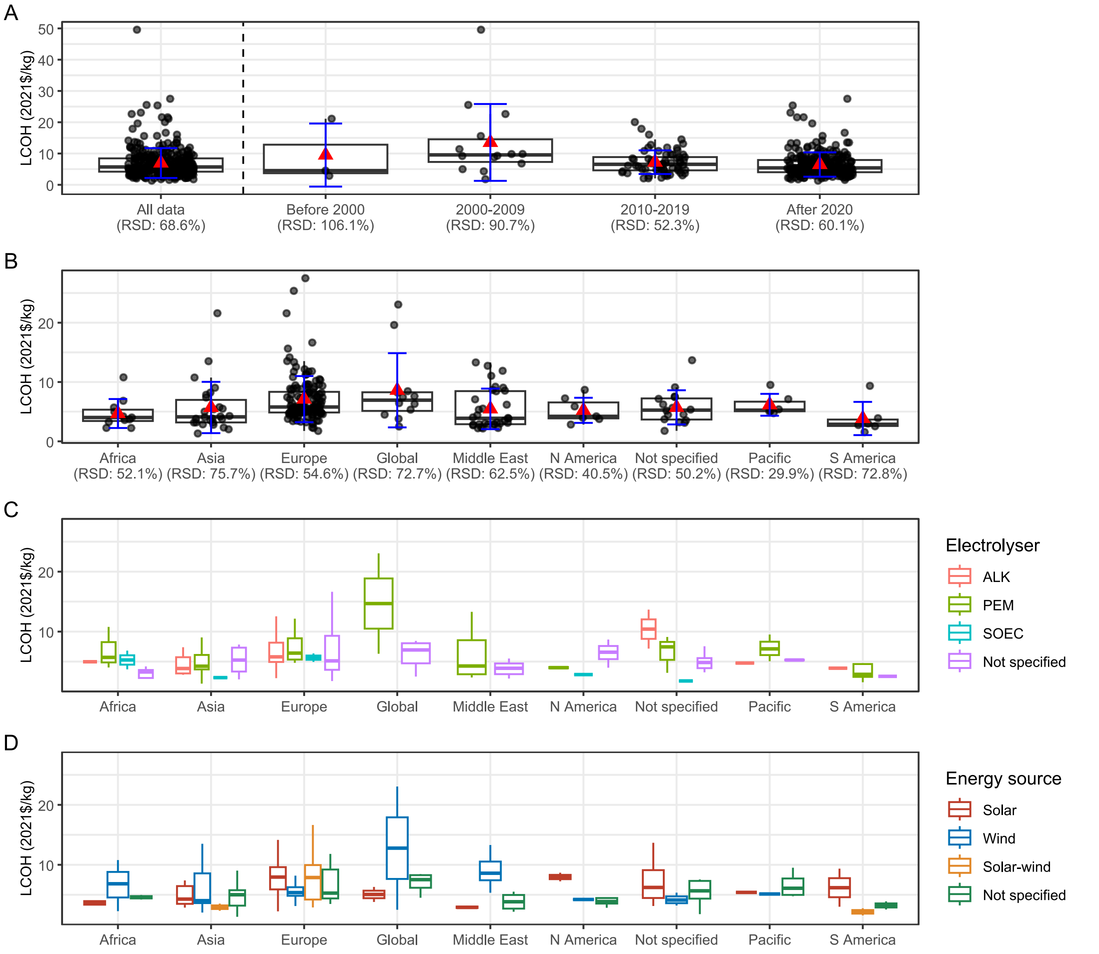

Fig. S38 Boxplot and summary statistics of literature values for initial levelized cost of hydrogen production Each panel presents literature data grouped by specific characteristics: A) from left to right, columns display all data from the included studies, organized by the period of cost estimation; B) columns show region-specific cost estimated after 2020, including Africa, Asia, the Middle East, Pacific, Europe, South and North America, multi-regional data (labeled as Global), and non-region-specific data; C) and D) further disaggregate the region-specific data from panel B by electrolyser type and energy source, respectively. Grey points represent standardized cost values, red triangles indicate means, and blue bars depict standard deviations. The boxplot shows the first quartile, median, and third quartile, with black whiskers extending to 1.5 times the interquartile range (IQR) above and below the median. Relative standard deviations are provided to compare data dispersion across groups with differing means.

10.2 Learning rate of hydrogen.

10.2.1 Background and motivation.

Several studies have forecasted green hydrogen production costs using methods such as bottom-up techno-economic assessments, learning curves, and expert elicitation[1]. However, these forecasts exhibit significant variability, and reports of learning rates specific to hydrogen production are scarce. Due to the limited empirical data available for green hydrogen, most studies rely on learning rates for renewable energy and electrolysis to estimate the levelized cost of green hydrogen, rather than deriving hydrogen-specific learning rates. Therefore, to project hydrogen-based technology costs using a component-based learning curve, it is crucial to identify appropriate learning rates for either green hydrogen or electrolysis to incorporate into our model.

10.2.2 Research question and objectives.

This systematic review aims to identify learning rates for green hydrogen production, where available, and for renewable electrolysis, to be used in a component-based learning curve model. While the primary focus is on green hydrogen production, we extended the scope to include learning rates for electrolysis technologies commonly applied in hydrogen cost projections due to limited empirical data of green hydrogen.

10.2.3 Search strategies.

A comprehensive literature search on the learning rate of green hydrogen was conducted using the Web of Science and Scopus databases, employing the search strategies outlined in Table S14. The search covered titles, abstracts, and keywords, encompassing studies published from the earliest available year to 2024. To ensure comprehensive coverage, we manually screened the reference lists of identified articles. Additionally, grey literature from relevant organizations, such as the International Energy Agency (IEA), the International Renewable Energy Agency (IRENA), and the Hydrogen Council, was included to complement academic studies, provided these sources were cited in scholarly publications.

10.2.4 Literature screening, inclusion and exclusion criteria.

Literature identification. As shown in Fig. S39, an initial total of 3025 records were retrieved from the Web of Science and Scopus databases. Based on the inclusion and exclusion criteria outlined in Table S17, 725 records were removed prior to screening for the following reasons:

- Duplicates (n = 703), and
- Untargeted document types, such as patents, news articles, and editorial materials (n = 22).

Title and Abstract Screening. The remaining 2300 records were independently screened by two researchers based on their titles and abstracts. This process resulted in

the exclusion of 2196 records unrelated to hydrogen production, including:

- 842 records focused on unrelated research areas (e.g., mathematics, physics, chemistry, agriculture, medicine, biology, materials science, education, and sociology),
- 1262 records focused on hydrogen applications in other sectors (n = 1174) or hydrogen transportation, storage, supply, trade, or recovery rather than hydrogen production (n = 88), and
- 92 records focused on technological innovation, static costs, or policy assessment rather than learning effects.

Full-text Screening. During this phase, additional exclusion criteria were applied to concentrate on studies providing learning rate values. This led to the exclusion of 95 records for the following reasons:

- Lacked learning rate values (n = 59),
- Provided learning rates for unrelated energy technologies, grey or blue hydrogen instead of electrolysis or green hydrogen (n = 8),
- Lacked a clear source of learning rate estimates (n = 2), and
- Cited learning rates from previous studies rather than reporting original estimates (n = 26)

As a result, only 9 academic articles were included. Additionally, 4 reports from authoritative organizations, such as the International Energy Agency (IEA), the International Renewable Energy Agency (IRENA), and the Hydrogen Council, were included. In total, 13 studies comprising academic articles and organizational reports were selected for further review and data extraction.

All screened references are detailed in the PRISMA Literature Dataset, available as supplementary material (see Data Availability section in the main text).

Table S17 Eligibility Criteria for Full-Text Assessment.

**Inclusion Criteria**	**Exclusion Criteria**
Articles, conference papers, reviews, thesis, or book chapter	Other document types (e.g., patents, news, editorials)
Related to hydrogen-related processes	Unrelated fields or technologies
Hydrogen as a primary subject	Studies where hydrogen is used as a medium (e.g., ammonia production, energy storage)
Focused on hydrogen production	Limited to hydrogen transportation, storage, supply chain, leakage, or trade
Provision of learning rates	Lack of learning rate values or unclear sources
Learning rates specific to green hydrogen or electrolysis	Learning rates provided only for other technologies
Provision of original estimates	Cited learning rates from previous studies

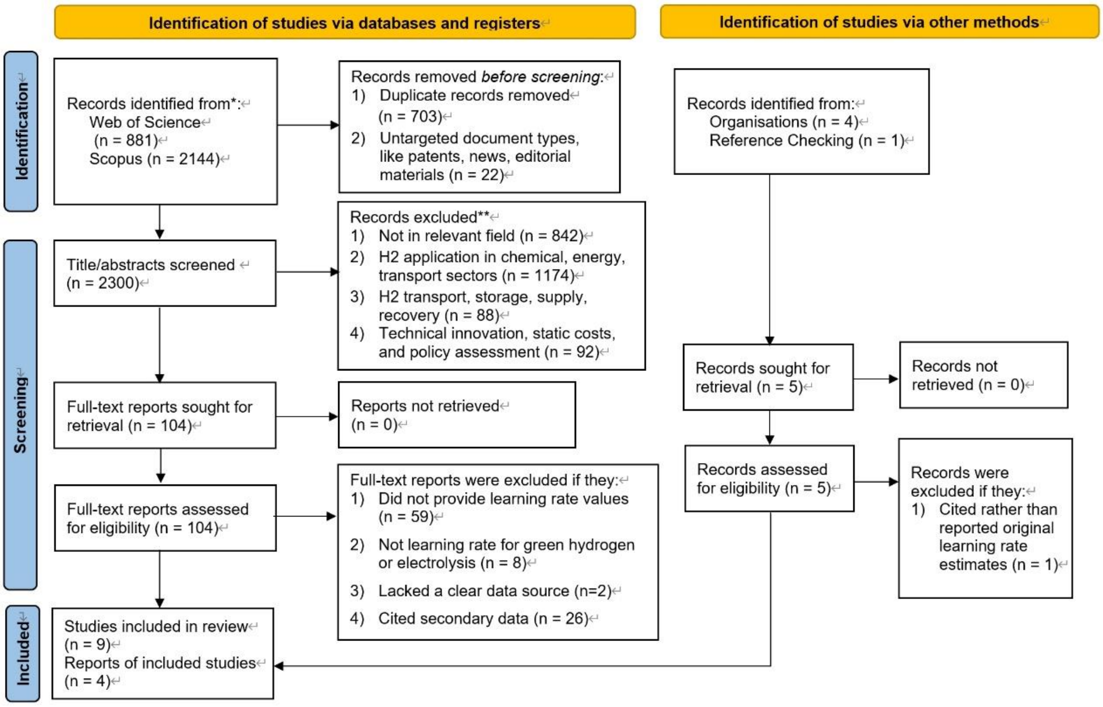

Fig. S39 Literature search procedures. PRISMA flow diagram illustrating the procedures (i.e., identification, screening, eligibility and inclusion) for obtaining literature values for the learning rate of green hydrogen or electrolyser cost.

10.2.5 Data extraction and processing.

Literature characteristics. Table S18 summarizes the characteristics of the 13 included studies that provide original learning rate estimates. Key factors potentially influencing the estimates were extracted, including the estimation methodology, electrolyser type, associated renewable energy source, publication year, and year of estimation, where applicable. For comparison, the table also presents 28 representative but excluded studies, which did not provide new estimates but instead cited previous studies. To better understand citation patterns, we reviewed the references of these 28 studies and confirmed that all cited estimates originated from the 13 included studies. This reference tracing offers insight into which sources are most frequently cited in the current literature, reflecting the foundational role of certain key studies in this topic.

Among the included studies, Revinova et al. (2023) was the only one to report a learning rate for green hydrogen production using a component-based learning curve. They projected future hydrogen costs by applying learning rates for two types of electrolysers and two renewable energy sources and back-calculated the learning rate by fitting predicted costs to projected installed capacities. However, their results rely on multiple technical assumptions, introducing considerable uncertainty. Therefore, we did not directly adopt their reported learning rate of hydrogen in our model.

Other included studies primarily focused on calculating learning rates for electrolysis technologies. Böhm et al. (2019) estimated the learning rates for three types of electrolysers (i.e., ALK, PEM, and SOEC) by aggregating component-level learning rates within electrolysis systems. Schoots et al. (2008) and Krishnan et al. (2020) derived learning rates for ALK and PEM electrolysers based on historical empirical data spanning 1940–2016, while Reksten et al. (2022) and Glenk et al. (2023) fitted learning curves using estimated and projected values from the literature for 2000–2030. Schmidt et al. (2017) employed expert elicitation, collecting insights from ten academic and industry experts to determine the learning rates. IEA (2021) and IRENA (2020) synthesized learning rates from existing literature for their model scenarios. Lerede et al. (2024) provided learning rate input parameters for the endogenous TEMOA-Europe model. Zun et al. (2023) combined learning curves with bottom-up techno-economic cost calculations to estimate learning rates.

In terms of citation patterns, Schmidt et al. (2017) was the most frequently cited source, followed by IRENA (2020), Böhm et al. (2019), Glenk et al. (2023), and Schoots et al. (2008). This suggests a growing consensus on data sources, with a few influential studies shaping much of the subsequent literature and potentially guiding prevailing assumptions about learning rates.

Table S18 Key literature characteristics on learning rates of green hydrogen and electrolysis.

No.	Author	Type	Technology	Method
1	Schoots, et al. (2008)	Single-electrolyser	ALK	Empirical learning curve (1940-2007)
2	Schmidt, et al. (2017)	Multi-electrolyser	ALK	Expert elicitation
			PEM	
			SOEC	
3	Böhm, et al. (2019)	Multi-electrolyser	ALK	Component-based learning curve
			PEM	
			SOEC	
4	Hydrogen Council (2020)	Multi-electrolyser	ALK	Analogous to fuel cell
			PEM	
5	Krishnan, et al. (2020)	Single-electrolyser	ALK	Empirical learning curve (1956-2016)
6	Reksten, et al. (2022)	Multi-electrolyser	ALK	Learning curve from literature data (2000-2030)
			PEM	
7	Glenk et al. (2023)	Multi-electrolyser	ALK	Learning curve from literature data (2000-2020)
			PEM	
			SOEC	
8	Revinova, et al. (2023)	Multi-electrolyser	ALK	Empirical learning curve
			PEM	
			Green H2	Component-based learning curve
		Energy	Solar	Empirical learning curve
		Energy	Wind	
9	Zun, et al. (2023)	Multi-electrolyser	ALK	Learning curve and bottom-up calculation
			PEM	
10	Lerede, et al. (2024)	Multi-electrolyser	ALK	Endogenous model
		Multi-electrolyser	PEM	
		Multi-electrolyser	SOEC	
11	Bloomberg NEF (2020)	Not-specified	Electrolyser	Literature review
12	IRENA (2020)	Not-specified	Electrolyser	Literature review
		Energy	Solar	Literature review
13	IEA (2021)	Not-specified	Electrolyser	Literature review
14	Ouassou, et al. (2021)	Multi-electrolyser	ALK, PEM,SOEC	Böhm, et al. (2019); Schoots, et al. (2008)
15	Moglianesi, et al. (2023)	Not-specified	Electrolyser	Böhm, et al. (2019)
16	Ordonez, et al. (2023)	Not-specified	Electrolyser	
17	Roeder, et al. (2024)	Multi-electrolyser	ALK, PEM,SOEC	
18	Rezaei, et al. (2024)	Single-electrolyser	PEM	Glenk et al. (2023)
19	Bühler, et al. (2025)	Multi-electrolyser	ALK, PEM	
20	Koj, et al. (2025)	Single-electrolyser	PEM	
21	Xu, et al. (2023)	Multi-electrolyser	ALK,PEM	Hydrogen Council (2020)
22	Gandhi, et al. (2022)	Not-specified	Electrolyser	IRENA (2020)
23	Loisel, et al. (2022)	Single-electrolyser	PEM	
24	Huang, et al. (2023)	Multi-electrolyser	ALK,PEM	
25	Wang, et al. (2023)	Not-specified	Electrolyser	
26	Zhen, et al. (2023)	Multi-electrolyser	ALK,PEM	
27	Liu, et al. (2024)	Multi-electrolyser	ALK	
28	Man, et al. (2024)	Multi-electrolyser	ALK,PEM	
29	Krishnan, et al. (2023)	Not-specified	Electrolyser	Krishnan, et al. (2020)
30	Rezaei, et al. (2022)	Not-specified	Electrolyser	Rezaei, et al. (2022)
31	Liu, et al. (2024)	Multi-electrolyser	PEM	Schmidt, et al. (2017)
32	Syauqi, et al. (2024)	Not-specified	Electrolyser	
33	Cao, et al. (2024)	Not-specified	Electrolyser	
34	Lee, et al. (2019)	Single-electrolyser	ALK	
35	Lane, et al. (2021)	Single-electrolyser	PEM	
36	Ouassou, et al. (2021)	Multi-electrolyser	ALK, PEM,SOEC	
37	Lee, et al. (2023)	Single-electrolyser	ALK	
38	Zeyen, et al. (2023)	Single-electrolyser	ALK	
39	Cattry, et al. (2024)	Not-specified	Electrolyser	
40	Nicodemus, et al. (2018)	Not-specified	Electrolyser	Schoots, et al. (2008)
41	Detz, et al. (2018)	Multi-electrolyser	ALK, PEM,SOEC	

Note: a) The 41 studies comprise 13 that provided original learning rate estimates (colored in green) and 28 that cited previous studies without offering new estimates (no color). b) The "Technology" column specifies the focus of the learning rate, such as varied electrolyzers, different types of renewable electricity, or green hydrogen production. For electrolyzers, the

technologies considered include alkaline (ALK), proton exchange membrane (PEM), solid oxide electrolyser cell (SOEC), and unspecified types. For renewable energy, solar and wind are the most commonly studied sources. "Green H2" represents the learning rate for the levelized cost of green hydrogen production. c) The "Method" column describes the calculation approach for original learning rate estimates or references cited for reported values. "Literature Review" indicates that the study synthesized insights from multiple references rather than relying on a single source. d) More reference information is provided in the PRISMA Literature Dataset (see Data Availability section in the main text).

Data extraction and summary statistics. Fig. S40 presents 26 original estimates for electrolysis learning rate from 13 key studies, along with corresponding estimation methods, technical assumptions, and years of estimation. A notable increase in the number of reported learning rates has occurred since 2020. Among the studies, Reksten et al. (2022) reported the highest learning rate estimate derived from fitted literature data, while Schmidt et al. (2017) exhibited the widest uncertainty range based on expert elicitation. To ensure the consistency and comparability of the extracted data, we applied descriptive statistical measures such as mean, median, quartiles, standard deviation (SD), and relative standard deviation (RSD) to characterize trends and variability (as shown in Fig. S41).

The mean learning rate across all studies was 18%, with a standard deviation (SD) of 6% and a relative standard deviation (RSD) of 35%. Learning rates by different electrolyser types were 16% for alkaline (ALK), 18% for proton exchange membrane (PEM), and 23% for solid oxide electrolysers (SOEC). In terms of estimation methods, studies based on analogies reported a mean learning rate of 11%, while estimates from expert elicitation averaged 21%. Studies employing learning curve models produced estimates ranging from 9% to 21%, reflecting differences in the types of input data used, such as empirical observations, component-level data, engineering calculations, or model-based parameters. Literature reviews, primarily from IEA and IRENA, reported learning rate estimates without specifying the electrolyser type, with an average of 17%.

Data selection and uncertainty. Following a systematic review and rigorous screening, we adopted the overall mean of all extracted estimates (18 %) as the baseline input for our learning-curve model. This value aligns closely with rates reported in several widely cited studies, such as Böhm et al. (2019), Schmidt et al. (2017), Schoots et al. (2008), and IRENA (2020).

To account for uncertainty, we adopted a range based on the mean ± 2 standard deviations (SD) of all original learning rate estimates (i.e., 18% ± 12%) for sensitivity analyses. The results show that even a ±2 SD variation in the electrolyser learning rate has a relatively limited effect on future costs of 20 low-carbon technologies, optimal decarbonization pathways for global steel plants, emission reduction potential, and abatement costs. These findings highlight the robustness of our projections and conclusions (see Notes S7–S8 in the SI Part A for details).

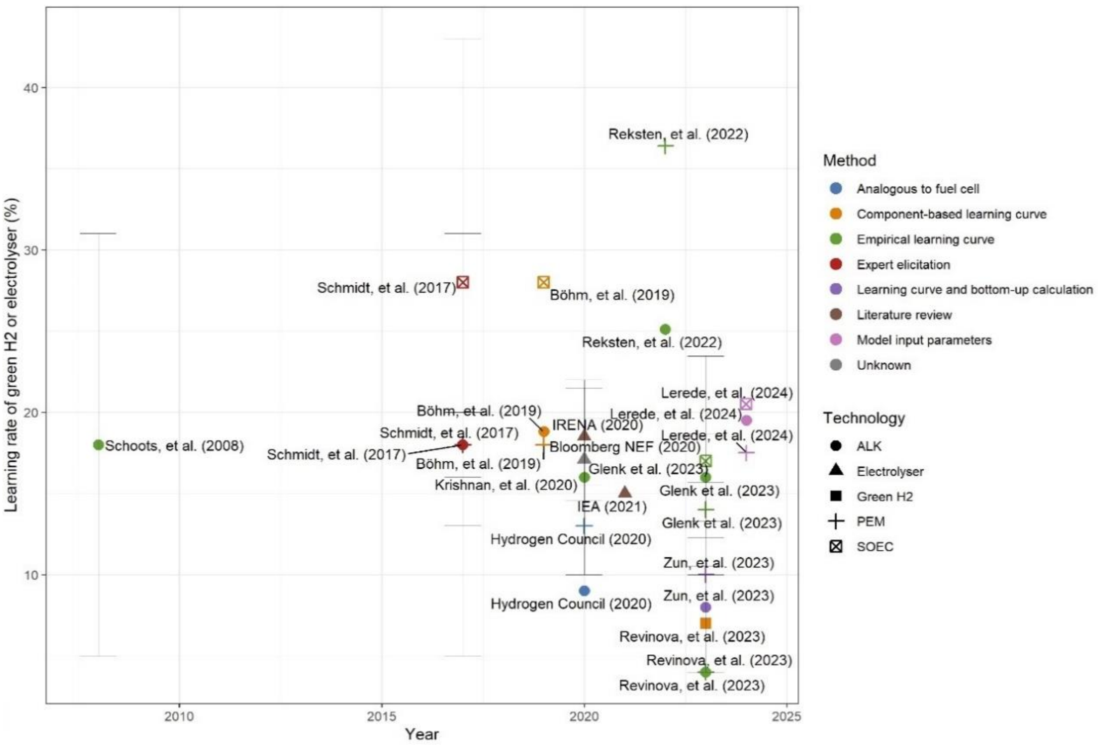

Fig. S40 Literature values for learning rates of green hydrogen costs and electrolyser costs. Learning rate estimates were extracted from 13 key studies providing original estimates, identified through a systematic review conducted using the PRISMA framework. The points represent the primary learning rate estimates reported in each study, while the bars illustrate the minimum and maximum values provided in the corresponding literature. Colors indicate the estimation method, and point shapes denote the electrolysis technology or green hydrogen production pathway to which the learning rates apply. More reference information is provided in the PRISMA Literature Dataset (see Data Availability section in the main text).

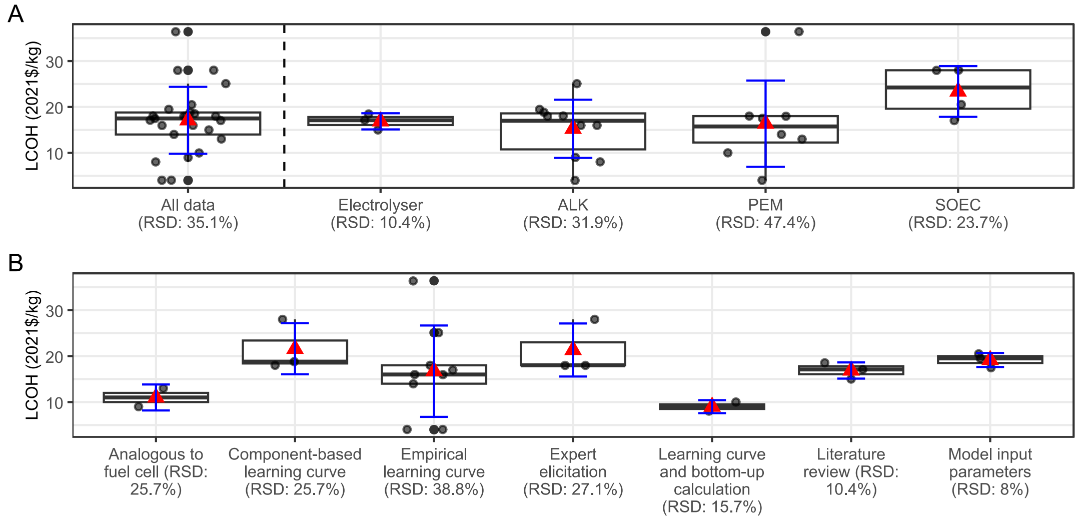

Fig. S41 Boxplot and summary statistics of literature-reported electrolysis learning rates. Each column represents literature data grouped by specific characteristics: A) from left to right, columns display all data from the 13 key studies, followed by data categorized by electrolysis

technologies, including alkaline (ALK), proton exchange membrane (PEM), solid oxide electrolyser cell (SOEC), and unspecified types (denoted as Electrolyser). B) Columns display data grouped by estimation method, including analogies, various learning curve models, expert elicitation, and literature reviews. Grey points indicate reported learning rates, red triangles denote means, and blue bars show standard deviations (SD) to represent data dispersion. The boxplot displays the quartiles, while black whiskers extend to 1.5 times the interquartile range (IQR) above and below the median. Relative standard deviations (RSD) are also presented for each group to facilitate comparisons of data dispersion across groups with very different means.

Selected references of SI Part C (All references are detailed in the PRISMA Literature Dataset, provided as supplementary material. Please refer to the Data Availability section in the main text for more information.)

1 Frieden, F. & Leker, J. Future costs of hydrogen: a quantitative review. *Sustainable Energy & Fuels* **8**, 1806-1822, doi:10.1039/d4se00137k (2024).

2 Miyagawa, T. & Goto, M. Hydrogen Production Cost Forecasts since the 1970s and Implications for Technological Development. *Energies* **15**, 4375, doi:10.3390/en15124375 (2022).

3 Thomassen, G., Van Passel, S. & Dewulf, J. A review on learning effects in prospective technology assessment. *Renew Sust Energ Rev* **130**, doi:ARTN 109937

10.1016/j.rser.2020.109937 (2020).

4 Rubin, E. S., Mantripragada, H., Marks, A., Versteeg, P. & Kitchin, J. The outlook for improved carbon capture technology. *Progress in Energy and Combustion Science* **38**, 630-671, doi:10.1016/j.pecs.2012.03.003 (2012).

5 Malhotra, A. & Schmidt, T. S. Accelerating Low-Carbon Innovation. *Joule* **4**, 2259-2267, doi:10.1016/j.joule.2020.09.004 (2020).

6 Sievert, K., Schmidt, T. S. & Steffen, B. Considering technology characteristics to project future costs of direct air capture. *Joule* **8**, doi:10.1016/j.joule.2024.02.005 (2024).

7 Bui, M. *et al.* Carbon capture and storage (CCS): the way forward. *Energy & Environmental Science* **11**, 1062-1176, doi:10.1039/c7ee02342a (2018).

8 Riahi, K., Rubin, E. S. & Schrattenholzer, L. Prospects for carbon capture and sequestration technologies assuming their technological learning. *Energy* **29**, 1309-1318, doi:10.1016/j.energy.2004.03.089 (2004).

9 Riahi, K., Rubin, E. S., Taylor, M. R., Schrattenholzer, L. & Hounshell, D. Technological learning for carbon capture and sequestration technologies. *Energy Economics* **26**, 539-564, doi:10.1016/j.eneco.2004.04.024 (2004).

10 Rubin, E. S., Taylor, M. R., Yeh, S. & Hounshell, D. A. Learning curves for environmental technology and their importance for climate policy analysis. *Energy* **29**, 1551-1559, doi:10.1016/j.energy.2004.03.092 (2004).

11 IEAGHG. Estimating the Future Trends in the Cost of CO_2 Capture Technologies. (IEA Greenhouse Gas R&D Programme, 2006).

12 Rochedo, P. R. R. & Szklo, A. Designing learning curves for carbon capture based on chemical absorption according to the minimum work of separation. *Applied Energy* **108**, 383-391, doi:10.1016/j.apenergy.2013.03.007 (2013).

13 Rubin, E. S., Yeh, S., Antes, M., Berkenpas, M. & Davison, J. Use of experience curves to estimate the future cost of power plants with CO2 capture. *International Journal of Greenhouse Gas Control* **1**, 188-197, doi:10.1016/S1750-5836(07)00016-3 (2007).

14 van den Broek, M., Hoefnagels, R., Rubin, E., Turkenburg, W. & Faaij, A. Effects of technological learning on future cost and performance of power plants with CO2 capture. *Progress in Energy and Combustion Science* **35**, 457-480, doi:10.1016/j.pecs.2009.05.002 (2009).

15 Li, S., Zhang, X., Gao, L. & Jin, H. Learning rates and future cost curves for fossil fuel energy systems with CO2 capture: Methodology and case studies. *Applied Energy* **93**, 348-356, doi:10.1016/j.apenergy.2011.12.046 (2012).

16 Kang, J. N. *et al.* The Prospects of Carbon Capture and Storage in China's Power Sector under the 2 °C Target: A Component-based Learning Curve Approach. *International Journal of Greenhouse Gas Control* **101**, doi: 10314910.1016/j.ijggc.2020.103149 (2020).

17 Zhou, L., Duan, M. S., Yu, Y. D. & Zhang, X. L. Learning rates and cost reduction potential of indirect coal-to-liquid technology coupled with CO2 capture. *Energy* **165**, 21-32, doi:10.1016/j.energy.2018.09.150 (2018).
