# Supplementary material for: Technological pathways for cost-effective steel decarbonization
Source: Nature. 2025 Oct 29;647(8088):93–101. doi: 10.1038/s41586-025-09658-9 (PMC12589104; doi:10.1038/s41586-025-09658-9)
Supplement: Supplementary file 2 — Reporting Summary [file 41586_2025_9658_MOESM2_ESM.pdf]

nature portfolio

Corresponding author(s): Jing Meng

Last updated by author(s): Sep 17, 2025

Reporting Summary

Nature Portfolio wishes to improve the reproducibility of the work that we publish. This form provides structure for consistency and transparency in reporting. For further information on Nature Portfolio policies, see our Editorial Policies and the Editorial Policy Checklist.

Statistics

For all statistical analyses, confirm that the following items are present in the figure legend, table legend, main text, or Methods section.

n/a	Confirmed	
☐	☒	The exact sample size (*n*) for each experimental group/condition, given as a discrete number and unit of measurement
☐	☒	A statement on whether measurements were taken from distinct samples or whether the same sample was measured repeatedly
☐	☒	The statistical test(s) used AND whether they are one- or two-sided<br>*Only common tests should be described solely by name; describe more complex techniques in the Methods section.*
☒	☐	A description of all covariates tested
☒	☐	A description of any assumptions or corrections, such as tests of normality and adjustment for multiple comparisons
☐	☒	A full description of the statistical parameters including central tendency (e.g. means) or other basic estimates (e.g. regression coefficient) AND variation (e.g. standard deviation) or associated estimates of uncertainty (e.g. confidence intervals)
☐	☒	For null hypothesis testing, the test statistic (e.g. *F*, *t*, *r*) with confidence intervals, effect sizes, degrees of freedom and *P* value noted<br>*Give P values as exact values whenever suitable.*
☒	☐	For Bayesian analysis, information on the choice of priors and Markov chain Monte Carlo settings
☒	☐	For hierarchical and complex designs, identification of the appropriate level for tests and full reporting of outcomes
☒	☐	Estimates of effect sizes (e.g. Cohen's *d*, Pearson's *r*), indicating how they were calculated

Our web collection on statistics for biologists contains articles on many of the points above.

Software and code

Policy information about availability of computer code

Data collection	Data collection was performed using open-source software R (version 4.4.3), RStudio (version 2024.12.1). Custom R scripts are openly available at https://doi.org/10.5281/zenodo.8214604.
Data analysis	Data analysis was performed using open-source software R (version 4.4.3), RStudio (version 2024.12.1), and Python (version 3.9.12). Custom R and Python scripts are openly available at https://doi.org/10.5281/zenodo.8214604.

For manuscripts utilizing custom algorithms or software that are central to the research but not yet described in published literature, software must be made available to editors and reviewers. We strongly encourage code deposition in a community repository (e.g. GitHub). See the Nature Portfolio guidelines for submitting code & software for further information.

Data

Policy information about availability of data

All manuscripts must include a data availability statement. This statement should provide the following information, where applicable:

- Accession codes, unique identifiers, or web links for publicly available datasets
- A description of any restrictions on data availability
- For clinical datasets or third party data, please ensure that the statement adheres to our policy

The dataset of cost-effective decarbonization pathways for global iron and steel plants is available at https://doi.org/10.5281/zenodo.8214604. It includes geographic locations and current technologies of iron and steel plants worldwide, along with plant-level data on cost-effective decarbonization technologies,

emission reductions, and abatement costs from 2020 to 2050 under three different carbon-neutrality strategies. Additionally, all references used for the systematic reviews on CCS and hydrogen costs and learning rates (Notes S9-S10) are provided. Data for the future production of iron and steel by country are available on the website of IEA ETP Clean Energy Technology Guide: https://www.iea.org/data-and-statistics/data-tools/etp-clean-energy-technology-guide.

Research involving human participants, their data, or biological material

Policy information about studies with human participants or human data. See also policy information about sex, gender (identity/presentation), and sexual orientation and race, ethnicity and racism.

Reporting on sex and gender	This study did not involve human participants or considerations of sex and gender.
Reporting on race, ethnicity, or other socially relevant groupings	This study did not involve human participants, nor did it include analyses of race, ethnicity, or other socially relevant groupings.
Population characteristics	This study did not involve human participants, and population characteristics.
Recruitment	This study did not involve human participants, and recruitment.
Ethics oversight	This study did not involve human participants, biological material, and ethics oversight.

Note that full information on the approval of the study protocol must also be provided in the manuscript.

Field-specific reporting

Please select the one below that is the best fit for your research. If you are not sure, read the appropriate sections before making your selection.

☐ Life sciences ☐ Behavioural & social sciences ☒ Ecological, evolutionary & environmental sciences

For a reference copy of the document with all sections, see nature.com/documents/nr-reporting-summary-flat.pdf

Ecological, evolutionary & environmental sciences study design

All studies must disclose on these points even when the disclosure is negative.

Study description	This study develops a model integrating global plant-level datasets and technology cost forecasts to identify the least-cost pathway for each steel plant worldwide, aligned with national carbon neutrality targets.
Research sample	The study draws on two plant-level databases: the World Crude Steel Capacity and Production Database (https://www.steelonthenet.com/plant.html) and the Global Iron and Steel Cost Database (https://www.transitionzero.org/products/global-steel-cost-tracker, http://www.metalinfo.cn). Together, these datasets cover more than 4,900 operating plants worldwide, comprising over 20,000 facilities in 127 countries. Of these, 1,967 plants are involved in the production of iron and crude steel, while the others are steel processing plants which are excluded from this study due to their limited emissions and lack of decarbonization measures.
Sampling strategy	The studied samples include all iron- and crude-steel-producing plants identified in the global plant-level databases. Steel processing plants were excluded from the analysis due to their limited emissions and the absence of relevant decarbonization measures. This approach ensures that the sample is representative of the global steel production fleet in terms of carbon mitigation potential.
Data collection	Plant-level data were obtained from existing commercial databases: the World Crude Steel Capacity and Production Database (https://www.steelonthenet.com/plant.html) and the Global Iron and Steel Cost Database (https://www.transitionzero.org/products/global-steel-cost-tracker, http://www.metalinfo.cn).
Timing and spatial scale	Plant-level annual production was recorded in 2018 in the World Crude Steel Capacity and Production Database and updated for 2020–2021 using World Steel Association data. Plant-specific costs were provided by the cost databases for 2021.
Data exclusions	The analysis focused exclusively on ironmaking and steelmaking plants. Steel processing plants in the databases were excluded, as they were not the target of this study and contribute negligible emissions without promising decarbonization options.
Reproducibility	All data and code necessary to reproduce the results of this study are available at https://doi.org/10.5281/zenodo.8214604.
Randomization	Randomization was not applicable, as this study used plant-level databases with comprehensive global coverage of iron- and steelmaking plants, rather than relying on randomized sampling.
Blinding	Blinding was not applicable, as this study relied on commercial plant-level databases and involved no experimental or observational participants.

Did the study involve field work? ☐ Yes ☒ No

Reporting for specific materials, systems and methods

We require information from authors about some types of materials, experimental systems and methods used in many studies. Here, indicate whether each material, system or method listed is relevant to your study. If you are not sure if a list item applies to your research, read the appropriate section before selecting a response.

Materials & experimental systems

n/a	Involved in the study
☒	☐ Antibodies
☒	☐ Eukaryotic cell lines
☒	☐ Palaeontology and archaeology
☒	☐ Animals and other organisms
☒	☐ Clinical data
☒	☐ Dual use research of concern
☒	☐ Plants

Methods

n/a	Involved in the study
☒	☐ ChIP-seq
☒	☐ Flow cytometry
☒	☐ MRI-based neuroimaging

Plants

Seed stocks	No seed stocks or other plant materials used.
Novel plant genotypes	This study did not involve plants or novel plant genotypes.
Authentication	This study did not involve seed stocks or novel plant genotypes.
